# Lack of membrane sex steroid receptors for mediating rapid endocrine responses in molluscan nervous systems

**DOI:** 10.3389/fendo.2024.1458422

**Published:** 2024-08-12

**Authors:** István Fodor, Shin Matsubara, Tomohiro Osugi, Akira Shiraishi, Tsuyoshi Kawada, Honoo Satake, Zsolt Pirger

**Affiliations:** ^1^ Ecophysiological and Environmental Toxicological Research Group, HUN-REN Balaton Limnological Research Institute, Tihany, Hungary; ^2^ Bioorganic Research Institute, Suntory Foundation for Life Sciences, Kyoto, Japan

**Keywords:** progesterone, testosterone, estradiol, membrane receptor, mollusk, *Lymnaea stagnalis*

## Abstract

Despite the lack of endogenous synthesis and relevant nuclear receptors, several papers have been published over the decades claiming that the physiology of mollusks is affected by natural and synthetic sex steroids. With scant evidence for the existence of functional steroid nuclear receptors in mollusks, some scientists have speculated that the effects of steroids might be mediated via membrane receptors (i.e. via non-genomic/non-classical actions) - a mechanism that has been well-characterized in vertebrates. However, no study has yet investigated the ligand-binding ability of such receptor candidates in mollusks. The aim of the present study was to further trace the evolution of the endocrine system by investigating the presence of functional membrane sex steroid receptors in a mollusk, the great pond snail (*Lymnaea stagnalis*). We detected sequences homologous to the known vertebrate membrane sex steroid receptors in the *Lymnaea* transcriptome and genome data: G protein-coupled estrogen receptor-1 (GPER1); membrane progestin receptors (mPRs); G protein-coupled receptor family C group 6 member A (GPRC6A); and Zrt- and Irt-like protein 9 (ZIP9). Sequence analyses, including conserved domain analysis, phylogenetics, and transmembrane domain prediction, indicated that the mPR and ZIP9 candidates appeared to be homologs, while the GPER1 and GPRC6A candidates seemed to be non-orthologous receptors. All candidates transiently transfected into HEK293MSR cells were found to be localized at the plasma membrane, confirming that they function as membrane receptors. However, the signaling assays revealed that none of the candidates interacted with the main vertebrate steroid ligands. Our findings strongly suggest that functional membrane sex steroid receptors which would be homologous to the vertebrate ones are not present in *Lymnaea*. Although further experiments are required on other molluscan model species as well, we propose that both classical and non-classical sex steroid signaling for endocrine responses are specific to chordates, confirming that molluscan and vertebrate endocrine systems are fundamentally different.

## 1 Introduction

Despite the lack of endogenous synthesis and relevant nuclear receptors (1–9), several papers have been published over the past 70 years showing that the physiology of mollusks is affected by natural and synthetic sex steroids occurring in the environment, though the robustness of the bioassay data in most of these papers has been questioned (10). Recent reviews tried to fill in the gaps in steroid signaling of mollusks and raised the idea that vertebrate sex steroids affect molluscan species via membrane receptors (i.e. non-genomic/non-classical actions) (4, 11).

Most efforts to prove this possible mode of action were performed by the investigation of hemocytes of *Mytilus* spp. [reviewed by (4)]. These works demonstrated that estradiol (E_2_) rapidly induced lysosomal membrane instability, production of intracellular reactive oxygen species, lysozyme release and nitrogen-oxide production in *Mytilus* hemocytes; the underlying mechanisms, similar to those identified in mammalian cells, involved cytosolic Ca^2+^ increase, activation of MAPKs and PKC, as well as phosphorylation of signal transducers and transcription factors (12–15). However, it is unknown whether E_2_ binds to a specific membrane receptor in mollusks, if it cross-reacts with a receptor for other compound(s), or if there is a cognate sex steroid receptor at all. Furthermore, to our knowledge, no study has investigated the potential rapid effects of progesterone (P) and testosterone (T) on mollusks or conducted homology searches for their known membrane receptors in mollusks. Although recent studies have revealed sequences homologous to membrane progestin receptor beta (mPRβ) and gamma (mPRγ), two of the five vertebrate mPRs (16), in molluscan species such as the cuttlefish *Sepia japonica* (17), owl limpet *Lottia gigantea*, and Pacific oyster *Crassostrea gigas* (18), the ability of these receptor candidates to bind P has not been examined.

The aim of the present paper was to advance our understanding of the evolution of the endocrine system and to contribute to the ongoing debate about the role of vertebrate-type sex steroids in mollusks, by investigating the great pond snail (*Lymnaea stagnalis*). *Lymnaea* has long-served as a multipurpose molluscan model in neuroscience and neuroendocrinology [reviewed by (19–24)], as well as in ecotoxicological studies related to endocrine disruption (25–28). To accomplish our aim, we searched for sequences in the *Lymnaea* transcriptome and genome data that show homology with the known vertebrate membrane sex steroid receptors that are not related to nuclear receptors: G protein-coupled estrogen receptor-1 (GPER1), five mPRs, G protein-coupled receptor family C group 6 member A (GPRC6A), and Zrt- and Irt-like protein 9 (ZIP9). We cloned the coding sequence of selected candidates and transiently expressed them in Human Embryonic Kidney (HEK) cells to assess their membrane expression and ability to bind vertebrate sex steroids. Our findings represent the first evidence indicating the absence of specific, evolutionarily conserved membrane receptor-mediated sex steroid signaling in a mollusk.

## 2 Materials and methods

### 2.1 Experimental animals

5-month-old adult, mature specimens of *Lymnaea* were obtained from our laboratory-bred stocks. Snails were maintained in large plastic tanks containing 10 L oxygenated artificial snail water (composition in mM: 0.1309 NaHCO_3_, 0.0378 K_2_SO_4_, 0.4013 CaCl_2_.2H_2_O, 0.0390 Mg(NO_3_)_2_.6H_2_O; pH=7.6) at 20°C (± 1°C) on a 12:12 h light:dark regime with natural wavelength light. Specimens were fed on lettuce *ad libitum* three times a week.

### 2.2 In silico searches and bioinformatic analysis

The neural transcriptome data obtained in our previous study (29) were used to search for sequences homologous to vertebrate membrane sex steroid receptors. For homology-searching, the relevant molluscan and vertebrate sequences were used as search queries (**Supplementary Table 1**). We also compared the obtained sequences with the public *Lymnaea* CNS transcriptome shotgun assembly (sequence read archive: #DRR002012 (30);) and genome data (31). Conserved domain search using NCBI CDD/SPARCLE (32, 33) was first performed to check if the key regions were present in the deduced protein sequence. Phylogenetic analysis was made in the Molecular Evolutionary Genetics Analysis v7 software (34). Transmembrane domain prediction was performed with the TMHMM 2.0 and DeepTMHMM tools (35).

### 2.3 Plasmid constructions and transfection

Plasmid construction and transfection was according to the description of our previous study (36). The complete coding sequence of GPER1, mPR, and ZIP9 candidates was amplified with PrimeStar HS DNA Polymerase (#R010A, TaKaRa Bio, Japan). The applied gene-specific primers (designed with the SnapGene Viewer software (GSL Biotech)) with extensions to vector ends were as follows: 1) GPER1 candidate: AAT GCG GCC GCA TGG AGA CCA CAG TCG GGG (forward) and GCC TCT AGA CAG ATG GAC TGG TGG TTT CAG AAA GCC AT (reverse); 2) mPR candidate: AAT GCG GCC GCA TGT TGT TAC TGC CAG CAA CAT TGA G (forward) and GCC TCT AGA ATG GAT GAC CCC ATT TCT GTG ATG AA (reverse); 3) ZIP9 candidate: AAT GCG GCC GCA TGG ATG ATA TCT TGA CTC TCC TTT CTC TGT (forward) and GCC TCT AGA GTG CTT ATG ACC CAC AGC TAA AAA AAC A (reverse) (Integrated DNA Technologies, Belgium).

The amplicons were checked by gel electrophoresis, restricted with NotI – XbaI enzymes, purified by gel electrophoresis, and subcloned into the linearized pcDNA6-His-G16 plasmid [described in (37)] using T4 DNA ligase (#LGK-201, Toyobo, Japan). Since the coding sequence of GPRC6A candidate was too long to effectively amplify it, it was synthetized and ordered in pcDNA6-His-G16 plasmid as a ready-to-use vector (Eurofins Genomics). Codon usage optimized mPR and ZIP9 candidates in pcDNA6-His-G16 plasmids were synthesized by FASMAC (Kanagawa, Japan). After transformation and amplification in JM109 competent cells, colony PCR was carried out. Next, the plasmids were purified from the appropriate colonies with the QIAGEN Plasmid Midi Kit (#12143, Qiagen) and sequence verification by nucleotide sequencing was performed.

Finally, the constructed vectors were transiently transfected into genetically engineered HEK293MSR cells using the Lipofectamine 3000 Transfection reagent (#L3000001, Thermo Fisher Scientific) following the manufacturer’s instructions. Transfection efficiency and expression for all candidates were evaluated by an immunodetection method as described below.

### 2.4 Fluorescence immunocytochemistry

Immunocytochemistry was performed according to the description of our previous studies (36, 38). Briefly, 5 μg of the constructed pcDNA6-His-G16 vectors containing the receptor candidates were transfected into HEK293MSR cells in glass bottom dishes (35 mm diameter). As method controls, 5 μg of pcDNA6-His-G16 vectors containing GFP or *Ciona* galanin-like peptide receptor (GALPR) (37) were also transfected into HEK293MSR cells. After blocking with 4% goat serum (#S-1000-20, Vector Laboratories, USA), immunoreactions were performed using an anti-mouse G16 antibody (1:50; #TA808136, Origene, USA). The secondary antibody was a goat anti-mouse IgG antibody conjugated with Alexa488 antibody (1:500; #A-11029, Thermo Fisher Scientific). Nuclei were stained with Hoechst 33342 (1:1000; #AS-83218, AnaSpec, USA). The immunostaining was analyzed with a Fluoview FV3000 confocal laser microscope (Olympus, Tokyo, Japan) equipped with appropriate wavelength-filter configuration settings and a transmitted light detector. Image processing was performed by the Fluoview software (Olympus).

### 2.5 Second messenger assays

Intracellular Ca^2+^ mobilization was following the description of our previous studies (36, 38). Briefly, 1 × 10^6^ HEK293MSR cells were cultured on polystyrene culture dishes (100 mm in diameter). A day later, 10 μg of pcDNA6-His-G16 vectors containing the receptor candidates or empty pcDNA6-His-G16 vector (i.e. Mock) were transfected into the HEK293MSR cells. After incubating for 24 h, 6 × 10^5^ cells were plated in each of a 96-well plate. For the assays, synthetic P (#5341; Merck), T (#86500; Merck), and E_2_ (#E8875; Merck) were used. The stock solutions were prepared in ethanol weekly. In the positive control experiment, *Ciona* GALP (PFRGQGGWTLNSVGYNAGLGALRKLFE (39);) was applied. The real-time fluorescence assessment of Ca^2+^ mobilization was performed using the FLIPR Calcium 5 Kit (#3808451, Molecular Devices, USA) following the manufacturer’s instructions. All measurements were performed with a FlexStation III Multi Mode microplate reader (Molecular Devices). The data were obtained at least from two independent transfections for *Ciona* GALPR, all *Lymnaea* receptor candidates, and the empty vector. *Ciona* GALPR and empty vector samples were applied to the plate in two technical replicates, while all *Lymnaea* receptor candidate samples were applied in three technical replicates.

### 2.6 Statistical analysis

The data obtained during the second messenger assays were plotted using four-parameters logistics-based non-linear regression curve fit feature in the GraphPad Prism5 software (GraphPad, San Diego, USA). Significant differences at a given data point was analyzed with the OriginPro 2018 software (OriginLab Corp., USA). The normality of the datasets was investigated using the Shapiro-Wilk test and the homogeneity of variances between groups was checked with the Levene-test. The data were then analyzed using the two-sample t-test.

## 3 Results

Our homology-searching revealed candidate sequences for all vertebrate membrane sex steroid receptors which are not related to the nuclear ones (**Supplementary Figure 1**). After the initial bioinformatic analysis, including conserved domain analysis (**Supplementary Figure 2**), phylogenetics (**Supplementary Figures 3-5**), and transmembrane domain prediction (**Supplementary Figure 6**), four sequences were investigated further: one GPER1 candidate, one mPR candidate, one GPRC6A candidate, and one ZIP9 candidate. Based on the *in silico* analyses, the mPR and ZIP9 candidates appeared to be genuine homologs. Although the GPER1 and GPRC6A candidates were unlikely to be the counterparts of the vertebrate receptors, we did not exclude the possibility that, during evolution, these deduced proteins might have acquired the ability of recognizing steroids as well [as reported in the case of vertebrate ZIP9 (40)].

**Figure 1:**
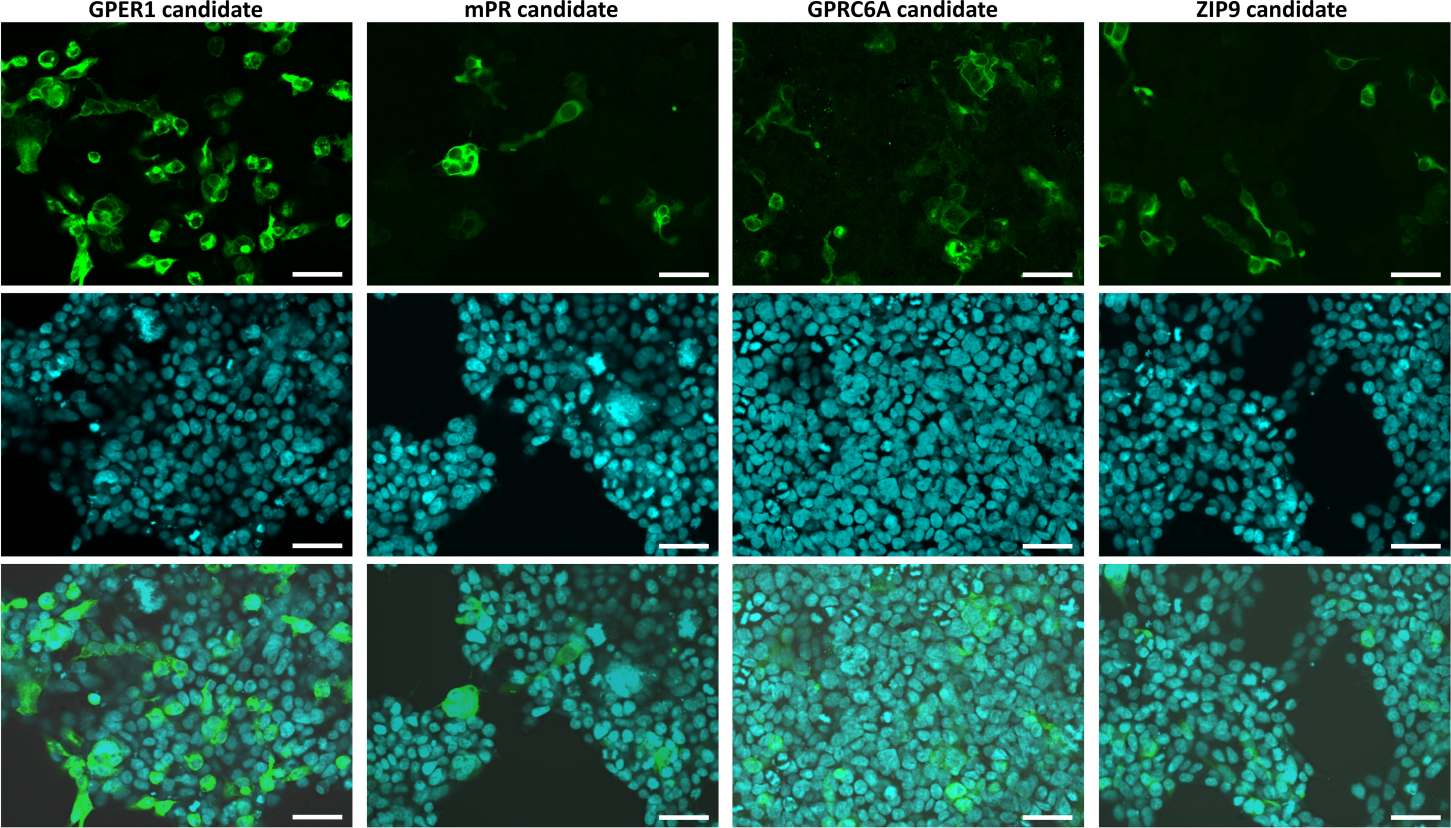
Confocal microscopic observation of immunostained HEK293MSR cells expressing the different *Lymnaea* membrane sex steroid receptor candidates fused with G16 protein. Expressed candidates stained with an anti-G16 antibody (green; top row), nuclei stained with Hoechst 33342 (blue; middle row), and merged views (bottom row) of the transfectants. The candidates were localized at the plasma membrane. The expression of the GPER1 and GPRC6A candidates were high, while the expression of the mPR1 and ZIP9 candidates were very low (only a few cells showing immunosignal were detected). Bars = 50 μm.

**Figure 2:**
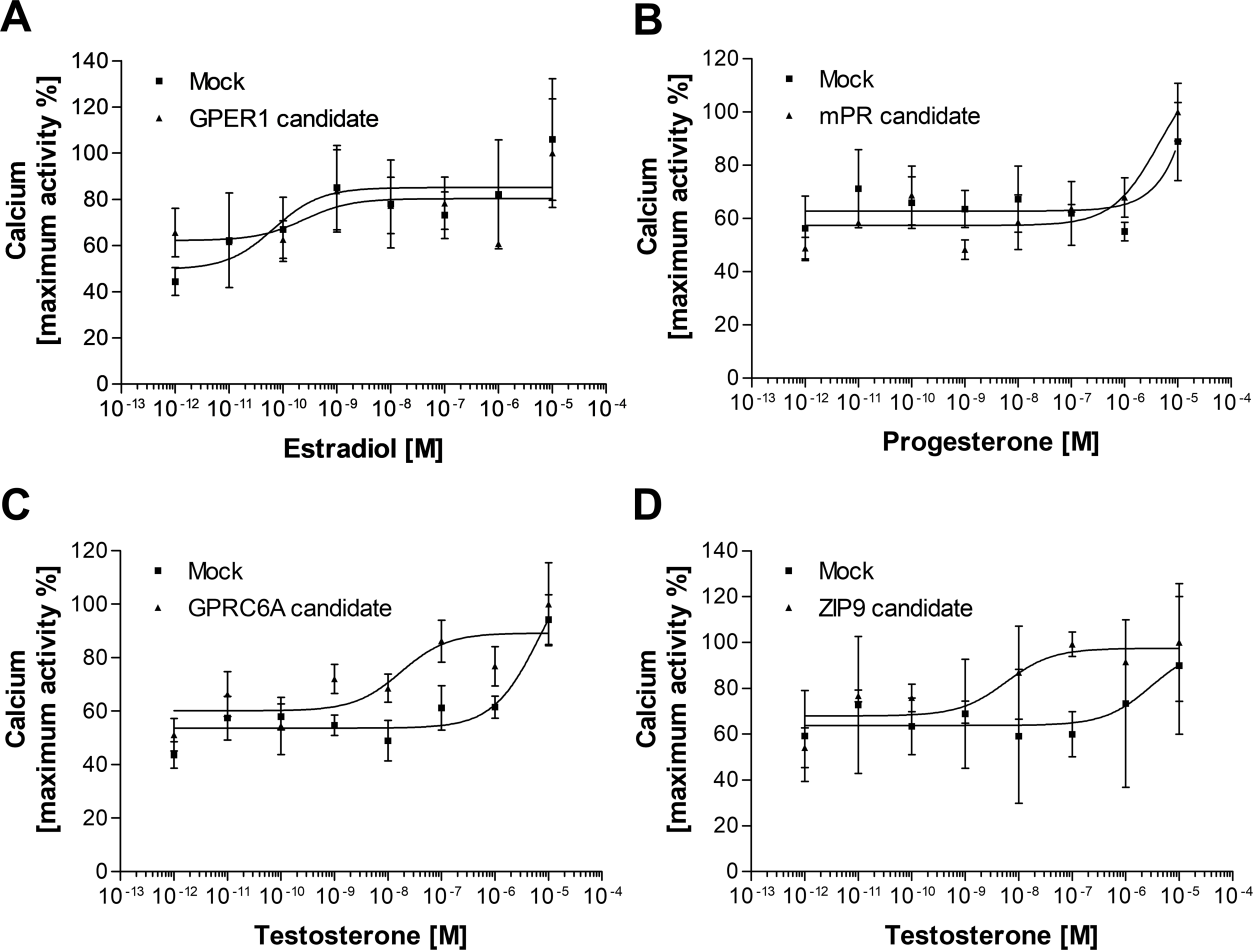
Second messenger assay (Ca^2+^ mobilization) for assessing the potential ligand-receptor interaction between vertebrate type-sex steroids and *Lymnaea* membrane sex steroid receptor candidates. Dose-response curve of E_2_ in GPER1 candidate-expressing HEK cells **(A)**, P in mPR candidate-expressing cells **(B)**, and T in GPCR6A candidate or ZIP9 candidate-expressing HEK cells **(C, D)**. Empty vector was transfected as the negative control (Mock). None of the candidates were responsive to the main steroid ligand. Data points are means ± SEM of two **(A, B, D)** or three **(C)** independent transfections.

First, we investigated the expression and subcellular localization of the candidates by transiently transfecting the G16-fused coding sequences into HEK293MSR cells. In the positive control experiments (**Supplementary Figure 7**), the expression level of GFP indicated an appropriate transfection efficiency (**Supplementary Figure 7A**). Moreover, the G16-fused *Ciona* GALPR showed a high expression and localization at the plasma membrane (**Supplementary Figure 7B**). Immunocytochemical fluorescence confocal microscopic detection confirmed that the *Lymnaea* membrane sex steroid receptor candidates were localized to the cell surface of HEK293MSR cells (**Figure 1**). The expression of the GPER1 and GPRC6A candidates was high. However, the expression of the mPR1 and ZIP9 candidates was very low and only a few cells showing immunosignal were detected. To evaluate the ligand-receptor interaction between the three main vertebrate sex steroids and the *Lymnaea* membrane sex steroid receptor candidates, we assessed the mobilization of intracellular Ca^2+^ using G16-fused receptor candidates (41). In the positive control experiment (**Supplementary Figure 7C**), the *Ciona* GALP specifically induced the elevation of intracellular Ca^2+^ in a concentration-dependent manner, as previously reported (37). In the case of the *Lymnaea* candidates, however, the assay showed that the respective ligand-receptor combinations failed to induce the elevation of intracellular Ca^2+^ (**Figure 2**), indicating that these transmembrane proteins are not *bona fide* membrane sex steroid receptors. Both in the case of GPRC6A and ZIP9 candidates, there was an increase in response at the 10^-7^ M concentration, but statistical analysis with two-sample t-test revealed no significant difference compared to the response of HEK cells expressing the empty vector.

**Figure 3:**
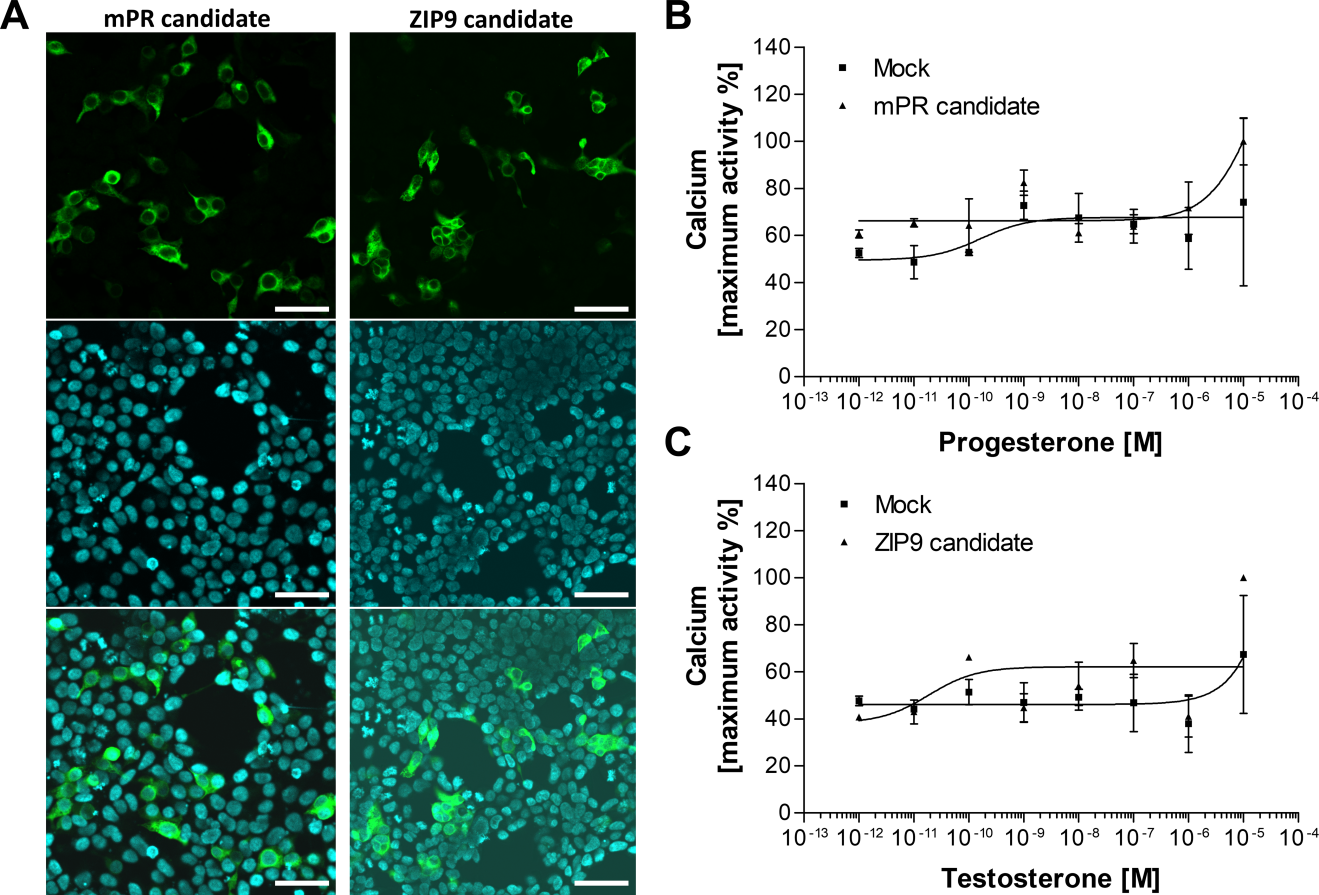
Confocal microscopic observation of immunostained HEK293 cells expressing codon usage optimized mPR and ZIP9 candidates fused with G16 protein **(A)** and functional receptor assay for assessing ligand-binding **(B, C)**. **(A)** Expressed receptor candidates stained with anti-G16 antibody (green), nuclei stained with Hoechst 33342 (blue), and merged views of the transfectants. The candidates were localized at the plasma membrane. The expression of both candidates significantly improved due to the codon usage optimization. Bars = 50 μm. Dose-response curve of P in mPR candidate-expressing cells **(B)** and T in ZIP9 candidate-expressing HEK cells **(C)**. Empty vector was transfected as the negative control (Mock). Both candidates were unresponsive to the main steroid ligand. Data points are means ± SEM of two independent transfections.

We did not exclude the possibility that the mPR and ZIP9 homologues were unresponsive due to their low expression. Hence, we applied codon usage optimization for both sequences (**Supplementary Figure 8**) and investigated their expression and ligand-binding interaction again (**Figure 3**). Although the expression of both candidates was improved after the codon usage optimization (**Figure 3A**), they were still found to be unresponsive to vertebrate sex steroids (**Figures 3B, C**).

## 4 Discussion

There is a long-standing debate regarding whether vertebrate sex steroids can affect the physiology of mollusks. Sex steroids are present in tissues of a wide range of mollusks, and their concentration sometimes show an association with stages of gonad development or differences between tissues and sexes [reviewed by (1)]. However, since the 2000s, it has become evident that mollusks possess a remarkable ability to absorb and retain vertebrate sex steroids for extended periods, even months (42–51). Consequently, their presence in molluscan tissues does not necessarily indicate an endogenous origin. Given that vertebrate sex steroids have been present in the aquatic environment - and thus in molluscan tissues - for a considerable part of molluscan evolutionary history, the question arises as to whether evolution has favored the development of signaling systems to recognize these compounds.

**Figure 4:**
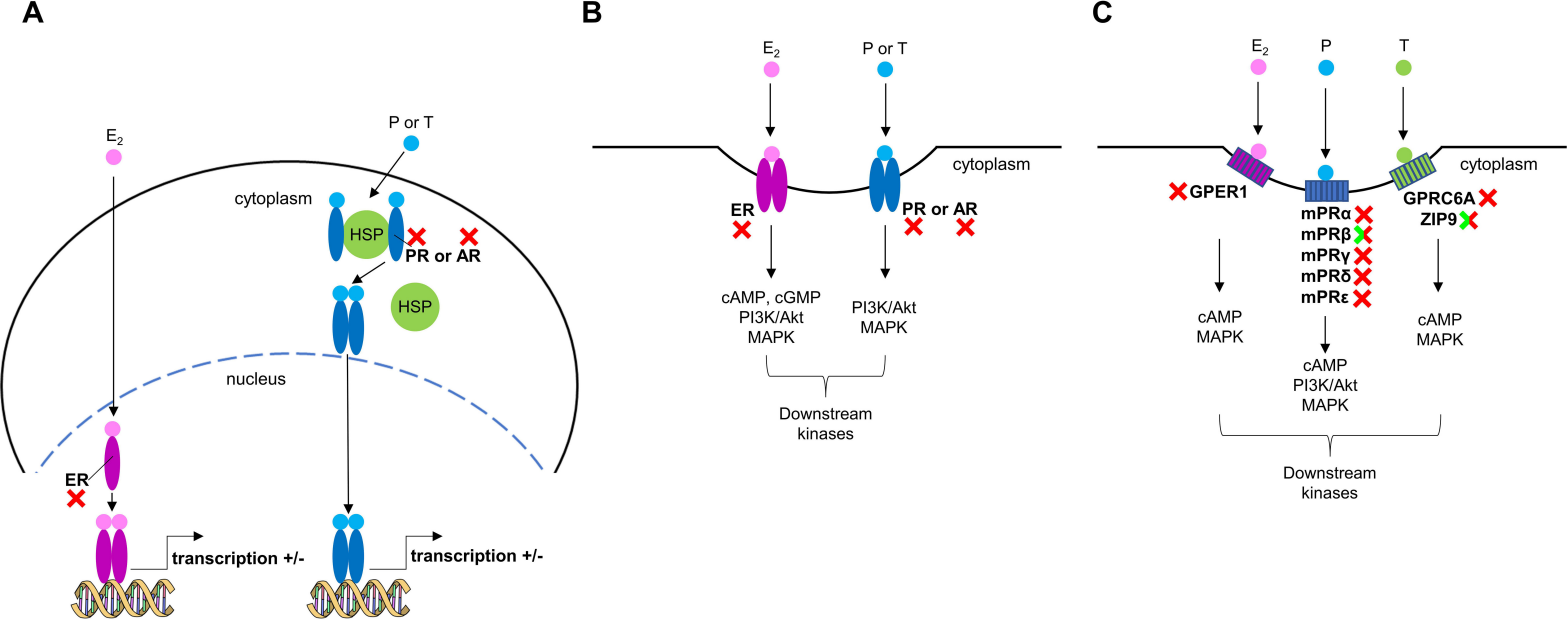
Sex steroid signaling in vertebrates. **(A)** Classic nuclear pathway involving estrogen (ER), progesterone (PR), and androgen (AR) receptors, which are primarily located as monomers in the nucleus or as monomers bound to heat shock proteins (HSPs) in the cytoplasm. After steroid binding, dimerization, changes in receptor conformation (and nuclear localization), the receptors bind to specific steroid-response elements (SREs) to modulate gene transcription. **(B)** The membrane forms of ER, PR, and AR are tethered to the cell membrane and can also bind sex steroids activating G proteins (e.g., Gs) and/or kinases (e.g., PI3K/Akt pathway). **(C)** Membrane sex steroid receptors (GPCRs and non-GPCRs) which are unrelated to ER, PR, and AR can also bind sex steroids activating G proteins and kinases. Red X symbols mark vertebrate receptors which have no homologs in *Lymnaea*, while green/red X symbols indicate vertebrate receptors which have structural but, in terms of the sex steroid signaling, not functional homologs in *Lymnaea*.

Sex steroid signaling in vertebrates is regulated via three possible pathways (**Figure 4**). First, through nuclear receptors localized in the cytoplasm or nucleus (**Figure 4A**) (52). Second, following post-translational modification, the nuclear receptors relocate to the plasma membrane to facilitate the rapid effects of sex steroids (**Figure 4B**) (52, 53). Considering the absence of relevant functional nuclear receptors in mollusks, the nuclear receptor-mediation of sex steroids via these pathways can be excluded. Previous screenings of *Lymnaea* genome and transcriptome data did not identify functional nuclear sex steroid receptors either (2, 31, 54). We are careful to use the word ‘functional’ in this context, as molluscan genomes all contain a gene that was initially annotated as being a homologue of the vertebrate nuclear estrogen receptor (nER), although it is actually orthologous to the common ancestor of vertebrate estrogen and oxosteroid receptors (55). Moreover, all studies on mollusks to date, except one (56), have shown that molluscan nERs do not bind to E_2_ and are, in fact, in an already activated state (57–59). Previously, the nER of the scallop was reported to be bind to E_2_ (56). Nevertheless, the hybrid vector used in that study was made up of fish and scallop sequences, and the binding evidence was a two-fold increase at one dose of E_2_. Hence, we would argue that data are insufficient to refer to the scallop nER as a ‘functional’ E_2_ receptor and support the proposal of (60) that sequences previously termed molluscan nER should be renamed NR3D. The third possible pathway involves rapid effects mediated by membrane receptors which are distinct from nuclear receptors (**Figure 4C**) (16, 40, 61–63). Given that GPCRs trace their origins back into history over one billion years, whereas the original ancestral nuclear steroid receptor appeared about 500-600 million years ago (7), other works (4, 11) have very reasonably suggested that sex steroids affect the physiology of mollusks through this mechanism.

The rapid effects of E_2_ in vertebrates are mediated by a single GPCR called GPER1 [for detailed reviews, see (62, 63)]. Our candidate was unresponsive to E_2_, suggesting that no GPER1 homolog is present in *Lymnaea*. The identified sequence likely belongs to the protostome allatostatin-C receptors, which are closely related to echinoderm somatostatin/allatostatin-C-type receptors and chordate somatostatin-type and opioid-type receptors (**Supplementary Figure 3**) (64, 65). Without assessing the robustness of previously reported rapid effects of E_2_ on *Mytilus* hemocytes, we note that only the lysosomal membrane stability-decreasing effect has so far been properly verified. This classic ‘immune response’, as also referenced by the authors of the original study (4), led to the hypothesis the E_2_ may act as a xenobiotic ‘inflammatory agent’ for mollusks. Consistent with the previous idea that recognition and deactivation of xenobiotics were significant evolutionary pressures for receptor development (66), we hypothesize that mollusks have an unknown specific membrane receptor, though not a GPER1 homolog, capable of binding E_2_ and initiating a rapid immune response.

Rapid actions of P are implemented by five mPR subtypes, which are not GPCRs but belong to the progestin and adipoQ receptor (PAQR) family, in vertebrates [for recent detailed reviews, see (16, 61)]. The previous scenario was that mPRs are exclusive to chordates (67). However, a recent comprehensive genomic analysis of metazoans revealed homologs for mPRβ and mPRγ in bivalves, suggesting that the five vertebrate mPRs evolved from an ancestral metazoan mPRγ (18). It is also noteworthy that mPRα, which has been shown to mediate most mPR functions in vertebrate cells (even when they express all isoforms), is absent in invertebrates. Our *in silico* revealed only one potential mPR candidate in *Lymnaea*. Conservation analysis of this sequence yielded similar results to those previously reported in mollusks (**Supplementary Figures 9**, **10**) (17). However, our ligand-receptor interaction experiment showed that this candidate did not respond to P, suggesting the absence of an mPR homolog in *Lymnaea*. Adiponectin receptor, a member of PAQR family, was recently identified and showed to be involved in learning and memory formation of *Lymnaea* (68). Our identified sequence may also belong to the PAQR family (**Supplementary Figure 4**) and use adiponectin as its ligand, but further studies are required to confirm this assumption. Collectively, the present study supports the original theory that functional mPRs are exclusive to chordates.

Non-genomic effects of T in vertebrates are mediated by two proteins, GPRC6A (GPCR) and ZIP9 (non-GPCR) [reviewed by (40, 69)]. Our homology-searches revealed one candidate for both proteins in *Lymnaea*, but neither responded to T, suggesting the absence of mAR homologs in this species. GPRC6A is also known to be activated by L-α-amino acids (40). Based on our *in silico* analyses (**Supplementary Figures 2**, **5**), we suppose that the identified *Lymnaea* sequence functions as a metabotropic glutamate receptor, although experimental validation awaits further studies. In vertebrate cells, ZIP9 has dual functions as a zinc transporter and as a mAR activating second messengers (40). Since *Lymnaea* ZIP9 was unresponsive to T, we hypothesize that its sole function is to participate in zinc homeostasis.

In summary, our findings strongly suggest that functional homologs of vertebrate membrane sex steroid receptors are not present in *Lymnaea*. Although further experiments are required on other molluscan model species as well, we propose that both classical and non-classical sex steroid signaling for endocrine responses are specific to chordates. In light of this, it remains an important and unanswered question how vertebrate steroids affect the physiology of mollusks. Vertebrate sex steroids are probably xenobiotic ‘inflammatory agents’ for mollusks and may be recognized by membrane receptors involved in rapid immune responses. Another potential target for endocrine disruption by vertebrate steroids in mollusks might be an ecdysone-like pathway. Ecdysone signaling was long thought to be exclusive to arthropods; however, studies from 2010 have demonstrated the presence of an orthologue of the EcR/NR1H receptor in other protostome taxa, including mollusks and annelids (70). In insects, 20-hydroxyecdysone exerts both genomic and non-genomic actions (71). It is noteworthy that GPCR-mediated effects of 20-hydroxyecdysone have also been described in mammals, demonstrating the activation of an endogenous signaling pathway by a clearly exogenous steroid (72). Our homology searches revealed *Lymnaea* sequences homologous to both insect nuclear and membrane (GPCR) ecdysone receptors (**Supplementary Figure 11**), paving the way for future studies aimed at investigating this potential target pathway. Our findings have implications not only for the fields of endocrinology and evolution but also for ecology: it is important to consider the signaling pathways and responsiveness of mollusks to vertebrate sex steroids when assessing the impact of these compounds on ecosystems.

## Data Availability

The datasets presented in this study can be found in online repositories. The names of the repository/repositories and accession number(s) can be found in the article/**Supplementary Material**.

## References

[1] Scott AP. Do mollusks use vertebrate sex steroids as reproductive hormones? Part I. Critical appraisal of the evidence for the presence, biosynthesis and uptake of steroids. Steroids. (2012) 77:1450–68. doi: 10.1016/j.steroids.2012.08.009 22960651

[2] Fodor I Urban P Scott AP Pirger Z. A critical evaluation of some of the recent so-called 'evidence' for the involvement of vertebrate-type sex steroids in the reproduction of mollusks. Mol Cell Endocrinol. (2020) 516:110949. doi: 10.1016/j.mce.2020.110949 32687858

[3] Horiguchi T Ohta Y. A critical review of sex steroid hormones and the induction mechanisms of imposex in gastropod mollusks. In: Saber Saleuddin ABL Orchard I, editors. Advances in invertebrate (Neuro)Endocrinology, 1st edition. Boca Raton, Florida, USA: Apple Academic Press (2020).

[4] Balbi T Ciacci C Canesi L. Estrogenic compounds as exogenous modulators of physiological functions in molluscs: Signaling pathways and biological responses. Comp Biochem Physiol C Toxicol Pharmacol. (2019) 222:135–44. doi: 10.1016/j.cbpc.2019.05.004 31055067

[5] Fodor I Pirger Z. From dark to light - an overview of over 70 years of endocrine disruption research on marine mollusks. Front Endocrinol. (2022) 13:903575. doi: 10.3389/fendo.2022.903575 PMC9301197 35872980

[6] Markov GV Tavares R Dauphin-Villemant C Demeneix BA Baker ME Laudet V. Independent elaboration of steroid hormone signaling pathways in metazoans. Proc Natl Acad Sci USA. (2009) 106:11913–8. doi: 10.1073/pnas.0812138106 PMC2715501 19571007

[7] Markov GV Gutierreze-Mazariegos J Pitrat D Billas IML Bonneton F Moras D. Origin of an ancient hormone/receptor couple revealed by resurrection of an ancestral estrogen. Sci Adv. (2017) 3:e1601778. doi: 10.1126/sciadv.1601778 28435861 PMC5375646

[8] Baker ME. Steroid receptors and vertebrate evolution. Mol Cell Endocrinol. (2019) 496:110526. doi: 10.1016/j.mce.2019.110526 31376417

[9] Fernandes D Loi B Porte C. Biosynthesis and metabolism of steroids in molluscs. J Steroid Biochem Mol Biol. (2011) 127:189–95. doi: 10.1016/j.jsbmb.2010.12.009 21184826

[10] Scott AP. Do mollusks use vertebrate sex steroids as reproductive hormones? II. Critical review of the evidence that steroids have biological effects. Steroids. (2013) 78:268–81. doi: 10.1016/j.steroids.2012.11.006 23219696

[11] Tran TKA Yu RMK Islam R Nguyen THT Bui TLH Kong RYC. The utility of vitellogenin as a biomarker of estrogenic endocrine disrupting chemicals in molluscs. Environ pollut. (2019) 248:1067–78. doi: 10.1016/j.envpol.2019.02.056 31091639

[12] Canesi L Ciacci C Lorusso LC Betti M. Effects of endocrine disrupting chemicals on the immune system of the edible bivalve mollusc mytilus. In: Marino M Mita DG, editors. The Endocrine Disruptors. Trivandrum, India: Transworld Research Network (2007). p. 1–12.

[13] Canesi L Ciacci C Lorusso LC Betti M Guarnieri T Tavolari S. Immunomodulation by 17beta-estradiol in bivalve hemocytes. Am J Physiol Regul Integr Comp Physiol. (2006) 291:R664–73. doi: 10.1152/ajpregu.00139.2006 16601263

[14] Canesi L Lorusso LC Ciacci C Betti M Rocchi M Pojana G. Immunomodulation of *Mytilus* hemocytes by individual estrogenic chemicals and environmentally relevant mixtures of estrogens: in *vitro* and in *vivo* studies. Aquat Toxicol. (2007) 81:36–44. doi: 10.1016/j.aquatox.2006.10.010 17126923

[15] Canesi L Ciacci C Betti M Lorusso LC Marchi B Burattini S. Rapid effects of 17beta-estradiol on cell signaling and function of *Mytilus* hemocytes. Gen Comp Endocrinol. (2004) 136:58–71. doi: 10.1016/j.ygcen.2003.12.003 14980797

[16] Thomas P Pang Y Camilletti MA Castelnovo LF. Functions of membrane progesterone receptors (mPRs, PAQRs) in nonreproductive tissues. Endocrinology. (2022) 163:bqac147. doi: 10.1210/endocr/bqac147 36041040

[17] Pang Z Lu Z Wang M Gong L Liu B Jiang L. Characterization, relative abundances of mRNA transcripts, and subcellular localization of two forms of membrane progestin receptors (mPRs) in the common Chinese cuttlefish, *Sepiella japonica* . Anim Reprod Sci. (2019) 208:106107. doi: 10.1016/j.anireprosci.2019.106107 31405483

[18] Ren J Chung-Davidson YW Jia L Li W. Genomic sequence analyses of classical and non-classical lamprey progesterone receptor genes and the inference of homologous gene evolution in metazoans. BMC Evol Biol. (2019) 19:136. doi: 10.1186/s12862-019-1463-7 31262250 PMC6604198

[19] Fodor I Hussein AA Benjamin PR Koene JM Pirger Z. The unlimited potential of the great pond snail, *Lymnaea stagnalis* . eLife. (2020) 9:e56962. doi: 10.7554/eLife.56962 32539932 PMC7297532

[20] Rivi V Benatti C Colliva C Radighieri G Brunello N Tascedda F. *Lymnaea stagnalis* as model for translational neuroscience research: From pond to bench. Neurosci Biobehav Rev. (2020) 108:602–16. doi: 10.1016/j.neubiorev.2019.11.020 31786320

[21] Rivi V Benatti C Lukowiak K Colliva C Alboni S Tascedda F. What can we teach *Lymnaea* and what can *Lymnaea* teach us? Biol Rev Camb Philos Soc. (2021) 96:1590–602. doi: 10.1111/brv.1271622 PMC9545797 33821539

[22] Kemenes G Benjamin PR. Lymnaea . Curr Biol. (2009) 19:R9–11. doi: 10.1016/j.cub.2008.10.013 19138593

[23] Koene JM. Neuro-endocrine control of reproduction in hermaphroditic freshwater snails: mechanisms and evolution. Front Behav Neurosci. (2010) 4:167. doi: 10.3389/fnbeh.2010.00167 21088700 PMC2981420

[24] Nakai J Chikamoto N Fujimoto K Totani Y Hatakeyama D Dyakonova VE. Insulin and memory in invertebrates. Front Behav Neurosci. (2022) 16:882932. doi: 10.3389/fnbeh.2022.882932 35558436 PMC9087806

[25] Zrinyi Z Maasz G Zhang L Vertes A Lovas S Kiss T. Effect of progesterone and its synthetic analogs on reproduction and embryonic development of a freshwater invertebrate model. Aquat Toxicol. (2017) 190:94–103. doi: 10.1016/j.aquatox.2017.06.029 28697460

[26] Giusti A Lagadic L Barsi A Thome JP Joaquim-Justo C Ducrot V. Investigating apical adverse effects of four endocrine active substances in the freshwater gastropod *Lymnaea stagnalis* . Sci Total Environ. (2014) 493:147–55. doi: 10.1016/j.scitotenv.2014.05.130 24950493

[27] Giusti A Leprince P Mazzucchelli G Thome JP Lagadic L Ducrot V. Proteomic analysis of the reproductive organs of the hermaphroditic gastropod *Lymnaea stagnalis* exposed to different endocrine disrupting chemicals. PloS One. (2013) 8:e81086. doi: 10.1371/journal.pone.0081086 24363793 PMC3867191

[28] Svigruha R Fodor I Padisak J Pirger Z. Progestogen-induced alterations and their ecological relevance in different embryonic and adult behaviours of an invertebrate model species, the great pond snail (*Lymnaea stagnalis*). Environ Sci pollut Res Int. (2021) 28:59391–402. doi: 10.1007/s11356-020-12094-z PMC8542004 33349911

[29] Pirger Z Urban P Galik B Kiss B Tapodi A Schmidt J. Same same, but different: exploring the enigmatic role of the pituitary adenylate cyclase- activating polypeptide (PACAP) in invertebrate physiology. J Comp Physiol A Neuroethol Sens Neural Behav Physiol. (2024). doi: 10.1007/s00359-024-01706-5 PMC11551080 38940930

[30] Sadamoto H Takahashi H Okada T Kenmoku H Toyota M Asakawa Y. *De novo* sequencing and transcriptome analysis of the central nervous system of mollusc *Lymnaea stagnalis* by deep RNA sequencing. PloS One. (2012) 7:e42546. doi: 10.1371/journal.pone.0042546 22870333 PMC3411651

[31] Koene JM Jackson DJ Nakadera Y Cerveau N Madoui MA Noel B. The genome of the simultaneously hermaphroditic snail Lymnaea stagnalis reveals an evolutionary expansion of FMRFamide-like receptors. Res Square Preperint. (2024). doi: 10.21203/rs.3.rs-3948809/v1 PMC11589774 39587195

[32] Lu S Wang J Chitsaz F Derbyshire MK Geer RC Gonzales NR. CDD/SPARCLE: the conserved domain database in 2020. Nucleic Acids Res. (2020) 48:D265–D8. doi: 10.1093/nar/gkz991 PMC6943070 31777944

[33] Marchler-Bauer A Bo Y Han L He J Lanczycki CJ Lu S. CDD/SPARCLE: functional classification of proteins via subfamily domain architectures. Nucleic Acids Res. (2017) 45:D200–D3. doi: 10.1093/nar/gkw1129 PMC5210587 27899674

[34] Kumar S Stecher G Tamura K. MEGA7: molecular evolutionary genetics analysis version 7.0 for bigger datasets. Mol Biol Evol. (2016) 33:1870–4. doi: 10.1093/molbev/msw054 PMC8210823 27004904

[35] Hallgren J Tsirigos KD Pedersen MD Armenteros JJA Marcatili P Nielsen H. DeepTMHMM predicts alpha and beta transmembrane proteins using deep neural networks. BioRxiv. (2022). doi: 10.1101/2022.04.08.487609

[36] Nagasawa K Matsubara S Satake H Osada M. Functional characterization of an invertebrate gonadotropin-releasing hormone receptor in the Yesso scallop *Mizuhopecten yessoensis* . Gen Comp Endocrinol. (2019) 282:113201. doi: 10.1016/j.ygcen.2019.06.005 31199924

[37] Shiraishi A Okuda T Miyasaka N Osugi T Okuno Y Inoue J. Repertoires of G protein-coupled receptors for *Ciona*-specific neuropeptides. Proc Natl Acad Sci USA. (2019) 116:7847–56. doi: 10.1073/pnas.1816640116 PMC6475428 30936317

[38] Matsubara S Shiraishi A Sakai T Okuda T Satake H. Heterodimerization of the prostaglandin E_2_ receptor EP_2_ and the calcitonin receptor CTR. PloS One. (2017) 12:e0187711. doi: 10.1371/journal.pone.0187711 29095955 PMC5667882

[39] Kawada T Ogasawara M Sekiguchi T Aoyama M Hotta K Oka K. Peptidomic analysis of the central nervous system of the protochordate, *Ciona intestinalis*: homologs and prototypes of vertebrate peptides and novel peptides. Endocrinology. (2011) 152:2416–27. doi: 10.1210/en.2010-1348 21467196

[40] Thomas P. Membrane androgen receptors unrelated to nuclear steroid receptors. Endocrinology. (2019) 160:772–81. doi: 10.1210/en.2018-00987 30753403

[41] Milligan G Marshall F Rees S. G16 as a universal G protein adapter: implications for agonist screening strategies. Trends Pharmacol Sci. (1996) 17:235–7. doi: 10.1016/0165-6147(96)10026-2 8756181

[42] Schwarz TI Katsiadaki I Maskrey BH Scott AP. Mussels (*Mytilus* spp.) display an ability for rapid and high capacity uptake of the vertebrate steroid, estradiol-17beta from water. J Steroid Biochem Mol Biol. (2017) 165:407–20. doi: 10.1016/j.jsbmb.2016.08.007 27568213

[43] Schwarz TI Katsiadaki I Maskrey BH Scott AP. Rapid uptake, biotransformation, esterification and lack of depuration of testosterone and its metabolites by the common mussel, *Mytilus* spp. J Steroid Biochem Mol Biol. (2017) 171:54–65. doi: 10.1016/j.jsbmb.2017.02.016 28245981

[44] Fodor I Schwarz T Kiss B Tapodi A Schmidt J Cousins ARO. Studies on a widely-recognized snail model species (*Lymnaea stagnalis*) provide further evidence that vertebrate steroids do not have a hormonal role in the reproduction of mollusks. Front Endocrinol. (2022) 13:981564. doi: 10.3389/fendo.2022.981564 PMC9493083 36157463

[45] Schwarz TI Katsiadaki I Maskrey BH Scott AP. Uptake and metabolism of water-borne progesterone by the mussel, *Mytilus* spp. (Mollusca). J Steroid Biochem Mol Biol. (2018) 178:13–21. doi: 10.1016/j.jsbmb.2017.10.016 29107179

[46] Katsiadaki I Schwarz TI Cousins ARO Scott AP. The uptake of ethinyl-estradiol and cortisol from water by mussels (*Mytilus* spp.). Front Endocrinol. (2021) 12:794623. doi: 10.3389/fendo.2021.794623 PMC8714933 34975764

[47] Gooding MP LeBlanc GA. Biotransformation and disposition of testosterone in the eastern mud snail *Ilyanassa obsoleta* . Gen Comp Endocrinol. (2001) 122:172–80. doi: 10.1006/gcen.2001.7630 11316422

[48] Labadie P Peck M Minier C Hill EM. Identification of the steroid fatty acid ester conjugates formed in *vivo* in *Mytilus edulis* as a result of exposure to estrogens. Steroids. (2007) 72:41–9. doi: 10.1016/j.steroids.2006.10.003 17126373

[49] Puinean A-M Labadie P Hill EM Osada M Kishida M Nakao R. Laboratory exposure to 17b-estradiol fails to induce vitellogenin and estrogen receptor gene expression in the marine invertebrate *Mytilus edulis* . Aquat Toxicol. (2006) 79:376–83. doi: 10.1016/j.aquatox.2006.07.006 16930737

[50] Janer G Mesia-Vela S Porte C Kauffman FC. Esterification of vertebrate-type steroids in the Eastern oyster (*Crassostrea virginica*). Steroids. (2004) 69:129–36. doi: 10.1016/j.steroids.2003.12.002 15013691

[51] Dimastrogiovanni G Fernandes D Bonastre M Porte C. Progesterone is actively metabolized to 5alpha-pregnane-3,20-dione and 3beta-hydroxy-5alpha-pregnan-20-one by the marine mussel *Mytilus galloprovincialis* . Aquat Toxicol. (2015) 165:93–100. doi: 10.1016/j.aquatox.2015.05.018 26026673

[52] Levin ER Hammes SR. Nuclear receptors outside the nucleus: extranuclear signalling by steroid receptors. Nat Rev Mol Cell Biol. (2016) 17:783–97. doi: 10.1038/nrm.2016.122 PMC5649368 27729652

[53] Levin ER. Membrane estrogen receptors signal to determine transcription factor function. Steroids. (2018) 132:1–4. doi: 10.1016/j.steroids.2017.10.014 29155215

[54] Fodor I Koene JM Pirger Z. Neuronal transcriptome analysis of a widely recognised molluscan model organism highlights the absence of key proteins involved in the *de novo* synthesis and receptor-mediation of sex steroids in vertebrates. Malacologia. (2021) 64:69–77. doi: 10.4002/040.064.0103

[55] Hochberg GKA Liu Y Marklund EG Metzger BPH Laganowsky A Thornton JW. A hydrophobic ratchet entrenches molecular complexes. Nature. (2020) 588:503–8. doi: 10.1038/s41586-020-3021-2 PMC8168016 33299178

[56] Gu W Thitiphuree T Otoki Y Marquez EC Kitano T Itoh N. Expression and functional analyses for estrogen receptor and estrogen related receptor of Yesso scallop, *Patinopecten yessoensis* . J Steroid Biochem Mol Biol. (2023) 231:106302. doi: 10.1016/j.jsbmb.2023.106302 36990165

[57] Keay J Bridgham JT Thornton JW. The *Octopus vulgaris* estrogen receptor is a constitutive transcriptional activator: evolutionary and functional implications. Endocrinology. (2006) 147:3861–9. doi: 10.1210/en.2006-0363 16690796

[58] Thornton JW Need E Crews D. Resurrecting the ancestral steroid receptor: ancient origin of estrogen signaling. Science. (2003) 301:1714–7. doi: 10.1126/science.1086185 14500980

[59] Bannister R Beresford N Granger DW Pounds NA Rand-Weaver M White R. No substantial changes in estrogen receptor and estrogen-related receptor orthologue gene transcription in Marisa cornuarietis exposed to estrogenic chemicals. Aquat Toxicol. (2013) 140-141:19–26. doi: 10.1016/j.aquatox.2013.05.002 23747549 PMC3778743

[60] Markov GV Laudet V. Origin and evolution of the ligand-binding ability of nuclear receptors. Mol Cell Endocrinol. (2011) 334:21–30. doi: 10.1016/j.mce.2010.10.017 21055443

[61] Thomas P. Membrane progesterone receptors (mPRs, PAQRs): review of structural and signaling characteristics. Cells. (2022) 11:1785. doi: 10.3390/cells11111785 35681480 PMC9179843

[62] Filardo EJ Thomas P. Minireview: G protein-coupled estrogen receptor-1, GPER-1: its mechanism of action and role in female reproductive cancer, renal and vascular physiology. Endocrinology. (2012) 153:2953–62. doi: 10.1210/en.2012-1061 PMC3380306 22495674

[63] Barton M Filardo EJ Lolait SJ Thomas P Maggiolini M Prossnitz ER. Twenty years of the G protein-coupled estrogen receptor GPER: Historical and personal perspectives. J Steroid Biochem Mol Biol. (2018) 176:4–15. doi: 10.1016/j.jsbmb.2017.03.021 28347854 PMC5716468

[64] Zhang Y Guerra LAY Egertová M Zampronio CG Jones AM Elphick MR. Molecular and functional characterization of somatostatin-type signalling in a deuterostome invertebrate. Open Biol. (2020) 10:200172. doi: 10.1098/rsob.200172 32898470 PMC7536072

[65] Kreienkamp HJ Larusson HJ Witte I Roeder T Birgül N Hönck HH. Functional annotation of two orphan G-protein-coupled receptors, Drostar1 and -2, from *Drosophila melanogaster* and their ligands by reverse pharmacology. J Biol Chem. (2002) 277:39937–43. doi: 10.1074/jbc.M206931200 12167655

[66] Baker ME. Xenobiotics and the evolution of multicellular animals: Emergence and diversification of ligand-activated transcription factors'. Integr Comp Biol. (2005) 45:172–8. doi: 10.1093/icb/45.1.172 21676759

[67] Thomas P Pang Y Dong J Groenen P Kelder J de Vlieg J. Steroid and G protein binding characteristics of the seatrout and human progestin membrane receptor alpha subtypes and their evolutionary origins. Endocrinology. (2007) 148:705–18. doi: 10.1210/en.2006-0974 17082257

[68] Fujimoto K Totani Y Nakai J Chikamoto N Namiki K Hatakeyama D. Identification of putative molecules for adiponectin and adiponectin receptor and their roles in learning and memory in *Lymnaea stagnalis* . Biol (Basel). (2023) 12:375. doi: 10.3390/biology12030375 PMC10045044 36979067

[69] Thomas P Converse A Berg HA. ZIP9, a novel membrane androgen receptor and zinc transporter protein. Gen Comp Endocrinol. (2018) 257:130–6. doi: 10.1016/j.ygcen.2017.04.016 28479083

[70] Taubenheim J Kortmann C Fraune S. Function and evolution of nuclear receptors in environmental-dependent postembryonic development. Front Cell Dev Biol. (2021) 9:653792. doi: 10.3389/fcell.2021.653792 34178983 PMC8222990

[71] Zhao XF. G protein-coupled receptors function as cell membrane receptors for the steroid hormone 20-hydroxyecdysone. Cell Commun Signal. (2020) 18:146. doi: 10.1186/s12964-020-00620-y 32907599 PMC7488307

[72] Lafont R Serova M Didry-Barca B Raynal S Guibout L Dinan L. 20-Hydroxyecdysone activates the protective arm of the RAAS *via* the MAS receptor. J Mol Endocrinol. (2022) 68:77–87. doi: 10.1530/JME-21-0033 34825653

