# Supplementary material for: Lack of membrane sex steroid receptors for mediating rapid endocrine responses in molluscan nervous systems
Source: Front Endocrinol (Lausanne). 2024 Aug 12;15:1458422. doi: 10.3389/fendo.2024.1458422 (PMC11345136; doi:10.3389/fendo.2024.1458422)
Supplement: Supplementary file 1 [file DataSheet_1.docx]

**Supplementary information**

**Lack of membrane sex steroid receptors for mediating rapid endocrine responses in molluscan nervous systems**

^1^István Fodor^+,*^, ^2^Shin Matsubara^+^, ^2^Tomohiro Osugi, ^2^Akira Shiraishi, ^2^Tsuyoshi Kawada, ^2^Honoo Satake^+^, ^1^Zsolt Pirger^+^

^1^Ecophysiological and Environmental Toxicological Research Group, HUN-REN Balaton Limnological Research Institute, Tihany, 8237, Hungary

^2^Bioorganic Research Institute, Suntory Foundation for Life Sciences, 8-1-1 Seikadai, Seika-cho, Soraku-gun, Kyoto, 619-0284, Japan

*Corresponding author; E-mail: [fodor.istvan@blki.hu](mailto:fodor.istvan@blki.hu) (I Fodor)

^+^Equally contributed authors

**Supplementary Table 1.** Molluscan and vertebrate **q**uery sequences used for homology-searches in the *Lymnaea* transcriptome and genome data

| **Query sequence** | **NCBI identifier and/or reference paper** |
| --- | --- |
| Human GPER1 | NP_001091671.1 |
| Mouse GPER1 | NP_084047.2 |
| Chicken GPER1 | NP_001155877.1 |
| Zebrafish GPER1 | NP_001122195.1 |
|  |  |
| Human mPRα | NP_848509.1 |
| Mouse mPRα | NP_001272778.1 |
| Chicken mPRα | NP_001157123.1 |
| Zebrafish mPRα | AAN78115.1 |
| Human mPRβ | NP_588608.1 |
| Mouse mPRβ | NP_083105.3 |
| Chicken mPRβ | NP_001008462.1 |
| Zebrafish mPRβ | NP_899187.1 |
| Human mPRγ | NP_060175.3 |
| Mouse mPRγ | NP_083024.1 |
| Chicken mPRγ | XP_015147701.2 |
| Zebrafish mPRγ | Q7ZVH1.2 |
| Human mPRδ | NP_079173.2 |
| Mouse mPRδ | NP_001391037.1 |
| Zebrafish mPRδ | XP_056331726.1 |
| Human mPRε | NP_940906.1 |
| Mouse mPRε | NP_940806.2 |
| Chicken mPRε | NP_001393213.1 |
| *Sepiella* mPRβ | AVA07271.1; (1) |
| *Octopus* mPRβ | XP_014774875.1; (1) |
| *Crassostrea* mPRβ | XP_019918893.1; (2) |
| *Biomphalaria* mPRβ | XP_013077721.1; (1) |
| *Lottia* mPRβ | (2) |
| *Sepiella* mPRγ | AVA07272.1; (1) |
| *Octopus* mPRγ | XP_014787281.1; (1) |
| *Crassostrea* mPRγ | (2) |
| *Biomphalaria* mPRγ | XP_013087777.1; (1) |
| *Lottia* mPRγ | (2) |
|  |  |
| Human GPRC6A | NP_683766.2 |
| Mouse GPRC6A | NP_694711.1 |
| Chicken GPRC6A | XP_040522589.1 |
| Zebrafish GPRC6A | AAI63279.1 |
| Human ZIP9 | NP_060845.2 |
| Mouse ZIP9 | NP_080520.2 |
| Chicken ZIP9 | NP_001007934.1 |
| Zebrafish ZIP9 | NP_001013558.1 |
| *Aplysia* ZIP9 | XP_005097024.1 |
| *Biomphalaria* ZIP9 | XP_055878952.1 |

**Supplementary Figure 1.** Nucleotide (cds) and protein sequences of *Lymnaea* homologs to vertebrate membrane sex steroid receptors

>GPER1 candidate_nucleotide (NCBI: #PP943023)

ATGGAGACCACAGTCGGGGCGGTGGCCAATCTCACCAACTCCACTTCGGACATGCCCACCTACCTCAAGGTGCTTCCCTACGCCGTCATCGTCTGCTACCTGCTCATCTGCGCGCTCGGCCTCATCGGGAACGGCCTGGTCATCTACGTCGTCCTCATGTTCGCCAAGATGAAAACGGTGACCAACATGTACATCCTGAACTTGGCGCTGTCCGACGTCCTGTTCCTGACCATGCTCCCCATCCTGGCCACCACGTCCATCGTCCAGCACTGGATCTTCGGCTTCGCCATGTGCAAGATCTACTTCGTCGTCTACAGCATCAACCTGTTTGGTGGGGCCTTCAACCTGTGTCTGATGAGCGCGGACAGGTACATGGCGGTCTGCCACCCCATTCGTTCCCTGAGGTACCGTACTCCAAGAATCGCTTTGTTCCTCTGCCTGTGTATCTGGTCGCTGGCGTTTCTCGTCATGATGCCTACCATTCTCTACAGCAAGACCATGGACCACCGTATATTTAAAGGAAAATACTCATGCAATATTAAATGGCCCGAAAATCAGGCCATCTCGTCCGACAAAGCCTTTATTTGGTATTCCTTCATCCTGGGCTTCTCTATCCCCGTCTCCCTCATCTCCGTGTTCTACGTGCTGGTCATACTGAGGCTGCGTCACGTGGGGCCCGCAAAGAAGTCGAAAGAAAAGCGGAAGTCCCACCGCCGCGTCACTCGCCTCGTGCTGACCGTGATCGCTATATACATTGTCTGCTGGCTGCCTTACTGGGTCTTCCAAGTTGTTCTTGTGTTTCAAAACTATAAGATTAGCTCGCCCTCGAAGGTGTTGCTCTTCAACTTCTTCACCATCCTCTCCTACGCCAACAGCATGCTCAACCCATTCCTTTACGCTTTCCTCAGCGACAACTTCCGGAAGAGTTTTCTCAAAGCCTTCAAGTGTGTGTCCAGCATCGAGGCCAACAGGTCTGTGTGTAATGAGAATAGTGTGTTTCCCAGGACCAGTCAAACCTACACGCGCAGTGGCGTCACGTCTGAGGAGAGGATGGAGCTGTCATCCATGGAGGCCACCTCGATGTCTCCCCCTCCTTGTGATCAAAAGTCGATCAAGTTGCTGAAGGATGAGCATGGCTTTCTGAAACCACCAGTCCATCTGTGA

>GPER1 candidate_protein

METTVGAVANLTNSTSDMPTYLKVLPYAVIVCYLLICALGLIGNGLVIYVVLMFAKMKTVTNMYILNLALSDVLFLTMLPILATTSIVQHWIFGFAMCKIYFVVYSINLFGGAFNLCLMSADRYMAVCHPIRSLRYRTPRIALFLCLCIWSLAFLVMMPTILYSKTMDHRIFKGKYSCNIKWPENQAISSDKAFIWYSFILGFSIPVSLISVFYVLVILRLRHVGPAKKSKEKRKSHRRVTRLVLTVIAIYIVCWLPYWVFQVVLVFQNYKISSPSKVLLFNFFTILSYANSMLNPFLYAFLSDNFRKSFLKAFKCVSSIEANRSVCNENSVFPRTSQTYTRSGVTSEERMELSSMEATSMSPPPCDQKSIKLLKDEHGFLKPPVHL*

>mPR candidate_1_nucleotide (NCBI: #PP943022)

ATGTTGTTACTGCCAGCAACATTGAGTAGGGAAGAAATCCCAATACTTTTTCATGAACCCCATGTTATGAAAGGGTTTAGGCCACTGCACTACCCATGGATGTCTTATTTTTTAAGTTTGTTTCAGTGGCATAATGAACTCTTGAACATCTGGACACACCTACTGGCATTAATCATGGTGTTGGTGAGGGCTAGTATGTGGTGGACAGAGTTTGACCTGCTCCGAGACCCTTACATGTGGCCACTGTCAGTGGGGATCATCACTATGATTATTCTCTATGTCTGCAGCTCTGGTGCTCATTGTTTTCAGAACAGGTCAGAATTGGTCCACTACACCTGTTTCATGTTTGACTATGCTGGTATAGGACTCTATGGTTTTGGAAGTACCATGCTACACTACTGGTATTGTCTCCATGAAAGTTTCATGGGCTCTTTATCCCACCAGCTCGCTATACCAGTTGGAGCAATTTTAGCAGTCTTGGTCTGTATCTGCTGCTCTATTTCCAAAACTAAGTACAAGCGCCCTTACCCTTTCACTCGTCGTATCTGGCAGATGTCCTCAGTGGCCTCTATTTACATTTGGCTTATATTCCCAATTTGGTACAGAATTTGGTTGTATGTCCACACTGGAGAGTGGGATCCCAGTTTTAAGCACCATATTCGTCAGATGTGTTGGTTCACTTTGGGAGGATTTTTCTTTGGTTCAGACATTCCTCAAAGGTTCTTTCCGGGCACTTTTGATATTATCGGTCACAGCCATCAGCTGTTCCACATATGTATATTCATGACCACCTATGAACAGCTGAGTGCCCTGTACTTGGAGTTGACCGGCATCAGTGTCATCATCCACAACATGGAGTCCCCAACTCTCTTCAACACCTGGGGAGTTCTCATCCTAGTTGTTGTTTGCAATTCCATCGTGGTTTATTTATTTCACATCTCCGTAGAGAGGAAGCTTGAAGATGAGAGGAAGAAGGAGGATAAGGAAGAGGAGGATAAGGGAGAGGGCGTGTTAAAGGAAAAAGCTGAACTTAAGAGTAATTCTCCAAACAGGAAAAGTGCCAATGGTGATGGCTGTGATGTTCATCACAGAAATGGGGTCATCCATTAG

>mPR candidate_1_protein

MLLLPATLSREEIPILFHEPHVMKGFRPLHYPWMSYFLSLFQWHNELLNIWTHLLALIMVLVRASMWWTEFDLLRDPYMWPLSVGIITMIILYVCSSGAHCFQNRSELVHYTCFMFDYAGIGLYGFGSTMLHYWYCLHESFMGSLSHQLAIPVGAILAVLVCICCSISKTKYKRPYPFTRRIWQMSSVASIYIWLIFPIWYRIWLYVHTGEWDPSFKHHIRQMCWFTLGGFFFGSDIPQRFFPGTFDIIGHSHQLFHICIFMTTYEQLSALYLELTGISVIIHNMESPTLFNTWGVLILVVVCNSIVVYLFHISVERKLEDERKKEDKEEEDKGEGVLKEKAELKSNSPNRKSANGDGCDVHHRNGVIH*

>mPR candidate_2_nucleotide

ATGGCATCTGGTTACCCAGCTAACTCCTTTGGGAGGCTCGGGGTGGCTTGCTCACAGACCATCACAGAAGAGCCTTTCAGCCCAGAAACATCCAGTCTGGCTGGCAACTTTGACACAACTGAAAGTATAAACTCTTTTCATCTGCCCGGGGAGTTGGGGGATGGAAACACTGTGGGGAAGTCTAAATCAAAATCTGGCTATGGCTATAACCGTGCGGAATTAGGTTCTTTACTGGCCGCTGAGGATGATAAACTACCCCCTGAACTTGATGAGGATCTGGATGGAGTAGACACCTCCTTGTTAAATGTCCCCAGTAAATGTGCTCAACAAGCAGAAGAGTTTGTAAAGAAGGTGTGGGCTGCTGGCTGGAATGTCGCCCACCACCACACCCTGCCTGACTGGCTCAAGGACAATGACTTTTTACTCAGAGGCCATCGTGTTCCAACAAACTCATTCATAGCATGTTTCAAGTCCATTTTTAGAATTCACACTGAAACTGGAAACATATGGACGCATTTGCTAGGCATGATAGCTTTCCTTGGCATTGCTGCCTACTTCTTAACTCGACCTTCCATTGAGATCCAGTGGCAAGAAAAGGCAGTTTTCTCAGCTTTTTTTATGGGGGCCATTTTATGTCTGGGCTTTTCGTGGGTGTTCCACACCGTCTACTGCCACAGTGAACGTGTTGGTAGATTTTTTAACAAACTTGATTATTGTGGTATAGCATTGCTCACCATAGGTTCCTTTGTTCCCTGGCTGTATTATAGTTTCTATTGTCGCCTGGAGCCCAAAGTAACTTACTTAGCTTTGATATTTTTCCTGGGAACTATTGCCATCGTGGTCTCCATGTGGGACAAGTTTGCTCAACCACAGTACAGGCCTTTGAGGGCAGGAGTGTTTGTTGCCCTGGGTCTGAGTGGAGTTATTCCCGCTATGCATTATGTGATCACAGATGGTTTTTGGCATGCCATTAACTATGCAGCCCTGGGCTGGCTGGTCCTCATGGCTTTACTATATATTGTTGGAGCTGTCATCTATGCTGTTAGAATTCCAGAGAGGATCTTTCCCGGCAAGTTCGATATTTGGTTCCAAAGTCACCAGATTTTCCATGTCTTTGTGCTGGCTGCAGCTTTTGTGCACTACCATGGGATATCGGAGATCGCCAACTACAGACTTACCCTGGGGGACTGTATAGCAAGGGGAGAGGTCGAGTAG

>mPR candidate_2_protein

MASGYPANSFGRLGVACSQTITEEPFSPETSSLAGNFDTTESINSFHLPGELGDGNTVGKSKSKSGYGYNRAELGSLLAAEDDKLPPELDEDLDGVDTSLLNVPSKCAQQAEEFVKKVWAAGWNVAHHHTLPDWLKDNDFLLRGHRVPTNSFIACFKSIFRIHTETGNIWTHLLGMIAFLGIAAYFLTRPSIEIQWQEKAVFSAFFMGAILCLGFSWVFHTVYCHSERVGRFFNKLDYCGIALLTIGSFVPWLYYSFYCRLEPKVTYLALIFFLGTIAIVVSMWDKFAQPQYRPLRAGVFVALGLSGVIPAMHYVITDGFWHAINYAALGWLVLMALLYIVGAVIYAVRIPERIFPGKFDIWFQSHQIFHVFVLAAAFVHYHGISEIANYRLTLGDCIARGEVE*

>GPRC6A candidate_nucleotide (NCBI: #PP943024)

ATGGCAACGCGCGTCAGAGGCTACCGTCGCACGTTGCAGGCCCTTGTCGCCTTCAGAGTGGTGGCCATCATGGCGGACTTTGCCTTCCAGCCCCTCCCCGAGGTCACGGGCAACGTGACGATCCCGACGCTGTCCGCGACCATCAACGGGGACATCATCATTGGCGGGTTGTTCCCCATTCACAACAAGGGCGGCAAGGGCTGCGGGACGATCAACGCCGACCGGGGCATAGAACGCCTGGAGGCCTTCATGTTCACCATCGACGAGATCAACAACAGCACCGTGCTGCTTCCCGGAATCACCATTGGGGTCGCCGCCTTCGACACTTGCGCCCTAGCGCCCTACGCCCTGGAGCAGTCCCTCGAGTTCATCCGGGCGTCGATAACGTCGTTGGACCCTGCGGAATTCTACTGCAGCGACGGATCCAAGGCGAAGGCCGTCAGCACGGCCACCGCCGTTGCCGGCGTCGTCGGCGGATCGTACAGCACCGTCTCCATCCAAGTGGCCAACCTGCTTCGCCTTTTCAAGATCCCGCAGATCAGCTACGCCTCCACGTCAGCGTCTCTCAGCGATAAAACCCGGTACGATTACTTCGTCCGAACCGTCCCGCCCGATACCCTGCAGGCGAAAGCTCTCGCGGATATTGTTGCCGAATTCAATTGGACGTACGTATCAGTTGTCCACTCCGAGGGCGAGTACGGCGAGCCCGGAATCGATTCGTTCAAAGTAGAGGCCCGGGCTAAAAATATCTGCATCGCGGCCGACATCGAGATCGCCACCACGCCCACGAACGCCACCTACGAGCAGGTTCTCAGGGACCTCTTAGACAAGCCCGAGGCAAAAGTGGTGATCGTGTTCGTGCGGACCGAGGACGCGGAGGGCCTGCTGAACGCGGCCACCAGACAAAACCTGACGGGGAAGTTCGTCTGGATTGCCAGCGACGCCTGGGGAAACCGGATAGCGCCGGTGAAGAACAACCCGCTGGCCGCTCAAGGGGCGATAACTTTAGAACTTCAATCATCGCCGATCCGTAAATTCGAGGAGTATTTCTTGAACTTGAACCCGCGGACCAACAAGCGCAACCCGTGGTTCATCGAGTACTGGGAGGAGGAGCACAAGTGCGCGTGGAACGGCGTCGCCCAGGGCGCCGCAGGCTCACGGGTGCACTCGCCCGTGAACCCCTGCAAGGGGGACGAGCGCGTGAACCGGAAGCTGACGTCGCAGGAGAGCAAGACGCAGTTCATCTACGACGCCGTGTACGCGTTCGCCCACGCCCTGGACAAGATGCACCGTGACCTGTGCCCCGGGATGCCCGGAGTCTGTCAGAAGATGGAGAAGATCGACGGGGAGAAGCTACTGCGGGAGTATCTGCTCAAAGTCTCGTTCGATAATGGCCACGGTGCTACGGTACAGTTTGACAAGAAAGGAGACGCCCCTGGCAGGTACACGGTCATGAACTTCCAGCGCAACCGACGGACCAGGGAGTACGAGTACCACGTGGTCGGCACGTGGAACAACGGCCTGACGGTCAACCACTCGGAGATCATCTGGGCCGGCGAGACCAAGGACATCCCGCGGTCGCGCTGCAGCGACCCGTGCCAGGAGGGCCAGATCAAGAACATGCAGAAGGGCGAGCAGTGCTGCTGGATCTGCACGCGGTGCAACCCGTGGGAGTACATCAAGAACGAGAAGACGTGCGAGCCGTGCGACGCTGGCTTCTGGCCCTACCCCAACAAGACCGGCTGCTTCGCCCTCGAGGTCCAGTACATGACATGGGGCAGCATCCACGCGGTAATCCCCATGGTCCTAGCCACGATCGGGATCTTGTGCACGGGCTTCGTGATCTGCCTCTTCGTGATGTTCAACAGCACCCCGGTCGTCATGGCCTCGGGCCGGGAACTCAGCTACATGCTGCTCGGGGGCTGTGTCTTCTGCTACTTCATGACGTTCATGATCGTCGCGCCGCCCTCCACGCTCATGTGCGCCATTCAGAGATTCGGGGTCGGGTTCGGGTTCAGCATCGTGTACTCCTCCCTGCTCATAAAGACCAATAGGATATCTAGGATATTCGAGAGCGCCCGGCGCTCCGCGAAACGCCCGCCGTTCATCAGTCCAAAGTCGCAAATAGTAATGACGTGTATACTCATACTAATCCAGGTACTCTTCACGTTCGTCTGGCTGCTCCTGGAGCCACCGGGCACGAGGCTCATGTACCCAGACTCGCGTGAGCCGGTCGTCATCCGCAAGTGCAAGAGCGACGACATCTCGTTCCTCATCAGCCTGGTGTACAACATGCTCCTCATCATCATCTGCACGGTGTACGCGGTCAAGACGAGGAAAATTCCAGAAAACTTCAACGAATCCAAGTTTATAGGCTTTTCCATGTACACCACCTGTATCATATGGCTCGCCTTTGTGCCTATTTATTTCGGGACTCTCCATTCGTTTAGAATCCAGATCACCACTCTGTGTGTGTCCATCAGTCTGAGTGCTAGTGTGGCTCTACTGTGCCTCTTCCTGCCCAAAGTCTACATCATCGTCTTCCAGCCTCAGAAGAACGTGCGCAAGTTGACCATGAACTCGGCCAGTTACAAGATGGCGCCCACGGCCTCCTCGGCGACCGGGAACAACCACAGCGCGCCGAGTTCTGAGCACTTACGTCTGAACGTCCAGCCCAGAATCAACATGCCAGGGACGACAACTGAGACAGAAACGGACGACAGGGATTCCCTAGCGTCACTCTGA

>GPRC6A candidate_protein

MATRVRGYRRTLQALVAFRVVAIMADFAFQPLPEVTGNVTIPTLSATINGDIIIGGLFPIHNKGGKGCGTINADRGIERLEAFMFTIDEINNSTVLLPGITIGVAAFDTCALAPYALEQSLEFIRASITSLDPAEFYCSDGSKAKAVSTATAVAGVVGGSYSTVSIQVANLLRLFKIPQISYASTSASLSDKTRYDYFVRTVPPDTLQAKALADIVAEFNWTYVSVVHSEGEYGEPGIDSFKVEARAKNICIAADIEIATTPTNATYEQVLRDLLDKPEAKVVIVFVRTEDAEGLLNAATRQNLTGKFVWIASDAWGNRIAPVKNNPLAAQGAITLELQSSPIRKFEEYFLNLNPRTNKRNPWFIEYWEEEHKCAWNGVAQGAAGSRVHSPVNPCKGDERVNRKLTSQESKTQFIYDAVYAFAHALDKMHRDLCPGMPGVCQKMEKIDGEKLLREYLLKVSFDNGHGATVQFDKKGDAPGRYTVMNFQRNRRTREYEYHVVGTWNNGLTVNHSEIIWAGETKDIPRSRCSDPCQEGQIKNMQKGEQCCWICTRCNPWEYIKNEKTCEPCDAGFWPYPNKTGCFALEVQYMTWGSIHAVIPMVLATIGILCTGFVICLFVMFNSTPVVMASGRELSYMLLGGCVFCYFMTFMIVAPPSTLMCAIQRFGVGFGFSIVYSSLLIKTNRISRIFESARRSAKRPPFISPKSQIVMTCILILIQVLFTFVWLLLEPPGTRLMYPDSREPVVIRKCKSDDISFLISLVYNMLLIIICTVYAVKTRKIPENFNESKFIGFSMYTTCIIWLAFVPIYFGTLHSFRIQITTLCVSISLSASVALLCLFLPKVYIIVFQPQKNVRKLTMNSASYKMAPTASSATGNNHSAPSSEHLRLNVQPRINMPGTTTETETDDRDSLASL*

>ZIP9 candidate_nucleotide (NCBI: #PP943021)

ATGGATGATATCTTGACTCTCCTTTCTCTGTCCATAGCGATGCTTGTTGGCTGTTACCTTGCAGGAGTCATCCCACTGACTATTTCACTTTCAGAGGAAAAACTTAAATTAGTGACAGTTCTTGGTGCTGGATTGTTGGTTGGCACTGCCCTTGCAGTTATTATACCAGAGGGTGTTCATGCAATGTACTCAAGTTATGAAGCCCACGAGCATTCACATGATCATGCAAGAGAAGCTGAGCCCATTCAAGCATCTGATGCTGCAAAAAATGATAAACAAGGGAGCTCTGCATTAAAAGATCCAGCCTCACAGCTTGAGCACCATCATGAGCACAGCAAACCAGACGTGCATTCTATTATAGGCGTTACCCTAGTTATTGGGTTTATTTTCATGTTGTTGGTGGATCAGATAGGGGGGAGCATGCATTCCCATGCGATTTCTTCAGACCCAGAGACGGCGAGTCATGGACAGAGTAGAAATAAAATTACAGCCACACTTGGACTTGTTGTCCACGCTGCAGCCGATGGTATAGCCCTTGGAGCCGCCATATCATTATCTGAAACTCACATCACCATGATTGTTTTTATAGCAATCATGTTGCATAAGGCTCCTGCAGCTTTTGGTTTGGTTTCCTTTTTAATGCACGAGGGATTGGATCGAACAAGAATAAGGAAACATTTAGCAGTATTTTCTGCTGCTGCACCACTTTTAACCATTATAACTTACCTTGGCCTAAGTCAGCAAAGTAAAGAAACATTGTCAGATATGCAAACAACAGGGATTGCAATGTTGTTTAGTGCTGGGACATTTTTATATGTGGCCACTGTTCATGTCCTTCCAGAAATTTCTGTAAGCCAGACACAGCATAAGGCAGCAGATGGAACTGTAATTATTCGAGAGCAGAAAGGTTTCAAGAAATTGGAGCTTGCAGCCTTAGTGTTGGGTGCACTTTTACCTGTTTTTTTAGCTGTGGGTCATAAGCACTGA

>ZIP9 candidate_protein

MDDILTLLSLSIAMLVGCYLAGVIPLTISLSEEKLKLVTVLGAGLLVGTALAVIIPEGVHAMYSSYEAHEHSHDHAREAEPIQASDAAKNDKQGSSALKDPASQLEHHHEHSKPDVHSIIGVTLVIGFIFMLLVDQIGGSMHSHAISSDPETASHGQSRNKITATLGLVVHAAADGIALGAAISLSETHITMIVFIAIMLHKAPAAFGLVSFLMHEGLDRTRIRKHLAVFSAAAPLLTIITYLGLSQQSKETLSDMQTTGIAMLFSAGTFLYVATVHVLPEISVSQTQHKAADGTVIIREQKGFKKLELAALVLGALLPVFLAVGHKH*

**Supplementary Figure 2.** Conserved domain analysis of the receptor candidates

1) human GPER1


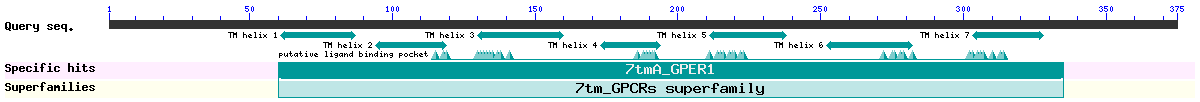


*Lymnaea* GPER1 candidate


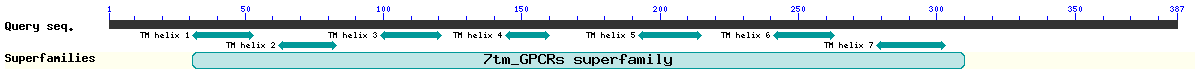


2) human mPRα


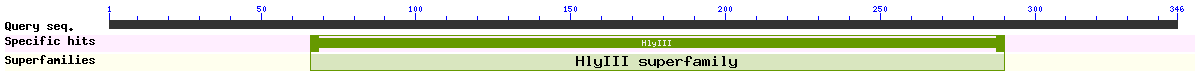


*Lymnaea* mPR candidate 1


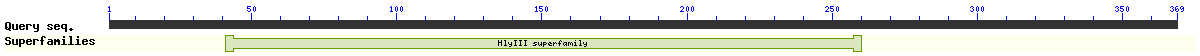


*Lymnaea* mPR candidate 2


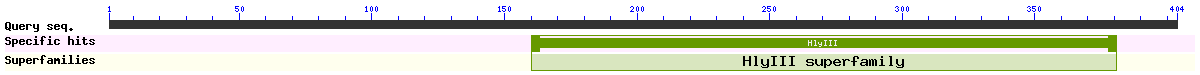


3) human GPRC6A


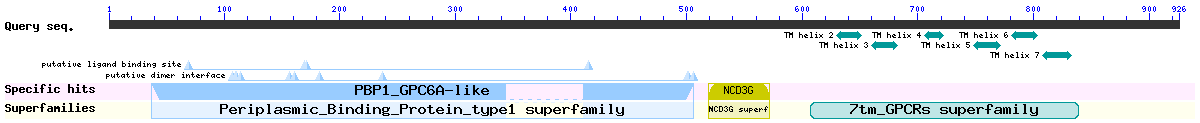


*Lymnaea* GPRC6A candidate
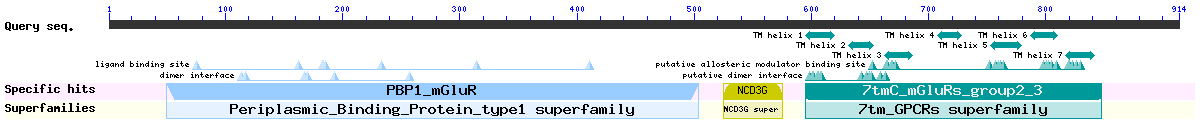


4) human ZIP9


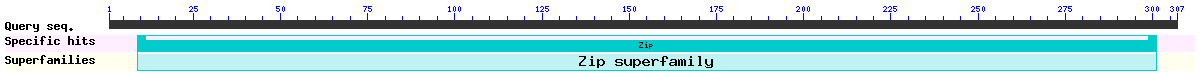


*Lymnaea* ZIP9 candidate


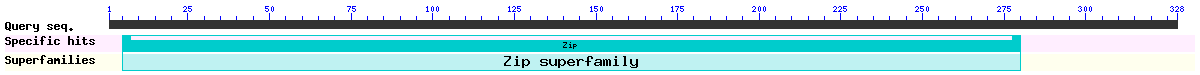


**Supplementary Figure 3.** Phylogenetic analysis of *Lymnaea* GPER1 candidate. For the analysis, relevant vertebrate GPER1 sequences and, based on the homology-searches, invertebrate allatostatin-C receptor (ASTC-R) and vertebrate somatostatin receptor sequences were used. The alignment was made with MUSCLE in the Molecular Evolutionary Genetics Analysis v7 software (3). The maximum-likelihood tree was made with the LG general amino acid replacement matrix and four discrete gamma models. Bootstrapping support for the tree was conducted with 1000 bootstrap replicates, the bootstrap values (%) are indicated at each branch point. All positions containing gaps and missing data were eliminated. There was a total of 240 positions in the final dataset. The *Lymnaea* sequence is marked by a red arrow. Based on the analysis, we supposed that *Lymnaea* GPER1 candidate was actually a member of the somatostatin/ASTC-type GPCRs family, but we did not exclude it from further experiments.

**
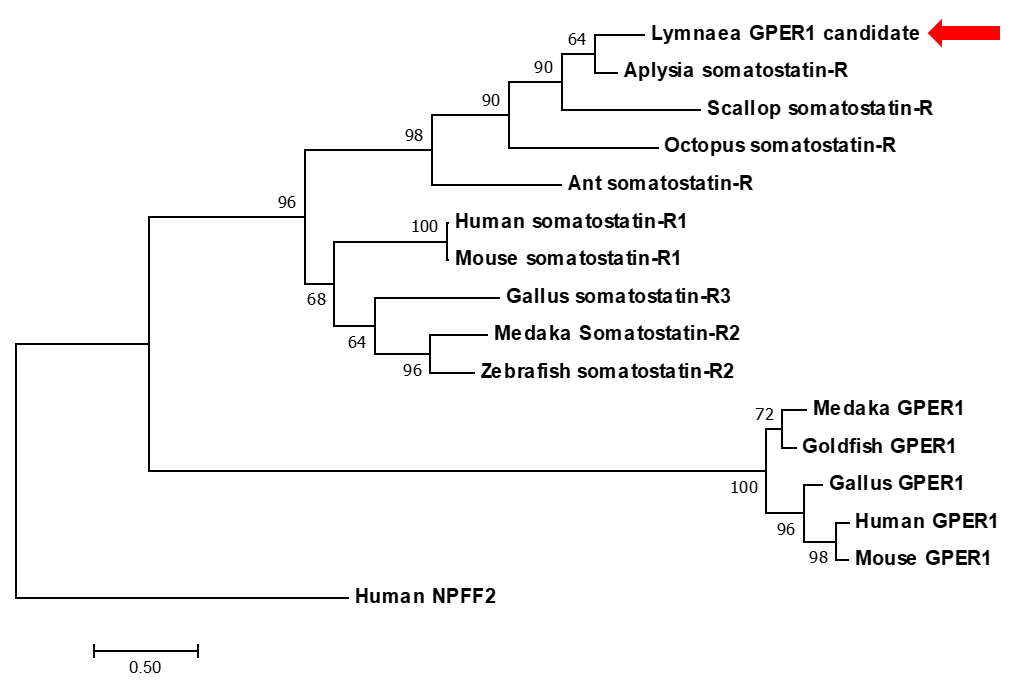
**

>Aplysia_somatostatin-R_XP_005095139.1

MAKPIPTMDSPYTAYSFSPNETLETLLNVLTTLPTCNISDNVTESNLNDTGMPERSGYAKVLPYVMIVCYILICILGLAGNGLVIYVVLMFAKMKTVTNMYILNLALSDILFIVILPMMATTTLMEHWIFGFAMCKIYFVLYSINLFGGAFNLCVMSADRYLAVCHPIRSLKYRTPRIALFLCLCVWSLSFLVMLPIILYSRTEKHRRFPGKESCSIFWPKDQLIPPDKAFIWYSFILGFAIPVSLISVFYVLVILRLRHVGPAKKSKEKRKSHRRVTRLVLTVITIYVVCWLPYWCFQVHIVFNDVKITSDAMILVFNGFTVLSFANSMLNPFLYAFLSDNFRKSFVKAFKCLSNVEMNKSVCNENSVFPRTSQTYTRSGVMGEERMELSAVDTNPTASPNVAEQGSIKSPQDEHGFLKPPVQL

>Limpet_somatostatin-R_V4B6H9

MIENVTDLDCDFCNSTTASNITGSDDQISDILKIIPIVLVLCYVIVLFVGWIGNGLVIYVVARFSKMKTVTNMYVLNLAVSDVMFIFSLPFLTTTTLLGYWVFDFVMCKIYFVLYSINVFTSVFTLTVMSADRYLAVCHPVRSVKYRTPRIALFVCLCIWTISFLVMLPIILYSTTVPHRLYPDKHTCTIKWPNGQLIPAGQAFIWYTFLLGFAIPVSLTSVFYFLVVLRLKTVGPVKKSKEKKKSHRRVTRMVLSVISAYVVCWLPYWLFQVYIIVREPPVLVPWKVILFNSFTVLTYTNSMLNPFLYAFLSENFRKSFMKAFKCVSPTDANKSLCHENSMFPKNNQNFGRSVVTTIEDRLEMSTFEPGQNNIDATPMLPKENHLTTTTLPDEQCLLKPVAL

>Scallop_somatostatin-R_A0A210Q9S3

MALNNTTDSMDCDYLDNGTGVTGDMNVAVSTYVVVVSYACIFLCGIAGNGLVIYVVLRYAKMKTVTNLYILNLAVSDFLYILHLPLISTTTYLKYWMFGNAVCKINFVLYSISFFAGVFTLTSLGGDRYIAVCHAINSQEYRKPKYATLIIIGIWSLSFFVMLPTILYSKTVPNHNFPGKLTCTIEWPSGQLIPTEKAYTWYTFLLGFGIPVSLISVFYLLVILRLRTVGPVNKSKETRKAHKKVTRMVLAIISIYIVCWLPYWCFQVNLTLRPKDKSLEDWEILMFNIFTVLTSSNSMLNPILYAFLSDNFRRSFLKSFQCTNLMDANKSLYGESTLPTKHRRSHDYAFGKGKDMEKIELKEKEKEKKTTENNEYQTQTNGNVTEKCRLLQIHKFVKHTEEKNDNDNSLDVEEPDDVGDHSDTAALYADKQIQTREIV

>Crassostrea_somatostatin-R_XP_052680579.1

MNTTVYNHSHDLDNVTNVDGRKPFLLDNSSHDFHFQFPTQVDIINVICAVIAVLGGIGNIVTIVIISYQSKLHTPTFVVIKCLAVSDFFGLITVSFQYFTNVWTFLRHQNASTYANLFEIIVNTVYLNSPSHVLLLCAVRYLLVVHPLDSRRYLTVTVVSLGSLTSWILSFVFAVIYVSVYSAFGVKDQNIGLALEMASTIIVILYLVLSVIIIISMHLKKMAAIKKSATKGQVHKKMHFIAFVMLFCLVLCRLPDIVVHFFLFVKKTELFTIHLRNCYFVLNYFTYSYNPYMLFVFSCLKSCRKK

>Octopus_somatostatin-R_A0A7E6FMZ4

MEPLFNETVHVTEQSLFGENFNDTYADMNNSLSNATEHSIRRVFMEIVNITVMISNSLICITGLFGNCLVIYVVTMFSKMKTVTNTYILNLAIADVLFLVSIPLLIVTIKNGFWMFGFFMCKFYYLLVSINSFTGVFTLTVMSADRYFAVCHPIKSMSLRTPKVAFIITVCIWLISILIMLPALMYSTTVRNSKNKDSCMMVMPDHLGISPDKIFIWYNFIFSYAVPVPLISVFYISVVHKLRTTGPAKKSSEKKKSQKRVTRLVLTVIGVYIICWLPYWAFQLDIVVFTPPLTEGRILMFQTFNILTYANSMLNPLLYNFLSDNFRKSFTKAFKCTSRFEVNRSLRAENSIYPKGREAYSQTTVIEKQELQTINHCATENKVQEDFVNGETQTEDIDADGPEAVVTELE

>Ant_somatostatin-R_A0A0J7NQD0

MMMNTTMDMMDYVQNSTHNLTHNDIGQNCEAELPIIALVNQVLYSIVCIVGLLGNTLVIYVVLRFSKMQTVTNMYIVNLAIADECFLIGIPFLVTTISLRSWIFGKIMCKAYMTTTSINQFTSSIFLFIMSADRYIAVCHPISSPKMRTPFISRVVSLTAWATSALFMVPVFLYANAMESPEGVISCNIYWPNDRGGQTTFTLYTFILGFAVPLVLILIFYFLVIKKLRTVGPKNKSKEKKRSHRKVTKLVLTVITVYVICWLPYWVTQVALIYTPPKQCQTSISITSFLLAGFLSYSNSAMNPILYAFLSDNFKKSFLKACTCAAGKDVNAALHIENSVFPRRNKANVDRLQSNRMVTSGQSRIELEDEDAERGLLISKTSTTTVTMTSRSNITVSSEPRDQAQRDKDLIKNGAQLTLLTQV

>Human_somatostatin-R1_P30872

MFPNGTASSPSSSPSPSPGSCGEGGGSRGPGAGAADGMEEPGRNASQNGTLSEGQGSAILISFIYSVVCLVGLCGNSMVIYVILRYAKMKTATNIYILNLAIADELLMLSVPFLVTSTLLRHWPFGALLCRLVLSVDAVNMFTSIYCLTVLSVDRYVAVVHPIKAARYRRPTVAKVVNLGVWVLSLLVILPIVVFSRTAANSDGTVACNMLMPEPAQRWLVGFVLYTFLMGFLLPVGAICLCYVLIIAKMRMVALKAGWQQRKRSERKITLMVMMVVMVFVICWMPFYVVQLVNVFAEQDDATVSQLSVILGYANSCANPILYGFLSDNFKRSFQRILCLSWMDNAAEEPVDYYATALKSRAYSVEDFQPENLESGGVFRNGTCTSRITTL

>Mouse_somatostatin-R1_P30873

MFPNGTASSPSSSPSPSPGSCGEGACSRGPGSGAADGMEEPGRNASQNGTLSEGQGSAILISFIYSVVCLVGLCGNSMVIYVILRYAKMKTATNIYILNLAIADELLMLSVPFLVTSTLLRHWPFGALLCRLVLSVDAVNMFTSIYCLTVLSVDRYVAVVHPIKAARYRRPTVAKVVNLGVWVLSLLVILPIVVFSRTAANSDGTVACNMLMPEPAQRWLVGFVLYTFLMGFLLPVGAICLCYVLIIAKMRMVALKAGWQQRKRSERKITLMVMMVVMVFVICWMPFYVVQLVNVFAEQDDATVSQLSVILGYANSCANPILYGFLSDNFKRSFQRILCLSWMDNAAEEPVDYYATALKSRAYSVEDFQPENLESGGVFRNGTCASRISTL

>Medaka_Somatostatin-R2_A0A3P9J5H6

MDVDQWSVLPPSPNLSIPDALFYDADFPANQSAFNTSSSSGHQQDRTSSVVITFISFLVCAVGLCGNTLVIYVILRYAKMKTVTNIYILNLAVADVLCMTSLPFIALQLVLVRWPFGEVLCKIIMTVDSLNQFTSIFSLMVMSIDRYLAVVHPIRSTKWRKPRMAKLINLTVWVVSLLVILPTMIFSGLNKVPVCGIVWPEPQDVYYNTFIFYTFFIGFFFPLVVICLCYLLIIVKVKSSGMRVCSSKRRRSERKVTRMVSIVVAVFVLCWLPFYIFNVTSVTSSITPSSAMKSTFYFVIVLGYANSCANPVLYAFLSDNFRKSFQNVLCLKKGAGLDEMERSDSRADRSRMVNEAALINANLETHNAALLNGELQTSI

>Zebrafish_somatostatin-R2_E7FDL0

MDTWTFMPNSNLSLPDRLVNDSFFPGNESDFGLEFYPPNGTHPGFDHTSSVVITFVYFVVCAVGLCGNALVIYVILRYAKMKTVTNIYILNLAVADVLCMLSLPFIAIQLSLLHWPFGSAICRVVLTVDSMNQFTSIFFLTVMSFDRYLAVVHPIKSTKWRKPRMAKTISLGMWSVALLVNLPIMIYSGVNAKKNEARTCTMLWPEPQNTYYTAFIFYTFFLGFFLPLIVISMCYLLIVIKVKSSGMRVGSTKRKRSERKVTRMVSIVVVVFVLCWLPFYVFNVTSVTGTVPTTPVLKSTFDFVVVLGYANSCANPILYAFLSDNFKKSFQNVLCLKRVGGLDEIDRSDSRQDRTRMVNDIMTETHNAALLNGDLQTTI

>Gallus_somatostatin-R3_Q4ZJF2

MDTSAFSLPTPTVSEEGNASGSWAGFTTPNSSTTTTSPGVVVSGVLIPMVYLIVCVVGLIGNSLVIYVVLRHSVSESVTNVYILNLALADELFMLGLPFLAAQNALSYWPFGSFMCRLVMAVDAINQFTSIFCLTVMSVDRYLAVVHPGKSSKWRTARVAKAVSATVWMLSSIVVLPVVVFSDVPLGMNTCHIQWPEPASVWRAGFIIYTATLGFFGPLLVICLCYLLIVVKVRSSGRRVRALSSKHKLSERRVTRMVVTVVAVFVLCWLPFYVLNIINVVCPLPEEPSLFGVYFLVVVLPYANSCANPIIYGFLSYRFKQGFRRAIFRPSRRVQSQEVPECPPEKSDDGGEEKEISKITQNGNDREERPLSSRAGERNGQKPLPEEPVGCEKSSKLHVSYL

>Medaka_GPER1_H2LJY7

MERQTPALMWIRDNTTEPLNTSDAFNCTDLSEISDKYQSYIAGLFLSCLYTILLFPIGFIGNILILVVNLNHREKMTIPDVYFVNLAVADLILVADSLIEVFNLNEKYYDYAVLCTFMSLFLQVNMYSSIFFLTWMSFDRYVALASSMNSSPLRTMQHAKLSCGLIWMAAILATLLPFTIVQTQHRGELHFCFANVYEIQWLEVTIGFLVPFTIIGLCYSLIGRILMKAQKHHGLWPRRQKALRMIVVVVLVFFICWLPENVFISIQLLQGKADSSQRTAVTLWHDYPLTGHIVNLAAFSNSCLNPIIYSFLGETFRDKLRLFIKQNASWSLVNRFCHHSLDLHLSVRSKVSEV

>Human_GPER1_Q99527

MDVTSQARGVGLEMYPGTAQPAAPNTTSPELNLSHPLLGTALANGTGELSEHQQYVIGLFLSCLYTIFLFPIGFVGNILILVVNISFREKMTIPDLYFINLAVADLILVADSLIEVFNLHERYYDIAVLCTFMSLFLQVNMYSSVFFLTWMSFDRYIALARAMRCSLFRTKHHARLSCGLIWMASVSATLVPFTAVHLQHTDEACFCFADVREVQWLEVTLGFIVPFAIIGLCYSLIVRVLVRAHRHRGLRPRRQKALRMILAVVLVFFVCWLPENVFISVHLLQRTQPGAAPCKQSFRHAHPLTGHIVNLAAFSNSCLNPLIYSFLGETFRDKLRLYIEQKTNLPALNRFCHAALKAVIPDSTEQSDVRFSSAV

>Mouse_GPER1_Q8BMP4

MDATTPAQTVGVEIYLGPVWPAPSNSTPLALNLSLALREDAPGNLTGDLSEHQQYVIALFLSCLYTIFLFPIGFVGNILILVVNISFREKMTIPDLYFINLAAADLILVADSLIEVFNLDEQYYDIAVLCTFMSLFLQINMYSSVFFLTWMSFDRYLALAKAMRCGLFRTKHHARLSCGLIWMASVSATLVPFTAVHLRHTEEACFCFADVREVQWLEVTLGFIMPFAIIGLCYSLIVRALIRAHRHRGLRPRRQKALRMIFAVVLVFFICWLPENVFISVHLLQWTQPGDTPCKQSFRHAYPLTGHIVNLAAFSNSCLNPLIYSFLGETFRDKLRLYVEQKTSLPALNRFCHATLKAVIPDSTEQSEVRFSSAV

>Gallus_GPER1_F1NWC2METYSASVPPVLCNSTTFNLSGSHLCNESLSSRLADKSEHQQYVIGLFLSCLYTIFLFPIGFVGNILILVVNISFREKMTIPDLYFINLAVADLILVADSLIEVFNLDEKYYDITIICTFMSLFLQINMYSSIFFLTWMSFDRYIALAKVMRSNLFRTMQHARLSCGLIWMASISAALVPFTAVHLQHTGEVYFCFADVREIQWLEITLGFIIPFVIIGLCYSLIVRVLIKAHKHRSLRLRRQKALRMIFVVVLVFFICWLPENVFISVQLLQKKSEPTSSSSPSFRHDYPLTGHIVNLAAFSNSCLNPLIYSFLGETFRDKLRLYIEQKTKMSTLHRFCQAALTSVIPDSNEQSEV

>Goldfish_GPER1_A0A109YKV0

MEEQTTTVIQIYVNDTKQFNASYDFNLTDVKESIDTYEFYVIGLFLSCLYTILLFPIGFIGNILILVVNLNHRDKMTIPDLYFVNLAVADLILVADSLIEVFNLNEKYYDYAVLCTFMSLFLQVNMYSSVFFLTWMSFDRYIALANSLSSSPLRTMQHAKLSCSLIWMASILATLLPFTIVQTQHTGEVHFCFANVFEIQWLEVTIGFLVPFSIIGLCYSLIVRTLMRSQKHRGLWPRRQKALRMIVVVVLVFFICWLPENVFINIQLLQGTADPSKRSDTTL

>Human_NPFF2_AF268899

MNEKWDTNSSENWHPIWNVNDTKHHLYSDINITYVNYYLHQPQVAAIFIISYFLIFFLCMMGNTVVCFIVMRNKHMHTVTNLFILNLAISDLLVGIFCMPITLLDNIIAGWPFGNTMCKISGLVQGISVAASVFTLVAIAVDRFQCVVYPFKPKLTIKTAFVIIMIIWVLAITIMSPSAVMLHVQEEKYYRVRLNSQNKTSPVYWCREDWPNQEMRKIYTTVLFANIYLAPLSLIVIMYGRIGISLFRAAVPHTGRKNQEQWHVVSRKKQKIIKMLLIVALLFILSWLPLWTLMMLSDYADLSPNELQIINIYIYPFAHWLAFGNSSVNPIIYGFFNENFRRGFQEAFQLQLCQKRAKPMEAYALKAKSHVLINTSNQLVQESTFQNPHGETLLYRKSAEKPQQELVMEELKETTNSSEI

**Supplementary Figure 4.** Phylogenetic analysis of *Lymnaea* mPR candidates. For the analysis, relevant PAQR sequences were used (4). The alignment was made with MUSCLE in the Molecular Evolutionary Genetics Analysis v7 software (3). The maximum-likelihood tree was made with the LG general amino acid replacement matrix and four discrete gamma models. Bootstrapping support for the tree was conducted with 1000 bootstrap replicates, the bootstrap values (%) are indicated at each branch point. All positions containing gaps and missing data were eliminated. There was a total of 228 positions in the final dataset. The *Lymnaea* sequences are marked by red arrows. Based on the analysis, *Lymnaea* mPR2 sequence, which was also identified as an adiponectin receptor by a recent study (5), was excluded from further experiments.

**
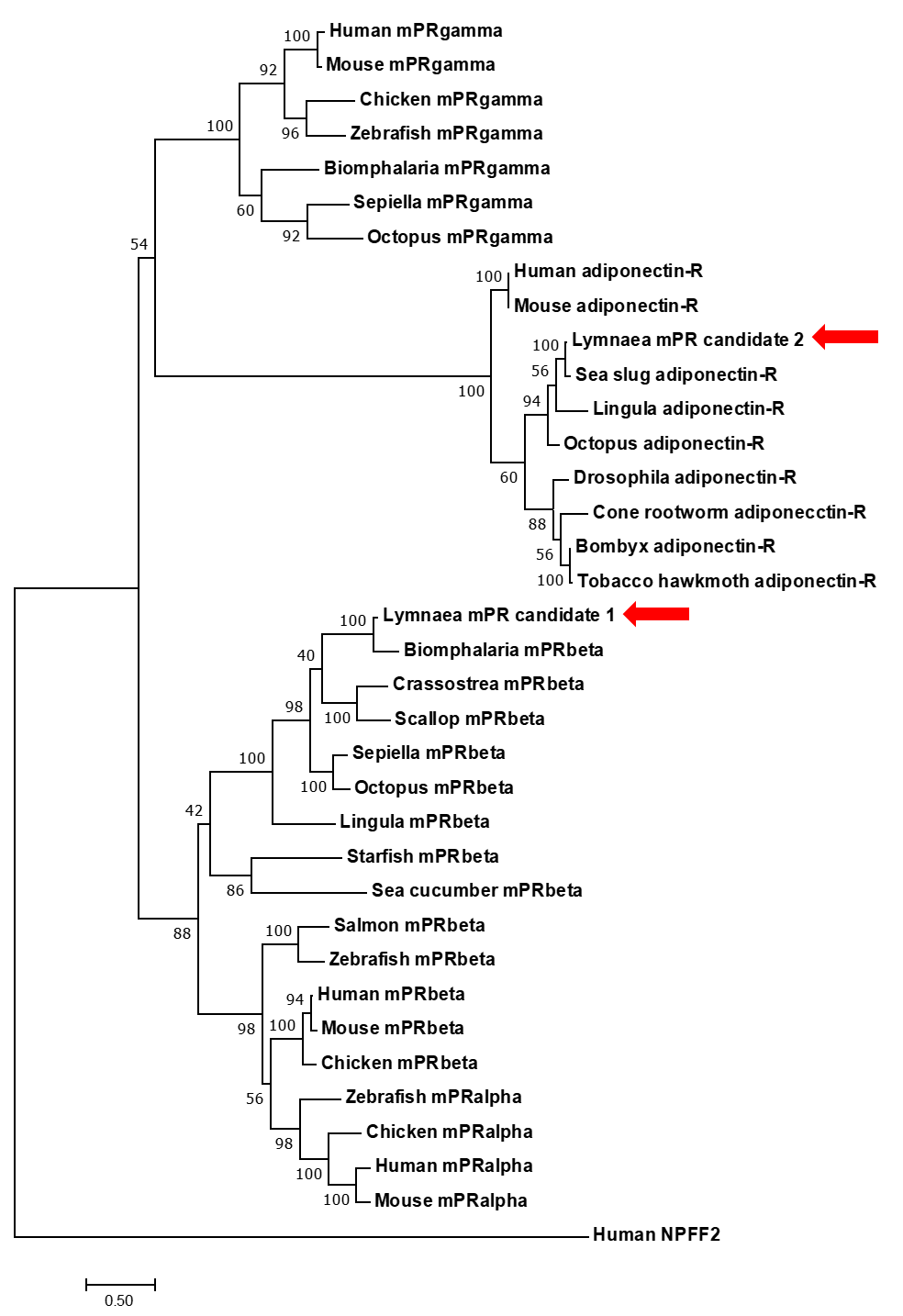
**

>Sepiella mPRbeta_AVA07271.1

MWHLMKPTVTKEQVPLLFHEPHVLSGFRHLNQPWSYYFLSLFQVHNECLNSWTHLIALILIIKKMFTFSMEVDLITDPYMWPLTSGILSIIILYLCSSLAHCLQSRSELAHYTCFMFDYAGIGLYGTGSIVLHYNYCMLPTYKLGALREWAITIGIGLGVVVCMCCSISKIRYKRPYPFVRKVWQLGSVGGIYAWLILPIAHRVFLFVISSQWDQGLPHHMEQMVWFLVAGFFFGSDIPQRFFPGKFDFVGHSHQLFHI

>Octopus mPRbeta_A0A6P7SN93_ XP_014774875.1

MVQLMKPTVSKEHVPLLFHEPHVLSGFRHIHQPWTYYFLSLFQIHNECLNSWTHLIAMLLVIKKMMTFSKEFSLITDPYMWPLTSGILSIVILYLCSSLAHCLQSRSELAHYTCFMFDYAGIGLYGIGSIILHYNYCMLPSFDIGILQSWIIPVGVLLGVGVCLCCSISKVRYKRPYPFIRKVWQLGSVGGIYALLILPIAHRVLLMILTSEWDKGLPHHIEQMIWFIMAGFFFGSDIPQRFFPGKFDFLGHSHQLFHICIMMVSWKQLDGIYEDIVAWKTLLYEKDPPTFSSTFGAIFLTIFINLIVVVIFTESAKQFIDQGKPREIKIDLISVKKRK

>Crassostrea mPRbeta_XP_019918893.1

MKHKGHISLLRPTTTKHDVPVLFQEPHVETGFRHPHQPWFYYICSIFQKHNECMNVWTHLIGLLLTVSRTIEFSNEYDLIGNPHMWPLSAGLITMILMYLCSTCAHCFSHKSELVHYTGFMIDYAGIGIFGLGSTIIHYAYCIHDTMLNSSLKMLSVPIGVILGALVCICCTVSKVFYKRPYPFTRKIWQIGSVGGIYTWLSLPILHRLFLDKDGEDVSLYHHTSQMIWFALAGFFFGSDIPQRFFPGKFDFVGHSHQIFHICIILVTQKQLDGVHLDIEKYHRTPAFVDEPSFMETFGAVISLTIVCFLNVFIFHNVVKYRLGKENVKQE

>Biomphalaria mPRbeta_XP_013077721.1

MIFLPPALDKDEIPILFHEPYVLTGFRPLHYPWTSYLLSIFQWHNELLNIWTHLLALIMVLIRASVWSTEFSLLSDPFMWPLSVGIITMIILYICSSGAHCLQNRSEVVHYTCFMCDYAGIGLYGFGSTMLHYWYCLHEDLLGSFSHQFAIPVGAVLAVIVCICCTISKTKYKRPYPFIRRVWQMSSVFAIYVWLIFPIWYRIWMYYHDGKWSSSFKNHIQQMLWFTVGGFFFGSDVPQRFCPGLFDIIGHSHQIFHMCIFMTTYEQMNALYLELTDDTVIIHKMEEPTLFNTWGMLAMVIVANIFVVYYFHCSVNNRLEQEKEQNLDVKNNKHMTENGGSCLHQENIQKSLVSKCKDKTQ

>Scallop_mPRbeta_A0A210Q2G6

MMLSSLAPTTTKKGVPLVFHEPHVETGFRTPHQPWSYYVYSLFQKHNECMNCWTHLVGLCLALSRAVDFAHNHRMLGDPHMWPLTAGLITMVLMYLCSTTAHCFSNRSELVHYTAFMIDYAGIGLFGVGSVILHHHYCHDDAWVNSWLQTNAVLVGMILGVIVCFCCSVSKVIYKRPYPIARKIWQVSSVVAIYTWLSLPILLRLYQYAAIGVWHSSLDHHIQQMIWFAVAGFFFGSDIPQRFFPGKFDFVGHSHQIFHLCIIMVTEKQLDGITHELERLSPKFANMALPTFWNSIVIIHFHVAQCNPPHVS

>Lingula_mPRbeta_A0A1S3GXV6

MQKVTAESSQNEVDSIEEADKRSSCLTPTTTFEDVPKVFREPNINTGFRQPYQPWYYYPASIFQVHNECMNVWTHIFAVILMTHKLVSFSKQLDFINDPYTWPMLAGILSSIILYLASSFAHCFQSKSEMFHYTCFMIDYAGIGLYGFGSVTVHFAYCMEEHLYQISWLKDMFVPVGGFLAVATCGCCTYAKYRYQRPYPRARKVWQVTGVCFLYIWLIIPVVDQVFNCFINGFRCDDTISPHTKQLAWFLASGFFYTSDIPQRFWPGKCDFIGHSHQFFHVCIMMTTFFQFDGVIRDLHNERDVFEQRPTPTLWNTFGPVALVLLLEIVFIIIFREKCKKKLKSKNE

>Starfish_mPRbeta_A0A8B7YE47

MKISIGYENNQPPGLRAEEVPLQFREPYVEIGYRKPYQPFSFYLKSFFQIHNESLNVWTHAIACVAIMFQGIRLCATLDMEHDPYAPMFKMLIFSCMTYLFLSTCAHLFQSINEVAHHTCFFIDYMGVSLYAFSAGMAHIHFCALPWVYDVTESFYLPLHWFLSFLVCFCCSYSKYRYKRPYPFARKVWQMSSVAAGYIITMFPVVMRLLFQGLSIFHDLALFYQVLSVASFLAGSFFFAAPFPQKFSPGNFDIVGHGHQLFHIFMALTSYMEVEAVYRDYVDRRSIYSNLFTPTPMIVYGYFVLLMIANMTVVFVFREKIKARIEREKEE

>Sea_cucumber_mPRbeta_A0A2G8K7I5

MKCEMKLHPISSLVCTDKRPKSPRREGQSFSVSRLDHHPCEMGYISDAMGVRKVFPRVPSNTKLAVEVPLLFQEPDIYHGYRDNYQPFSYYTGSAFWLHNESVNVWTHLIALMVLLRQTWSLCSNLDLLNDGHAQVFAVYCFGCCAYTLASALAHLYQSRDELTHHTCFYMDYAGVSLYGNATALACYHLASPSSFFHFWRGWFMPLNCFLSVTMCACCGFAKYKYRRPYPKTRKYWQIISSGLGYAQIISPVYYTLIHQLYNRQTTPLGQDFYLHLLQCALFIPGSLFFAVPYPQRFWPGLCDLFGHSHQLFHVFMSLMTYFQMKALYLNVIQKRDIHTDLSNPSFLTTFGYVLVVLVLDLVVVWVLRMKTYQKLKKEAKD

>Sepiella mPRgamma_AVA07272.1

MATRMVIGAKTSYHFRLMMKKIISCKSFEMISTYPLNGPLYHVDQIPAEFHESFILSGYRHPKSTFLQCVFSVFYRTNETGNFWTHFLPSCYFIYVICEKLSEDDYSLPFLAYLLACILFSLASSMAHMFFVLSDYARHICFFIDYGGLSLFSIGSAIAYRAYVFPEVLERTWFGYWYLPMAIFGALASTFFSCLSRFKKDCHTQQLLRIVAFSLPYLFDNLPVMVWLALCDFKQCLQSKFYHITQFMFCFIAAFLYMSHLPERLQPGRFDIWGHSHQIFHVCGILGTISQMKAIEIDMDLQKANIIKPCYYMYFHYSVEIMCAVLILNIIIIFIFSCMLKIEMKKKND

>Octopus mPRgamma_XP_014787281.1

MAARIVIAARITDHIKLTLKKLIRHRNLEMMVFQRILGPLCSVEQIPTEFRENFILTGYRYPECTIFQCVCSIFTATNETFNFWTHFLPALYFLYTICQTYSKDFLQSSYNSPLIAYLVTCILFPFASSMAHMFLSLSNNARHICFFIDYGAVSLYSFGSSIAYRAYAFPEVLEYTWFGKWYLPIAFFSALLSTLFSCQSRFSGKYEQIFRIGVFSWPYFFDNIPIFILFTPLEERFYSKFYHTKQFLYCFMAVLMYVSHFPERFKPGYFDIVLHSHQLFHIFGIISTFYQMRGIQIDMATRKNTITKQWFYNYFHYSISIVLVQLLLIALIILLYTCILNHKKAIKKKE

>Biomphalaria mPRgamma_XP_013087777.1

MLGMSVSTGLSGPIYYAHQVPEHFHEHYILRGYRHPKSSVTQCLLSVFDPTNETLNIWTHFLPTWYFVYVVYHLWWSIDFTNDVYSWPLLSYLLVCCAFPLASAVAHLFNVMSDCARHVCFFLDYSALSLFSFGVALIYRAYCFPSHILSNTTGCTWFKDHYVNLAAITAILCTFISCETRFMKPSAARKAIRLGAFAIPYLFDSIPIFYRILFPDVNEWSAEYLHKRQFLFALVAAFLYASHLPERLLPGIFDIIGHSHQLFHVSSILAVMDQLQAVLLDFKERRSFVEPCWQSREFSNSLGYLLNIFVINSIIIMIFTIRLLYLKHKMKCT

>Human_mPRalpha_Q86WK9

MAMAQKLSHLLPSLRQVIQEPQLSLQPEPVFTVDRAEVPPLFWKPYIYAGYRPLHQTWRFYFRTLFQQHNEAVNVWTHLLAALVLLLRLALFVETVDFWGDPHALPLFIIVLASFTYLSFSALAHLLQAKSEFWHYSFFFLDYVGVAVYQFGSALAHFYYAIEPAWHAQVQAVFLPMAAFLAWLSCIGSCYNKYIQKPGLLGRTCQEVPSVLAYALDISPVVHRIFVSSDPTTDDPALLYHKCQVVFFLLAAAFFSTFMPERWFPGSCHVFGQGHQLFHIFLVLCTLAQLEAVALDYEARRPIYEPLHTHWPHNFSGLFLLTVGSSILTAFLLSQLVQRKLDQKTK

>Mouse_mPRalpha_Q80ZE4

MAMAVAQKFNHLLSSLWHVGQKPPQPEPVFTVDRAQVPPLFWKPYIYAGYRPLHQNWCFYFRTLFQRHNEAVNVWTHLLAALALLLRLIGLAASVDFREDPHALPLFFIVLASFTYLSFSAVAHLLQAKSEFWHYSFFFLDYVGVAVYQFGSALAHFYYAIEPSWHDKVQAIFLPTAAFLAWLSCAGSCYNKYSQKPGLLGRIFQEAPSALAYVLDISPVLHRIIVSPLPAEEDPALLYHKCQVVFFLLAAAFFSTVMPESWFPGSCHIFGQGHQVFHVFLVLCTLAQLEAVTLDYQARRGIYEPLHARWPHNFSGLFLLTVASSSLTALLLSQLVRRKLHQKTK

>Chicken_mPRalpha_ NP_001157123.1

MAAVVAEKLSRLFISVRQVPQLLAPPVPTTVSSSEVPRVFWKPYIHTGYRPVHQTWRYYFSTLFQQHNEAINVWTHLVATLILLLRFQQLSQRVDFGQDPHAQPLLIIITASITYLTFSTLAHLLQAKSEFWHYSFFFMDYVGVAIYQYGSALVHYYYAIEPSWHEKIQGFFMPTAALLAWLSCAGSCYAKFRYHQSAGLLGRLCQEMPSGLAYLLDISPVVHRICTASPAERTDPALLYHKCQVLFFLIGAFFFSHPYPEKLLPGKCYFFGQSHQIFHVFLVLCTLAQIEAVVLDYESRRHIYSSLQGDLAHHFSALCVFTVTCSVLTAAYMARKVRDKLSFKED

>Zebrafish_mPRalpha_AAN78115.1

MATVVMEQIGRLFINAQQLRQIPRFLESAFPKLPCTVMVSDVPWVFRESHIITGYRPPDQNWRYYFLTLFQRHNESVNVWTHLLASLIILVKFQELSETVDFLRDPHAQPMFILLLAAFTYLGCSALAHLLSAKSEISHYTFYFLDYVGVAVYQYGSALAHFYYVVEEEWHAQVRTFFLPASAFLAWLSCTGCCYGKYASPKLPKFVHKLFQVVPSGLAYCLDISPVLHRIYRCYSSEHWCADQAVVYHCYQVLFFLISAYFFSYPHPERWFPGRCDFIGQGHQIFHVFLVLCTLVQIEAVRLDYTERRRLYEHLHGDLAHDAVALFIFTACCSALTAFYVRKRVKTYLEEKQE

>Human_mPRbeta_Q8TEZ7

MTTAILERLSTLSVSGQQLRRLPKILEDGLPKMPCTVPETDVPQLFREPYIRTGYRPTGHEWRYYFFSLFQKHNEVVNVWTHLLAALAVLLRFWAFAEAEALPWASTHSLPLLLFILSSITYLTCSLLAHLLQSKSELSHYTFYFVDYVGVSVYQYGSALAHFFYSSDQAWYDRFWLFFLPAAAFCGWLSCAGCCYAKYRYRRPYPVMRKICQVVPAGLAFILDISPVAHRVALCHLAGCQEQAAWYHTLQILFFLVSAYFFSCPVPEKYFPGSCDIVGHGHQIFHAFLSICTLSQLEAILLDYQGRQEIFLQRHGPLSVHMACLSFFFLAACSAATAALLRHKVKARLTKKDS

>Mouse_mPRbeta_Q80ZE5

MTTAILERLSTLSMSGQQLRRLPKILEEGLPKMPCTVPETDVPQLFREPYIHAGYRPTGHEWRYYFFSLFQKHNEVVNVWTHLLAALAVLLRFWAFVEAGALQWASPHTLPLLLFILSSITYLTCSLLAHLLQSKSELSHYTFYFVDYVGVSVYQYGSALAHFFYSSDQAWYELFWIFFLPAAAFCGWLSCAGCCYAKYRYRRPYPVMRKICQVVPAGLAFVLDISPVAHRVALCHLAGCQEQAAWYHTLQILFFLVSAYFFSCPVPEKYFPGSCDIVGHGHQIFHAFLSVCTLSQLEAILLDYQGRHEIFLQRHGPLSVYSACLSFFVLAACSAATATLLRHKVKDRLIKKDS

>Chicken mPRbeta_NP_001008462.1

MMTAILERLSTLSLSGPQLSRLPRLLEDGFPKMPCTVQEGEVPQLFREPYIHTGYRPTGQDWRYYFLSLFQKHNEVVNVWTHLLAALAVLLRFKAFVEGEQLPLDAWSLPLLIFVLSSVTYLTCSLLAHLLQSKSELYHYTFYFVDYVGVSTYQYGSALAHFYYSSDQAWYDKFWLFFLPAAAFCGWLSCAGCCYAKYRYRRPYPIMRKMCQVIPAGLAFILDISPVAHRVIVCHLGGCEEDAAWYHTYQILFFLISAYFFSCPVPEKYFPGSCDIVGHAHQIFHTFLAICTLSQLEAICLDYKNRQEIFLKRHRPFSIYLSCISFFGLVACSAITAYILRCRIKAILAKKDS

>Zebrafish mPRbeta_NP_899187.1

MSSGVLGRLSTLTLSLQQLGQLPHLSNWLPRLPRRQATVHASEVPSLFREPYILSGYRPVHQEWRSYFCSLFQCHNELLNVWTHLLAIPAVLLQFSFFAGAWGLTLNLASLPLFLYVLSSLTYLSFSVAAHLLQSHSELAHYSLFFVDYVGVAVYQYGCSMGHYFYCSEPEWRHSLVGVLFLPGAAMLAWLSCASCCYSKFRYRRPYPFHRKICQIIPTSLAYLLDISPVAHRLLTKSWDEPVLVFHAMQVAFFLLAALFFSCPVPERFFPGRCDIVGHGHQIFHIFLVLCTMCQLEAMFRDFLVHQQSVVDAHGEHFILLAGGSFFLLVLCSILTAVLMRGAVQRQLRKKD

>Salmon_mPRbeta_A0A1S3SBF1

MSSGALGRLSTLTLSVKQLSRLPHLSDILPSSLPSLPSPSPTVPVSCVPTLFQEPYILSGYRPVGQDWRCYLLSLFQRHNESLNVWTHLLAGPLVLLRVWAYGLSLDSASLPLCLYILSALTYLCCSVAAHLLQSHSELAHYSLFFLDYVGVSVYQYGCSLAHYFYCSEPAWRESTVGFWFLPGAALLGWLSCASCCFAKSRYRRPYPLRRKICQLIPTSMAYFLDISPVAHRLATVPWGQDPALPLHALQMAFFLLAALFFSCPVPERFFPGHCDIMGQGHQIFHLFLSLCTLCQLEALFLDYGSRRDTVLQLYGERQLWFAGVSFLALALCSALTAVVMRRHVQRRLEKCETVKQ

>Human mPRgamma_NP_060175.3

MLSLKLPRLFSIDQIPQVFHEQGILFGYRHPQSSATACILSLFQMTNETLNIWTHLLPFWFFAWRFVTALYMTDIKNDSYSWPMLVYMCTSCVYPLVSSCAHTFSSMSKNARHICYFLDYGAVNLFSLGSAIAYSAYTFPDALMCTTFHDYYVALAVLNTILSTGLSCYSRFLEIQKPRLCKVIRVLAFAYPYTWDSLPIFYRLFLFPGESAQNEATSYHQKHMIMTLLASFLYSAHLPERLAPGRFDYIGHSHQLFHVCVILATHMQMEAILLDKTLRKEWLLATSKPFSFSQIAGAILLCIIFSLSNIIYFSAALYRIPKPELHKKET

>Mouse mPRgamma_NP_083024.1

MLSLKLPRLFRIDQVPQVFHEQGILFGYRHPQSSATACILSLFQMTNETLNIWTHLLPFWFFVWRFMTALYVTDIQNDSYSWPMLVYMCTSCVYPLASSCAHTFSSMSKNARHICYFLDYGAVNLFSLGSAIAYSAYTFPDALVCSTFHECYVALAVLNTILSTGLSCYSRFLELQKPRLCKLLRVLAFAYPYTWDSLPIFYRLFLFPGESSRNEAMLYHQKHMGMTLLASFFYSAHLPERLAPGRFDYIGHSHQLFHVCVILATHLQMEAILLDKTLRREWLLATSRPFSFPQIAAAMLLCIIFSLSNIIYFSAALYRIPEPELHEKET

>Chicken mPRgamma_XP_015147701.2

MLSLKLPRLLSIHQVPKGYQEQGILCGYRPPRISAADCVLSAFQMTNETLNIWTHFLPAWYFVWMLVGRLWGPGGRDPPAWPLLAYLLSCCIYPLASSCAHTFSPMSARARHVCYFFDYAALSMYSLGSALAYSAYVFPEEWVGSIFHCCYVPVAVLNTVLSTSLACYSRFLELERPWLSKASRTLAFVYPYLFDSIPLFYRFYVCAARSCADPTVAAHYRHTAFAFLTCFIFATHLPERLAPGHFDYIGHSHQVFHVCGILGTHFQLEAILMDMSERQARLPATSLLQALAPMGTCMAVGLAVIAHCSAQLCRAPEPSHREKLHGQ

>Zebrafish mPRgamma_Q7ZVH1.2

MLNLIKLPQVFTINQVPKVFHEDGIISGYRHPCSSAKDCVLSLFQLTNETLNIWTHFLPTWFFLWKLLTVVLVLEDWRDPFIWPFLVFLLSCCVYPLASSCAHTFSTMSERARHICFFFDYGALSFYSLGSAIIYSSYSFPDKWVNGTFHLNYVSIAVVNSIISTALACYSRLGLPFLEYNCHSIKRPSGKLDQKLCKCLRIIAFVYPYLFDNIPLFYRIFVCAGEGCTVNEANTVHYQHTSLAFFTGFLFATHLPERLAPGSFDYIGHSHQLFHVFAIIGTYFQMTAIELDMAARKQWLHAHLPPVTFLNTVGAAFFSVVSGLCIVYVFSLSLFSTRGVKNKSF

>Sea_slug_adiponectin-R_A0A433TR46

MQKKNGDVSMSHKRQAFAEEVEHLGEDNSSMAAFETDCGGDSLDSFPHLEEKAPSWQQLKRQAGAGANGYTGYKTDELGALLGDDMSPEDDKLPLDLDEPLPGAETSLLNVPSKCAEQAEEFVKKVWAAGWNVAHHHTLPDWLKDNDFLLRGHRVPTNSFMACFKSIFRIHTETGNIWTHLLGMIAFLGIACYFLSRPSIEIQWQEKAVFSAFFMGAILCMTFSWVFHTVYCHSERVGRFFNKLDYCGIALLTIGSFVPWLYYSFYCRLEPKVTYLALIMFLGTICIVVSMWDKFAQPQYRPLRAGVFVALGLSGVVPAMHYVITDGFWHAIDFAALGWLVLMAMLYIVGAVIYAARIPERIFPGKFDIWFQSHQIFHVFVLAAAFVHYHGISEIANYRLTLGDCIAREF

>Octopus_adiponectin-R_A0A0L8HT40

MASVSSSDSSSSREKECNPPNNSPSSSPDLGPSLLSKINSHLDHETDPNLDPDTDYHNLADPVLLGQSDSLLGQNDGLMLDSGDDFTDAEDDLSLPTNRAVAKSEITRMLPAVSEAIAPIDEGQDNVLPHRSKVEIISTTSRAAVEHAEEFVHKVWEAGWRVAHHASLPDWLKDNEYLMKGHRPPTNSFAACFKSIFRIHTETGNIWTHLLGFIAFIGVAIYFLTRPSVEIQWQEKAVFSAFFAGAILCLGFSWIFHTVYCHSERVGKFFNKLDYCGIALLTMGSFVPWLYYSFYCRLEPKIAYLILIFVLGISCIVVSLWDKFSQPQYRGIRAGVFIGLGLSGIIPAMHYVITDGFYHAINCASLGWLALMALLYISGAIIYAVRIPERLFPGKFDIWFQSHQIFHVFVVAAAFVHYHGITAIANYRLTLGDCLETRNS

>Lingula_adiponectin-R_A0A1S3JUM8

MSVSPTTEVGFPRPTQQERTTVTKRNNTQTASIPSTDSNTSHTQSMSKPSTSNSRSSTNTALVEDSPQLISLADTEDLDDPDNGLSGDEVRKKTEEGKLLQDANYEHNSENDDEEEAGEEDALNPAVDIIHAIGRRMEQAEEFVKKGWWEIVHHNHLPNWLKDNEFLLHGHRPQLNSFSDCFKSVFRIHTETGNIWTHLLGFIAFIGIAGYFLSRPTIEVQWQEKAVFSAFFLGAILCLGFSWVFHTVYCHSERVGRLFNKLDYCGIALLTMGSFVPWLYYSFYCELGPKIAYLVLIFTLGGFCILVSLWDKFAEPRFRAVRAGVFVALGLSGVIPACHYVIQNGFWDAVNNVAMGWLVLMAFLYIFGAFIYAIRFPERIFPGKCDIWFQSHQIFHMFVLAGAFVHYHGISEIANYRLTLGDCVEAVVA

>Drosophila_adiponectin-R_Q9VCY8

MDSATNLLEQQGSAADVSGGSHPAEVEVTTQARATFGMDAEGHATGEAVTTTTATLRREGSDEDIFEQVQMILRKRRGWGPEDSLSPNDLDILEYDDELVEEDDAGCPLPSTPEDTQLIEAEMTEVLKAGVLSDEIDLGALAHNAAEQAEEFVRKVWEASWKVCHYKNLPKWLQDNDFLHRGHRPPLPSFRACFKSIFRVHTETGNIWTHLLGCIAFIGVALYFISRPSVEIQTQEKIVFGAFFIGAIVCLGFSFAFHTLSCHSVEMGRLFSKLDYCGIALLIMGSFVPWLYYGFYCHYQPKVIYLSVVSILGILSIVVSLWDKFSEPALRPLRAGVFMSFGLSGVIPAIHYSIMEGWFSQMSRASLGWLILMGLLYILGALLYALRVPERWFPGKFDIWGQSHQIFHILVIAAAFVHYHGISEMAMYRVMYSECTVPIEPITF

>Bombyx_adiponectin-R_A0SY07

MDCDAGKDGIPMRRALPLKWTSSTKSSQKKKKDVRCLQHRRINICWMPKWLKFLKAGVLSDEIDLGALAHNAAEQAEEFVRKVWEASWNVCHFRHLPRWLQDNDYLHKGHRPPLPSFSACFASIFRIHTETGNIWTHLLGCVAFIGVAIYFLSRPSIEIQMQEKVIFGVFFVGAIVCLGFSFAYHTLYCHSEMVGKLFSKLDYCGIALLIMGSFVPWLYYSFYCHYRPKIIYLSVVVVLGILSIIVSLWDRFSEPRLRPLRAGVFMGFGLSGIVPAIHYGITEGWFSQVSKASLGWLVLMGLLYILGAMFYALRVPERWFPGKCDIWFQSHQIFHVLVIVAAFVHYHGISELASYRVTVGECSMPPTSMAF

>Tobacco_hawkmoth_adiponectin-R_A0A922CYX8

MWEVDSDNGSHSASLDGLRRRQGWDPEAESLASQMDELDEVLAEEEEGCPLPSTPEDQHLLDAEMAEVLKAGVLSDEIDLGALAHNAAEQAEEFVRKVWEASWNVCHFRHLPRWLQDNDYLHKGHRPPLPSFSACFASIFRIHTETGNIWTHLLGCVAFIGVAIYFLSRPSIEIQMQEKMIFGVFFIGAIVCLGFSFAYHTLYCHSEMVGKLFSKLDYCGIALLIMGSFVPWLYYSFYCHYRPKIIYLSVVVVLGILSIIVSLWDRFSEPRLRPLRAGVFMGFGLSGIVPAIHYGITEGWFSQVSKASLGWLVLMGLLYILGAMFYALRVPERWFPGKCDIWFQSHQIFHVLVIVAAFVHYHGISELASYRVTVGECSMPPSSIVF

>Cone_rootworm_adiponecctin-R_A0A6P7GCU7

MSDVLKAAVLEILLDNAEALGDDLDITALAHNAATQAEHVVRKMIQAGWSVCHFRNLPAWLQDNDFLHHCHRPPLPSFRACFKSIFRLHTETANIWTHLLGCVAFIGIAIYFLMRPNTEIEIQEKVVFGAFFAGAIICLGMSFMFHTVNCHSQFIGKLFSKLDYCGIALLIMGSFVPWLYYGFYCHFRPKVVYLSVVCALGITSIFVSLWDKFSESGWRPFRAAVFMTFGLSGIVPAIHYGIVEGWFNYVSQKYLGWLVLMGMLYIVGAMFYALRIPERWFPGKCDIWLHSHQIFHVFVLGGALVHYHGISEMAMHRVTIGQCEIPDTPIY

>Human_adiponectin-R_Q96A54

MSSHKGSVVAQGNGAPASNREADTVELAELGPLLEEKGKRVIANPPKAEEEQTCPVPQEEEEEVRVLTLPLQAHHAMEKMEEFVYKVWEGRWRVIPYDVLPDWLKDNDYLLHGHRPPMPSFRACFKSIFRIHTETGNIWTHLLGFVLFLFLGILTMLRPNMYFMAPLQEKVVFGMFFLGAVLCLSFSWLFHTVYCHSEKVSRTFSKLDYSGIALLIMGSFVPWLYYSFYCSPQPRLIYLSIVCVLGISAIIVAQWDRFATPKHRQTRAGVFLGLGLSGVVPTMHFTIAEGFVKATTVGQMGWFFLMAVMYITGAGLYAARIPERFFPGKFDIWFQSHQIFHVLVVAAAFVHFYGVSNLQEFRYGLEGGCTDDTLL

>Mouse_adiponectin-R_Q91VH1

MSSHKGSAGAQGNGAPSGNREADTVELAELGPLLEEKGKRAASSPAKAEEDQACPVPQEEEEEVRVLTLPLQAHHAMEKMEEFVYKVWEGRWRVIPYDVLPDWLKDNDYLLHGHRPPMPSFRACFKSIFRIHTETGNIWTHLLGFVLFLFLGILTMLRPNMYFMAPLQEKVVFGMFFLGAVLCLSFSWLFHTVYCHSEKVSRTFSKLDYSGIALLIMGSFVPWLYYSFYCSPQPRLIYLSIVCVLGISAIIVAQWDRFATPKHRQTRAGVFLGLGLSGVVPTMHFTIAEGFVKATTVGQMGWFFLMAVMYITGAGLYAARIPERFFPGKFDIWFQSHQIFHVLVVAAAFVHFYGVSNLQEFRYGLEGGCTDDSLL

>Human_NPFF2_AF268899

MNEKWDTNSSENWHPIWNVNDTKHHLYSDINITYVNYYLHQPQVAAIFIISYFLIFFLCMMGNTVVCFIVMRNKHMHTVTNLFILNLAISDLLVGIFCMPITLLDNIIAGWPFGNTMCKISGLVQGISVAASVFTLVAIAVDRFQCVVYPFKPKLTIKTAFVIIMIIWVLAITIMSPSAVMLHVQEEKYYRVRLNSQNKTSPVYWCREDWPNQEMRKIYTTVLFANIYLAPLSLIVIMYGRIGISLFRAAVPHTGRKNQEQWHVVSRKKQKIIKMLLIVALLFILSWLPLWTLMMLSDYADLSPNELQIINIYIYPFAHWLAFGNSSVNPIIYGFFNENFRRGFQEAFQLQLCQKRAKPMEAYALKAKSHVLINTSNQLVQESTFQNPHGETLLYRKSAEKPQQELVMEELKETTNSSEI

**Supplementary Figure 5.** Phylogenetic analysis of *Lymnaea* mAR candidates. For the analysis, relevant invertebrate and vertebrate GPRC6A and ZIP9 sequences were used. Moreover, since GPRC6A is a member of family C of GPCRs which also includes the metabotropic glutamate receptors (6), invertebrate and vertebrate metabotropic glutamate receptors were also included. The alignment was made with MUSCLE in the Molecular Evolutionary Genetics Analysis v7 software (3). The maximum-likelihood tree was made with the LG general amino acid replacement matrix and uniform rates. Bootstrapping support for the tree was conducted with 1000 bootstrap replicates, the bootstrap values (%) are indicated at each branch point. All positions containing gaps and missing data were eliminated. There was a total of 200 positions in the final dataset. The *Lymnaea* sequences are marked by red arrows. Based on the analysis, we supposed that *Lymnaea* GPRC6A candidate was actually a glutamate receptor, but we did not exclude it from further experiments.


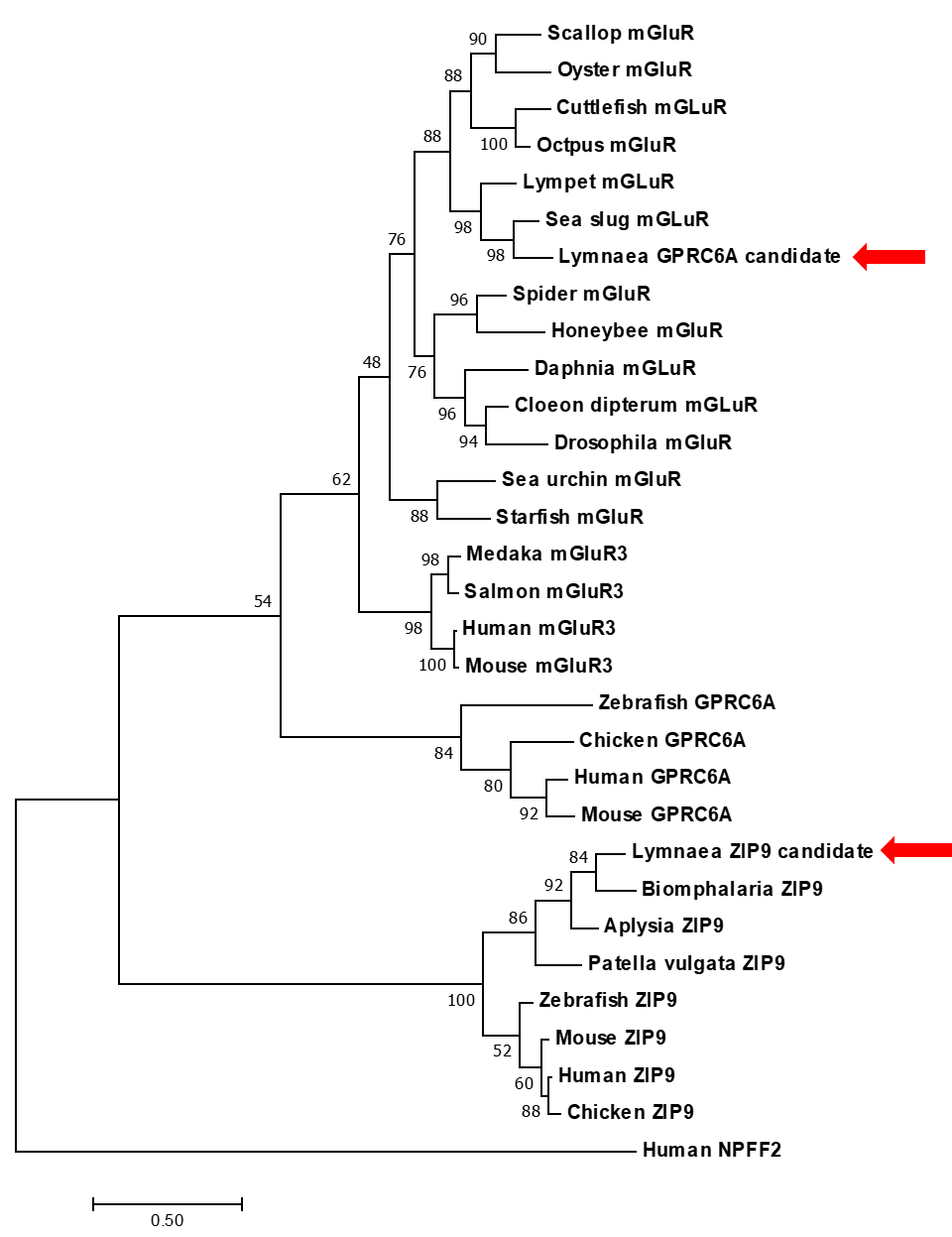


>Human_GPRC6A_NP_683766.2

MAFLIILITCFVIILATSQPCQTPDDFVAATSPGHIIIGGLFAIHEKMLSSEDSPRRPQIQECVGFEISVFLQTLAMIHSIEMINNSTLLPGVKLGYEIYDTCTEVTVAMAATLRFLSKFNCSRETVEFKCDYSSYMPRVKAVIGSGYSEITMAVSRMLNLQLMPQVGYESTAEILSDKIRFPSFLRTVPSDFHQIKAMAHLIQKSGWNWIGIITTDDDYGRLALNTFIIQAEANNVCIAFKEVLPAFLSDNTIEVRINRTLKKIILEAQVNVIVVFLRQFHVFDLFNKAIEMNINKMWIASDNWSTATKITTIPNVKKIGKVVGFAFRRGNISSFHSFLQNLHLLPSDSHKLLHEYAMHLSACAYVKDTDLSQCIFNHSQRTLAYKANKAIERNFVMRNDFLWDYAEPGLIHSIQLAVFALGYAIRDLCQARDCQNPNAFQPWELLGVLKNVTFTDGWNSFHFDAHGDLNTGYDVVLWKEINGHMTVTKMAEYDLQNDVFIIPDQETKNEFRNLKQIQSKCSKECSPGQMKKTTRSQHICCYECQNCPENHYTNQTDMPHCLLCNNKTHWAPVRSTMCFEKEVEYLNWNDSLAILLLILSLLGIIFVLVVGIIFTRNLNTPVVKSSGGLRVCYVILLCHFLNFASTSFFIGEPQDFTCKTRQTMFGVSFTLCISCILTKSLKILLAFSFDPKLQKFLKCLYRPILIIFTCTGIQVVICTLWLIFAAPTVEVNVSLPRVIILECEEGSILAFGTMLGYIAILAFICFIFAFKGKYENYNEAKFITFGMLIYFIAWITFIPIYATTFGKYVPAVEIIVILISNYGILYCTFIPKCYVIICKQEINTKSAFLKMIYSYSSHSVSSIALSPASLDSMSGNVTMTNPSSSGKSATWQKSKDLQAQAFAHICRENATSVSKTLPRKRMSSI

>Mouse_GPRC6A_NP_694711.1

MALLITVVTCFMIILDTSQSCHTPDDFVAITSPGHIMIGGLFAIHEKMLSSDDHPRRPQIQKCAGFEISVFLQTLAMIHSIEMINNSTLLSGVKLGYEIYDTCTEVTAAMAATLRFLSKFNCSRETVVFQCDYSSYMPRVKAVIGAGYSETSIAVSRMLNLQLMPQVSYESTAEILSDKIRFPSFLRTVPSDFYQTKAMAHLIRQSGWNWIGAITTDDDYGRLALNTFAIQAAENNVCIAFKEVLPAFLSDNTIEVRINQTLEKIIAEAQVNVIVVFLRKFHVFNLFTKAIERKISKIWIASDNWSTATKIITIPNVKKLGKVVGFAFRRGNTSSFHSFLQTLHMYPNDNNKPLHEFAMLVSACKYIKDGDLSQCISNYSQATLTYDTTKTIENHLFKRNDFLWHYTEPGLIYSIQLAVFALGHAIRDLCQARDCKKPNAFQPWELLAVLKNVTFTDGRNSFHFDAHGDLNTGYDVVLWKETNGLMTVTKMAEYDLQRDVFITTNQETKHEFRKLKQILSKCSKECSPGQMKKATGSQHSCCYECVSCPENHYSNETDMDHCLLCNNETHWAPVRSTTCFEKEVEYLDWDDSLALLLIALSLLGIAFVLAIGIIFTRNLKTPVVKSSGGLVVCYVMLICHALNFASTGFFIGEPQDFACKTRQTLFGVSFTLCVSCILTKSLKILLAFSFDPKLTMFLKCLYRPVPIVLTCTGIQVVICTLWLVLAAPSVEENISLPRVIILECEEGSALAFGTMLGYITVLAFICFVFAFKGRKLPENYNEAKFLTFGMLIYFIAWITFIPVYTTTFGKYLPAVEIIVILISNYGILCCIFFPKCYIILCKQKTNTKSAFLQMVYNYSAHSVDSLALSHVSLDSTSYDTATTNQSPGNKMTACQNDNHLPAQVLPHTGTAKTIKASKTLRQKRSSSI

>Chicken_GPRC6A_XP_040522589.1

MALFSLVLIPFVISSDAASACQNTDDFVGASSPGDIIIGGLFAVHSEMLQPEEHPIKPVIQNCAGFEIQIFLQTLAMIHAIEMINNSTLLSGVTLGYEIYDTCAEVTKAMASALRFLSKSNTSKDIVEFKCNYSDYVPRIKAVTGASYSEVSMAVSRLLALQLIPQVSPASSAEILSDKIRFPSFLRTIPSDFHQTRAMAHLICESGWNWIGVIATDDDNGRFALESFGIQAMANSVCIAFKEMLPAYLSDNTFHTKVDRAVEKIVKETRVNVIVVFMRQFHVLKLFKKAIERNVKKIWIASDNWSTAVKISTMPNIRKLGTVVGFGFKNKDLSTFQDFLRNLHDRPTENNKFLLEYIMLLSVCAHLDNYDFQMCISSQSQYDLMQNVENNHQIWRDDFLNANIEPGFIHSTILAVYAIAHAIKGQCKDRNCKNPSAFAPWELLEELKKVIIIDDDKEIKFDSKGDLSSGYDVLLWKEVDGRMEITTMAEYDPENGYFIFEDEEKKKEFLDLKKVPSTCSQHCRPGQMKKVTESPHTCCYECVYCPENHYSNQTDMDYCYRCHNKTYWAPVNSTTCYRKTIHFLGWTDWFAIFLLLLSAFGVVLIFSISAIFTKNLSTPVVKASGGLTVCYIILLSHFFIFLSTVFFIGEPTEFKCRTRQALFGISFALCISCILIKSLKILLAFSFDPKLQNFLKCAYKPITIVFICTGIQVIICTFWLIFRTPFVKQNFSIPRAIILECNEGSVVAFGIMLGYIAALAFICFICAFKGRKLPENYNEAKFITFGMLIYFIAWIVFIPVYVTTFGKYLPAVEIIVILISNYGILCCTFFPKCYIIIYKQETNTKSAFLKMIYTYSSKSVGSIAVSQISLDSKSSSSRITESDSCNAEKSSVNGNCHFQVSGQTPVKEKAVPKRATRTLSRKRLSSI

>Zebrafish_GPRC6A_AAI63279.1

MDLMSFILLWAGLMKVAEASIAQFSQLGASAPGNIIIGGLFPIHEAVVPVNYTGNNSISAPEHPDCIRFYTKGLNQALAMINAVEMANKSPMLSSLNITLGYRIYDTCSDVTTALRAVHDIMRPFSDCESPEDSSQPVQPIMAVIGTTSSEISIAVARDLNLQMIPQISYASTATILSDKSRFPAFMRTVPSDEYQTCAMAKLLKSNKWSWVGIIITDGDYGRSALEGFIQHTETEGICIAFKAILPDSLADQQKLNTDIENTLNIIENNPKVRVVISFAKSSQMQLLFKGLQSRNISNNMVWVASDNWSTAKHILNDGSITDIGKVLGFTFKSGNFTSFHQYLKNLQFESEDEMNNSFLKEFLKLNAGNASNTVLELMKSTNLDKIFSIEMAVTAVANAVAKLCAERQCQDSTALQPWELLRQLRSITFENGGEMYKFDANGDINLGYDLFLWEGDQSDEHADDIIAEYDPTKGGFHYIHNDLSEIKKVVSRCSNSCQPGQYKKTAEGQHTCCYECLTCVENHYSNITDADECSPCDSESMWSLANSTECHPKVFEYFDWNSGFAIVLLILAALGVLLLFFMSALFFWQRHSPVVKAAGGPLCHLILVSLLGSFISVVFFVGEPSDLTCRARQVIFGFSFTLCVSCILVKSLKILLAFEMNFELKELLCMLYKPYMIVSVGMGVQIIICTVWLTLYKPFKDKEVQTESILLECNEGFYVMFWLMLGYIALLALFCFTFAYIGRKLPQKYNEAKFITFSMVICLMAWIIFIPIHVTTSGKYVPAVEMVVILISNYGILSCHFLPKSYIILFKKEHNTKDAFMKNVYEYARKSAENIKGLTGTEPQFKQENSVYTISNLSFVPEEKHE

>Sea_slug_mGLuR_A0A3S1BQ95

SGNSTKKSLVATIKGEIILGGLFPVHDKGDNMCGDINLDRGIERLEAMLYTIDEINANPDLLPGITLGASVLDTCGRAPYALEQSLEFIRASFTSLDPTEFFCKDGSEAKPKSAPTTVVGVVGGSYSTVSMQVANLLRLFKIPQISYASTSASLSDKQRYDYFIRTVPPDTLQAKALVDIVQEFNWTYVSIVHSEGEYGESGIDFFKHEAKAKNICIAAHKEISLRATNATYDEVIKELLDKPEARVVIVFVRSEDATGLLNAASRKGLAGKFVWIASDGWGNRLAPVKNNPLVAQGAITLELQSTAISGFEDYFMNLNPRSNHRNPWFVEYWEEEHKCTWNGISMPSSPAEVNFHTRRRPCRGNERINRRITSQENKVQFIYDAVYAFAHALHDMQQDVCPTGQGLCPGMVNIDGEKLLRNYLLNVSFDDGYGAQVSFDNNGDAPGRYMIMNYQRNRRTREYEYRVVGTWNSGLQVNHSDIIWAGDTKDVPRSRCSDPCQEGEIKIMQKGEQCCWICTRCNPWEYIKDEKTCESCDTGLWPYPNKTGCFSLEVQHMTWTDLHAVIPMALSALGIMATSFVISLFVMFNSTPVVMASGRELSYMLLGGCVFCYFNTFVLLAPPSPVTCAVQRFGVGFGFSVIYSALLIKTNRISRIFESARRSAKRPPFISPKSQIVMTLILILIQVLFTFVWLVLEPPGTKLSYPDSREPMVILKCSSDDISFLVSLIYNMLLIIVCTVYAVKTRKIPENFNESKFIGFSMYTTCIIWLAFVPIYFGTLNSFKIQVTTLCVSISLSASVALLCLFLPKVYIIVFQPQKNVRKLTMNSASYKMAPTASSTMGNNHSSAPMPMTENLRLQYHHPRVHIGGVITETETDRDSLASLDTWTFAVCEYETYRNTWKIVYVNFTGIAGQLHLHNSTFVERVVQRYLEVSESVDLKLEIVKDSTSGVGTNTTLPPLQGSGGTRSVIDGAGQGFKFKFKFTS

>Lympet_mGLuR_V3ZLI4

MPNRTTKSIKSAFIAGDIILGGLFPVHHKGVGEQPCGEINPDRGIERLEAMLFTIDEINNNNNILPGITIGASLYDTCARGTYALEQSLEFIRASFYSLDTSEFVCSDGSQAKAKFSPTKVTGVVGGSYSTVSMQVANLLRLFKLPQVSYASTSASLSDKTRYDYFLRTVPPDTLQARALVDIVQEFNWTYVSTVSSEGEYGSSGIDYFQREARAKNICIAANVKIPAKSTNSTFDRTIRDLLEKPEAKVVIVFVRIEDAKGLLDAATRFNLSGKHVWVASDAWGRQDKPVKDNLLAGQGAITLELQSTPIKTFENYFRNLNPRTNIRNPWFREYWEAVNKCSWRENTNKHATRNIRYCTGAEKLNRKIHQQEGKVQFIYDAVYALAIALDKMQRSLCPNTTKLCPEMERIDGEHFKDFLLNVSFNDNYGAHVQFDKNGDALGRYTIMNYQRNRTTRHYEYQEIGRWSKTLNLDHSKIIWAGGTDKIPTSRCSEPCKEGEIKNMRQGDPCCWICTKCQPWEYVKDERTCESCGTGGWPYDNKTGCYSLVIEHMTWTSIYAIVPMCLAAVGIMCTNFVILLFILYNNTPVVMASGRELSYMLLCGCLFCYLMTFVLLAMPSVIVCALQRFGVGFGFSIIYSALLTKTNRISRIFESARRSAKRPPFISPKSQILLALILISIQILFTAVWLLIETPGIRLHIPDERKPLIILKCKSDDISFLVSLVYNMLLIIICTVYAVKTRKIPENFNESKFIGFAMYTTCIIWLAFVPIYFGTLNSFKIQITTLCVSISLSASVALLCLFFPKVYVIVFQPQKNVRKLTMNSASYKMAQTGTTGTTNNYGKKSILIEICHCTVFLSASVP

>Scallop_mGluR_A0A210QZW7

MAVSYRSRLLWGVVLILFYKIYLLNCIITFHGNDTGKLSRSAESANIKGDIILGGLFPVHQKGDDGAECGAINLQRGVQRLEAMLFTVDRINSDEEVLPGIILGAEVFDTCSRGTYALERSLEFIRASFANLDSADFKCDDGSIAKANATPRAIAGVIGGSYSSVSIQVANLLRLFKLPQISYASTSADLSDKKRYDYFVRTVPPDGFQAKAMADIVQLFNWTYVSTVASEGDYGQSGIESFQNEARSRNICISISIKIISNSNQDTFDTAIEDLDSKKNAMIVVLFLRVEDARNLLIAAKRKQLYNRFTWIASDGWGMQDKPVLGNEDMAEGAMTIELDSTLMPEFDTYFLKLKPGTNKRNPWFKEYWETVHECKFHGKSDLPITDQVVKNCTGQEKKLEAYQQETKVQFIYNAVYAMARSLDRMQRDVCPSTTKLCENMEKIDGERLLKDYILNTSFSDGYGATVQFDSKGDALGRYRIMNYQKNSKTKKYEYVVVGNWSMSRSLQLDVDRLVWSSGATGVFPESRCSQPCNFDQYKYIGKSGDSCCWVCITCQDFEYLKDEFTCEDCGRGRWPNKNKRDCHVLPKQYMQWDTVYALVPMVLACVGLIATLAVILTFMKFHDTPVVMASGRELSYFLLSGCLMCYFITFILIAKPSTIMCAIQRFGVGFGFSVIYSSLLTKTNRISRIFDSARRSARRPPFISPKSQIVITCILILIQILFTIVWLVIEPPGTRIYLPNGKRNEVILKCKTDDISFLVSLMYNMLLIIVCTLYAIKTRKIPENFNESKFIGFAMYTTCIIWLAFIPIYFGTLNSFQVQITTLCISISLSATVALLCLFTPKMYIIVFQPGKNVRRLTMNSASYNKKPQTTTSSILTSNNHDAFSERIKLSVNYDQRFGVTTTAEKDDKEKDTPASSL

>Spider_mGluR_A0A8T0EZW9

MTTEVTSFRGGGNRLPASHLIGIASIILFFLGLGQTRKAILEGDVMLGGLFPVHQKGLKTKCGAIFKDRGIQRLEAMLFAVEKINSDPTLLEKIKLGAIILDTCSSDSYALNQSLEFIRASINTVDATTFECADGSAPTMKFTTKAITGVVGGSYSEVSLQVANLLRLFKIPQISPASTGTSLSDKTRYDFFARTVPPDTFQAIALVDLVQSFNWSYVSLVSSEGQYGDSGMTAFQKEARSRNICIAVNEKVPHSATEEVFDSIYQNLLQKISARGVVLFTRAEDAAGVLRAAMRSRKHVFTWVASDGWGKQEKLVEGLERAAEGAITVELASTEIAEFNDYMKNLTPTTNIRNPWFEEYWESVFSCTLPQNVKPNSNSSVVVCSPNLRLDESVGYNQESKVQFVVDAVYAFAHALDKVWRDLCEPKGEAHCHEMRTLDGATLYLDYILNVSFTDLAGNQVKFDRQGDGLGRYTIYNYQRDDNSSAYHYKVVGKWFDGLHMDLNDVKWPSGRLTSSICSEPCGIGEVKITQSGDVCCWICHKCHPWEYVVDEFTCMDCGHGRWPYPHKQSCYDLDQQHMRWDSLFAIVPMAIATFGILLTFWVMVVFFRNNDTPIVKAAGRELSYLLLTGITLCYIMTFVMLAKPTRIICGLQRFGIGFSFAIIYSSLLTKTNRISRIFESARRSARRPSFISPKSQLAIASVLISVQVIGTAVWFLLEPPGTRQYYPDGKRNQVILKCSIRDSSFLLSLIYNMLLITICTVYAVKTRKIPENFNESKFIGFTMYTTCIIWLAFVPIYFGTGNSFEVQITTLCVSTSLSAYVALFCLFSPKVYIIIFHPDKNVRKLTMNSATYKRAPTSSTCGTSVNQGNGKGTTVEQVKLSVHMTPSREQTTETDKDSLASL

>Cuttlefish_mGLuR_A0A812EFN3

MLFTVDHINRNSNILPGIKLGAAIYDTCARATYALEQSLEFVRASISTLDASEFECDDGSLAKAKYIPTAVAGVIGGSSSSVSLQVANLLRLFHIPQISYASTSAALSDKTRFDYFVRTVPPDTFQAIAMVDIVSAFNWTYVSTVASQGEYGESGIDYFQQEARQRNICIATTVKIPTNSKSNTFSYLISRLLEKKNARVVILFVRIEDATRILESATKMNVTNHFVWIASDAWGRQPHPVKRNEAVAEGALTIELTSALIHPFNDYFRQLDPYLNTRNPWFREYWEKVHDCKWMTEERSSLPSLDSIPERICNGDEKMNPGEYKQESKVQFIYDAVYALAIALHRMQKDLCPKSVRLCDQMKKIDGEVLLKKYLLNTSFEDGYGARVEFDEHGDALGRYMIFNYRYNKTKKAYEYKNVGTWSTNGLELSEDDITWSGGISNIPVSRCSRPCNYDEIKSVHDTCCWICIKCQDWEYLYDEYTCKDCGLGRWPNATKRSCFDLELQHMTWDSIYAIVPMSLSCLGIIGTVTVIVTFVRYNDTPVVMASGRELSYMLLSGCLFCYLMSFILIAKPSMFICSVQRIGVGFGFAIIYSALLTKTNRISRIFYNARRSARRPPFISPRSQIAIASILISIQIVFTVIWLVLEPPGTRHYFPYNKRNEVILKCKTRDISFLVSLIYNMLLIIICTVYAVKTRNIPENFNESKFIGFSMYTTCIIWLAFVPIYFGTLNAFQEVNSQWPDEPSRLGTTSAVVDIDDALPVLPRFGAPRHQLSEPSCPLEQAISPAHFTGCSLRILVSAVSTLFHFDFSITLFLVILILIPPQFLSLSLSLSLSLSLSLSLTHALSLVSASALFSLSSSVSLYLSLSFFTIFSLSSFFLSLFLSFFLSLFLSFFFSLSLSYFQPRFRYLEANGMIERFHCSLNQSLKTALIYTSWLQNIPNSLSLSLYLSIYLSISLSLYLPPSRISSFELLKWNFHCEAKRAVLVPPFYLLSSFCRPSFTDPRIFSPCLCLYLYFTSFNLSLSLSLSLSLCLCYDRGGRFLHNTLYLSIYLSIYLSIYLCIYLSIYLSISHYQSRFRFGFLVLLFSMCHSFFFNASCFLLLFVRISLSLSSLCFLALSFSRCLSLSLSIYLSIYLSIYLSISHSNPLSMESIVFPFAFHFFNFVSFSLLFLFSLVFLFHLTTF

>Cloeon_dipterum_mGLuR_A0A8S1C1N6

MRVLVVLLALSGCCLAQLSVKMPGDIVLGGLFPVHEKGEKSPCGAKVYNRGVQRLEAMMFAVDKINADKTLLPTIRLGVNILDTCSRDTYALNRSLEFIRGSLNNLDVSAFECNDRRPPRIRSNSTGPVFGVVGGSYSSVSLQVANLLRLFHIPQISPASTAKALSDKSRFDYFARTVPPDTFQAIALVDIVKSLNWSYVSTVYSEGSYGEYGIEVFHREAQERNVCIAAAEKVPSAADERVFETILGKLLKKQNAKGVVLFTRAEDARGLLNASKRMQTSLHWIASDGWGKQQKLVESLEEVAEGAITVELQSEHMPGFDEYMMSLTPDNNHRNPWFDEYWQDTFSCILPQYMNAEDVDQEICSMDLRLSEKVGYEQESKVQFVVDAVYAFAYALHNLHRDLCRPKDKVCPAMATYDGGDFYRNYLLNVAFKDLGNSEVKFDSQGDGLARYDILNYQKLPNTSGYHYKVVGKWFHSLELNIDELVWNRGSDVIPTSACSLPCAVGMIKMQQGDTCCWICDKCEDYEFVYDEFTCRDCGPGRWPYPDKLSCFDLPLQYMRWDSLFAIVPAAISCLGIILTMGVVILFIRNNDTPIVKASGRELSYLLLGGILLCYLNTFALLVKPTTTSCILQRFGVGGGFSIIYGALLTKTNRISRIFDSAAKSAKRPDFISPRSQLIITCTFIGVQVLFTAVWLLVETPGTRHYYPDRTQVILKCKIHDSSFLISQVYNMLLITICTVYAVKTRKIPENFNESKFIGFTMYTTCIIWLAFIPIYFGTGNSYEIQVTTLCVAISLSASVALVCLYSPKIYIIVFHPDKNVRKLTMNSATYRKAPTSSTSANHAETFL

>Honeybee_mGluR_A0A7M7J1C3

MTPQRWGSTPDCVGAVTVEEAQEGDGTFYYFPSPHIRGLCSPVLAERSPVITVEKDSRLIDIVDAVRGGVDMCFNTNSCISFEKAACSTNNETSERRACNDSAKANCCGMQRTNFPRRLLAQENIALILDGISRFIGESRISSILSSVAQLPANRLNSSLFLQPSLMAAATPFFTMALADRQSIAVMSLSMILALVSAAWRMVGDVLYGCGADRSGIWGVRLLLLAGLLSGEAGRGAQGGAGGGAHALKAHIPGDIILGGLFPIHVKGENAKCGGINKDRGIQRLEAMLFAIDRINEDETLLPGVRLGATILDTCSSDSYALNQSLEFIRASINTADPHNYYCHGGGNLTLKPEASAAISGVIGGSYSEVSLQVANLLRLFRIPQISPASTGTALSDKTRYDFFARTVPPDTFQALAMVAVVETFNWSYVSLVYSEGQYGESGQEQFTKEARHHNICIAISEKIPHSATEQQYLSALERLQQKPNAKVVVLFVRADHAEGVLRAAKELKRKYRDRLQSFDFYWIASDGWGARALSEDMQEAAEGAITIELESRRIEAFDRYMLDLTVENNERNPWFRDYWEALFECKIRNTSSEKEKLPQGISGSNSSLPSCSENLNISSTKDGYKQENKVQFVVDAVYAMAHAIDSVRERVCGAHGVCPRLKEVDGGLIYQALLNVSFVDLVNSTVRFDEKGDAQAPYIIYNYRRNYSSGELQYIQVGKWLGVEEGLELMAGDITFGRRETHDRVIPVSVCSAECGIGEVKKVQAGDHCCWICTRCEPHEFVVNESTCADCGPSRWPHPHKRSCFDLPVQHMQWNNLFSAVPIAVALLGIVLTCGTIAVFVRNNETPIVKASGRELSYMLLSGILICYLMTFVLLLKPTPVSCTAQRFGVGLGFSIIYGSLLTKTNRISRIFDSARRSARRPSFISPKSQMVITCMLISVQVMATLVWFVLEPPATRTEYPEGRRNQVILKCKIRDSSFLVSLVYNMFLITTCTVYAIKTRKIPENFNESKFIGFTMYTTCIIWLAFVPIYFGTGNNFQVQITTLCVSISLSAYVALFCLFSPKVYIILFHPDKNVRKLTMNSATYKKAPTSSTTGGGGGAGGGPPGHNLGSGHGTAVNNQPPTTCTTGTADSCVETVKLVPVATISQHTLQQTSPEMTVAQALRGAPPCSQPTQIEGGRCFQGANTGECVTSDQPRLPGQYSSASRATPSSSPPATGNKSPCDQGSTMMKAVDDRGGGDCDSMQSL

>Daphnia_mGLuR_E9FZM4

MLSTIGSSPSNSPGASIQLPGDIILGGLFPVHVKGEKTPCGSAVYNRGIQRLEAMLFAVDKINREGRLLPGIRLGVNVVDTCSRDTYALNRSLEFIRASLNAMDHSSASFQCRDGSTPRDRRPPSQQSKGFGPVFGVVGGSYSSVSIQVANLLRLFRIPQISPASTAKVLSDKSRFEFFARTVPPDDYQAITLVDLVERFNWTYISTVASEGSYGESGIEVFHREAGARNICIAVAERVSSTADERVFDGIIRNLYRKPFARAIVLFTRADDARGLLAAAKRLSITSHFIWVASDGWGRQHKLVEGLEDVAEGAVTVDLESKSVPGFDDYMLSLTPQNNQRNPWYGDYWQEVHGCLLPHNLQPQSDSSAAASSVEQQQSTLQQPGNISICPAGLRLTHLGYEQDSKIQFVVDAVYAFAHAISALQRDVCSAGQRHHPPVYGACPQLLSYDGGDFYTKYLLNVSFLDPAGSEVKFDARGDGLARYTIMNYRRLPNSANNGYDYKEVGKWYNELELDPAEVMWTREQSAIPSSVCSQPCGVGQVKITQQGDTCCWSCDRCDPWEYVENEFKCADCGPGRWPYDDKHGCFDLDMQYMRWDSLLAIVPVCVSCGGILLTVTVISIFIRHSETPIVKASGRELSFVLLGGILLCYFNTFTLLAKPMVVTCAIQRLSVGTGFSIVYGALFTKTNRISRIFDSASRSAKRPSFISPKSQMVITWCIISFQMLGTVLWMWMEPPGIRAAYPQRDQTILKCRMEDSSFLLSQVFNVLLIAVCTVYAVKTRKIPENFNESKFIGFTMYTTCIIWLAFLPIYFGTANTNEIQITTMCLTISLSATVALVCLYVPKVYIIVFHPDKNVRKLTMTATYRKAPVRQGTATTPTANNKNHHPGKNI

>Octpus_mGluR_A0A6P7SXY7

MNSRRKLRRLYFSLVLTILVWVSFAFSVAPDNKTGKALIPGDIILGGLFPVHQKGEGDKPCGEIDVQRAIQRLEAMLFTVDHINQNASILPGIKLGAAIHDTCARATYALEQSLEFVRASISTLDASEFECEDGSRAKAKYIPTAVAGVIGGSSSSVSLQVANLLRLFHIPQISYASTSAALSDKTRFDYFVRTVPPDTFQAIAMVELVQAFNWTYVSTVASQGDYGESGIDYFQQEARLRNICIATTVKIPTNSKTETFKYIIRRLLEKKNARVVILFVRIEDATSILESATRMNVTNHFVWIASDAWGRQPHPVKNNEKVAEGALTIELTSTVIPQFDDYFQRLDPYLNTRNPWFTEYWEKVHNCKWKTEERSVPLLINEMPRQICNGDETLEPGYYKQEGKVQFIYDAVYAMALAIHRMQQDVCPRTTKLCERMKKIDGEVLLKKYLLNTSFYDGYGALVQFDKQGDALGRYSIFNYAYDNLTGQYAYKHVGTWGKNHLELTGDEITWSGGTDLIPMSRCSRPCNYDEVKSVHDTCCWICIKCEDWEYLYDEYSCRDCGLGRWPNATKRSCFSLELQHMTWNSIYAIVPLSLSCLGIIGTVTVMVTFVRYNDTPVVMASGRELSYMLLSGCLFCYLMSFILIAKPSMFICSVQRIGVGFGFAIIYSALLTKTNRISRIFYNARRSARRPPFISPRSQIAIASILISIQIVFTVIWLVLEPPGTRHYFPYNKRNEVILKCKTRDISFLVSLIYNMLLIIICTVYAVKTRNIPENFNESKFIGFSMYTTCIIWLAFVPIYFGTLNAFQIQITTLCISISLSASVALLCLFVPKMYIILFHPEKNVRKLIMNSATYKRAPTSSATITTATNHGTFTEHIKLNVQNSAQARAVCSPTDIKTDKDSVASL

>Oyster_mGluR_A0A8B8BIF7

MMRVISLLWTSLVENILVILFVFLLRTRGENPALSAYIPGEIIIGGLFPIHQKSSGDRAICGEINLDRGVQRAEAMLFTIDEINKNPKILPKIKLGAKIYDTCARGTYALERSLEFIRGSFTSIDSSDFFCDDGTKARANSTFENVAGVIGGSYSSVSIQVANLLRLFKLPQISYASTSADLSDKARYDYFSRTVPPDNFQAKAIVDIVEYLNWTYVSTVASEGDYGQSGIDDFKEKAVARNICIAESIKILSNSNPSTFDDAITKLLAKENAKIVVLFLRIEDATQLLDAARRSGVGSRFVWIASDAWGTQEKPVLQNAGVAKGALTIELQSTFIPAFHDYFMSLTPQNNVRNPWFREFWETAHNCSFQLGPKEPPDKARCTGNEQLSPVTFKQETKVQFIYDAVYAMALAIDAMHNASCKTEELCDDMRQVNGMILRDFILNTTFDDGYGARVKFDKKGDAMGRYNIMNFQWNETSRSYQYAVVGSWTDSLTIDTDKIVWAGQNREVPSSRCSRPCEFNEFKFVGKDGDTCCWACIKCQEWEYLKDEFTCEDCGVAMWPNADKRGCHRLPEQHINLVSVYALVPVILSCLGLIVTCIVIATFLKHNDTPVVMASGRELSYMLLGGCVLCYLVTFVLIARPSPITCAIQRAGIGLGFSVIYSSLLTKTNRISRIFDSARRSARRPPFISPKSQIVITLVLISIQVLFSIVWLVLERPGTRLYTPKNKRNEVILKCKTDDISFLVSLLYNIILIIVCTLYAIKTRKIPENFNESKFIGFAMYTTCIIWLAFVPIYFGTLNSFEVQITTLCISISLSATVALVCLFTPKMYIIVFQPEKNVRRLTMNSASYKKPVTNSSSVLATNNHDSYTDERIKLNVNYDEKHGVRTVPVPVSKTNGDQDSLHSL

>Drosophila_mGluR_A0A6J2TXB7

MWQPFRPFRSADLGGIQICMLLLLSAFWWSSLPLLEGSGASQAPQDTVSVSLPGDIILGGLFPVHEKGEGAPCGPKVYNRGVQRLEAMLYAIDRVNNDSNLLPGITIGVHILDTCSRDTYALNQSLQFVRASLNNLDNSAFVCSDESSPQLRKNATSGPVFGVIGGSYSSVSLQVANLLRLFHIPQISPASTAKTLSDKSRFDLFARTVPPDTFQSVALVDIIKNFNWSYVSTIHSEGSYGEYGIEAFHKEASERHVCIAAAEKVPSAADDKVFDAIISKLQKKPNARGVVLFTRAEDARRILQAAKRANLAQPFHWVASDGWGKQQKLLDGLEEIAEGAITVELQSEIIEDFDRYMMQLNPRSNLRNPWFAEYWEDTFNCVLLDSSELINPINRSDPKQLEDGRVICDENFRLSEKVGYEQESKTQFVVDAVYAFAHALHNLHNDLCLQSQACPAMASYDGKEFYNNYLLNVSFIDLAGSEVKFDRQGDGLARYDILNYQRLENSSGYQYKVVGKWFNNLELNLPTVVWNKEVDLPTSACSLPCEAGMIKKQQGDTCCWICDNCEPYEYVYDEFTCKDCGPGYWPYPDKLSCFALDIQYMRWNSLFAIVAVSLAVVGIFGTFIVILLFAKNHDTPLVRASGRELSYTLLFGILVCYCNTFALIAKPTIGSCVLQRFGIGVGFSIIYSALLTKTNRISRIFHSASKSAQRLKYISPQSQVIITTSLIAIQVLITMIWMIVEPPGTRFYYPDRTEVILKCKIQDMSFLFSQLYNMILITICTVYAIKTRKIPENFNESKFIGFTMYTTCIIWLAFVPIYFGTGNSYEIQITTLCISISLSASVALVCLYSPKVYILVFHPDKNVRKLTMNSTVYRRSAATAAVQGGMLSSSGYSRTPAPGTAVGATTATSTDRGNSQNGSQRSPSLVLVQSQAAVVHNHEEFNGAPATAECDSCRAEIPEPQCAAIEDPTIPSTIGKCSQD

>Sea_urchin_mGluR_A0A7M7N083

MGPITFKRSPAFSGTKMISSAHTMLLILTLLLCLATCAKLGSSVKSGITAKVIGDLMIGGLFPIHEKGSQNVGSLDDERCGLINEDRGIQRVEAMLFAIDKINNDSTILPGIRLGASLRDTCSIDTFALEQSLEFVRVSLTTSNPADCLNKSTIVASTSAGSVAGVIGGSYSTVSMQVANLLRLFEIPQISYASTSARLSDKSRFEFFARTVPPDTLQAKAIADILSHFNWTYVSTVWSSGEYGESGMDQFEKERLHRNICIAASEKIPYSPDAMTYDIIISRLIKKKAAKVVVLFTRDVDARDLLEAALRVNASKKFIWVASDGWGVQDTPVNGRETVAEGAITIELQTKKIKGFDQYFRGINPRTNTRNPWFKDFWEQKFQCLLEPGDSVNTSTPCAENVLDEANHAQEKKTQFVVDAVFAMAHALHTMQTDMCPHTEELCDNMLPIDGRKLFKEYILNTTFVDLADTEVSFDSQGDGLGRYDVMNYRPIEGTDDYRYVKVGEWANILSMNLSAVHFHPELGDYNSHNVPYSQCSMPCGPGQVKLIQDDQICCWMCIKCQPWEYLQNEYTCRPCDIGYWPTPDLTTCYKLAKQHMDWVSVYTIVPMCFSLAGIIVTCGVIIIFFQYQDTPLVKASSRELSFLLLGGILMCYSMTIPIVTKPSVIVCSIQRLGLGLSFSICYAAMLTKTSRIARIFDSASRSAQRPKYISPCSQLVICLGLISVQIFGELVWLAVDPPSTVTRFAEDRSEAILKCGITEISLVLSLVYDMFLIAMCTVYAFKTRKIPENFNEAKFISFTMYTTCIVWFAFVPVYFVTKDDFRLQTTTLCVSVSICASVALACLFAPKVYIIVFQPHKNVRRLSSHSSRRSQYDGTRFRDEVPSKDLDRMASN

>Starfish_mGluR_A0A8B7YZS6

MEQILNCICLLMVSFELMCYAVVAVYPDYLGSVGEGKRLRVDGNIVLGGLFPIHEKGKNGSICGVINEDRGIQRLEAMLFAIDLINNDTSLLPGITLGAEIRDTCSEDTYALEQSLEFVRASLTTVSTSSSPECPAEHINKTGQHGVVAGVIGGSYSTVSIQVANLLRLFQIPQISYASTSSELTDRKRFEYFARTVPPDTLQAKAIADIIASFGWTYVSTVASDGNYGESGIYEFTKEAGLRNICIAKSEKIRRSADSETYDNIIRGLRRKQNAKVVVLFTRVEDARDLLSAAKRANLSENFIWLASDGWGAQESPVKGNEAVAEGAITIELQTQKIDKFDEYFLNLNPKTNKRNPWFKEYWEQKFKCELPDDPGEGVCARESLDDGTHLQEKKTQFVVDAVYALVKALDNMHRVVCNETSSLCDGMIPINGERLFKEFVLKVVFTDIVDQQVSFDENGNGLGRYDILNFRRDENQENHYHYVKVGKWYGQLEMDRHAVQFHPSVSLRSETNLPMSQCSLPCEAGEIKRLQDDDEICCWLCTACRPYQYVENEHRCRDCEKGYWPIKNLSGCYKLREEYMELDTLWCIVPMAFSLFGIIATCAVAVVFITHNQTPLVKASSRELSYILLLGFAACYAMTFPILMKPSVATCSIQRVGLGLSFCMCYSALLTKTNRIARIFDSASRSAQRPKYISPKSQLVICFGLVSVQLFGELVWLVVKPPGTGVHITEARNHAVLKCNISDVSLVVSLAYNMLLILCCTVYAFKTRKIPENFNEAKFIGFTMYTTCIVWLAFVPLYFGTTWDFRMQTTTLCVTVSMSATVALACLFTPKVYIIVFQPEKNVRRLNTTSTRRSHYEAPPRDVMNNHSHCGSRGKYTMRFNNQTTQYDSQDTEAETSQAFL

>Medaka_mGluR3_H2M092

MVSCIPALVLVMMCQSTLLSDPPPSRREIRIEGDLVLGGLFPVHEKGAGMEECGRVNEDRGIQRLEAMLFAIDRINTDNSLLPGVTLGVHILDTCSKDTYALEQALEFVRASLTKVDDTEFICPDGSYALQEDSPLAIAGVIGGSFSSVSIQVANLLRLFQIPQISYASTSAKLSDKTRYDYFSRTVPPDFYQAKAMAEILRFFNWTYVSTVASEGDYGETGIEAFEQEARMRNICIATSEKVGRSNAKKSYEAVIRQLLQKPNARVAVLFLRSDDARELLAAAARLNTSFIWVASDGWGAQESIVKGNEVTAEGAITLELVANPLPEFNRYFLSLNPVRNHRNPWYKEFWEQRFQCSLGGSSGHGETSHMPPCDQNLSIDKNHFEPESKIMFVVNAVYAMAHALHNMQRSLCFNTSKLCDGMKPLDGRKLYRDYILNVSFPAPFSPSGSETVVKFDTQGDGMGRYNIFSYQRSSERYGYVPVGEWAETLTLNSDLIHWPREVVPTSQCSDPCERNEMKKMQAGEYCCWICTACEAHEYLADEFTCAPCAPGQWPTDDLTSCYDLPEDYIMWEDAWAIGPITIACVGFMCTGLVIWVFIRHNNTPLVKASGRELCYILLSGVFMSYAMTFLFLAKPSPAICALRRLGLGTSFAVCYSALLTKTNRIARIFNGVKEGAGAVRPRFISPFSQVFICLSLISVQLVMVTVWLLLEVPGTRRFTLPERRQTVILKCNVRDSSMLLSLGYDVLLVILCTVYAFKTRKCPENFNEAKFIGFTMYTTCIIWLAFLPIFYVTSSDYRVQTTTMCISVSLSGFVVLGCMFAPKVHIIMFQPQKNVTSHRLNLNRFSVSGAAATYASHASVSAHFVPAVCNGREIVDSTTSSL

>Salmon_mGluR3_A0A8C7FAD2

MLSRITTVLLAVLCRCVLVSLGVDPLHPRREIRIEGDLVLGGLFPVHEKGGGIEECGRVNEDRGIQRLEAMLFAIDRINSDPTLLPGVSLGVHILDTCSRDTYALEQALEFVRASLTKVDDTEFICPDGSYALQDDSPLAIAGVIGGSYSSVSIQVANLLRLFQIPQISYASTSAKLSDKTRYDYFARTVPPDFYQAKAMAEILRLFNWTYVSTVASEGDYGETGIEAFEQEARMRNICIATSEKVGRSNAKKSYEAVIRQLLQKPNARVAVLFLRSDDARELLSAAARLNTTFIWVASDGWGAQESIVKGNEATADGAITLELAANPVPAFNRYFLSLDPINNRRNPWYREFWEQKFQCSLGGGGGGAGGVGSGGMVSLSLPPCEKGVSVDNSNFEPESKIMFVVNAVYAMAHALHKMQRTLCSNTTKLCDSMKALDGRKLYRDYLLNISFTAPFSPLGSETVVKFDPYGDGMGRYNIFSYQRNADRYGYVPIGEWAETLSLSSDLIRWPREVVPPSQCSDPCARNEMKKMQAGEYCCWICTACEAHEYLADEFTCSPCAPGQWPSEDLTFCYDLPEDYIMWEDAWAIGPISIAIVGFLCTSLVFWVFVRHNNTPLVKASGRELCYILLLGVFMSYSLTFLFLAKPSPTICAMRRLGLSTSFAVCYSALLTKTNRIARIFSGVKDGGGTTRPRFISPTSQVFICLSLISVQLVMVSVWLLLEVPGTRRFTLPERRQTVILKCNVRDSSMLLSLVYDVLLVILCTVYAFKTRKCPENFNEAKFIGFTMYTTCIIWLAFLPIFYVTSSDYRVQTTTMCISVSLSGFVVLGCMFAPKVHIIMFQPQKNVTSLRLNINRFSVSGPATTCASHVSAQYVPTVCNGREIVDSTTSSL

>Human_mGluR3_Q14832

MKMLTRLQVLTLALFSKGFLLSLGDHNFLRREIKIEGDLVLGGLFPINEKGTGTEECGRINEDRGIQRLEAMLFAIDEINKDDYLLPGVKLGVHILDTCSRDTYALEQSLEFVRASLTKVDEAEYMCPDGSYAIQENIPLLIAGVIGGSYSSVSIQVANLLRLFQIPQISYASTSAKLSDKSRYDYFARTVPPDFYQAKAMAEILRFFNWTYVSTVASEGDYGETGIEAFEQEARLRNICIATAEKVGRSNIRKSYDSVIRELLQKPNARVVVLFMRSDDSRELIAAASRANASFTWVASDGWGAQESIIKGSEHVAYGAITLELASQPVRQFDRYFQSLNPYNNHRNPWFRDFWEQKFQCSLQNKRNHRRVCDKHLAIDSSNYEQESKIMFVVNAVYAMAHALHKMQRTLCPNTTKLCDAMKILDGKKLYKDYLLKINFTAPFNPNKDADSIVKFDTFGDGMGRYNVFNFQNVGGKYSYLKVGHWAETLSLDVNSIHWSRNSVPTSQCSDPCAPNEMKNMQPGDVCCWICIPCEPYEYLADEFTCMDCGSGQWPTADLTGCYDLPEDYIRWEDAWAIGPVTIACLGFMCTCMVVTVFIKHNNTPLVKASGRELCYILLFGVGLSYCMTFFFIAKPSPVICALRRLGLGSSFAICYSALLTKTNCIARIFDGVKNGAQRPKFISPSSQVFICLGLILVQIVMVSVWLILEAPGTRRYTLAEKRETVILKCNVKDSSMLISLTYDVILVILCTVYAFKTRKCPENFNEAKFIGFTMYTTCIIWLAFLPIFYVTSSDYRVQTTTMCISVSLSGFVVLGCLFAPKVHIILFQPQKNVVTHRLHLNRFSVSGTGTTYSQSSASTYVPTVCNGREVLDSTTSSL

>Mouse_mGluR3_Q9QYS2

MKMLTRLQVLMLALFSKGFLVSLGDHNFMRREIKIEGDLVLGGLFPINEKGTGTEECGRINEDRGIQRLEAMLFAIDEINKDNYLLPGVKLGVHILDTCSRDTYALEQSLEFVRASLTKVDEAEYMCPDGSYAIQENIPLLIAGVIGGSYSSVSIQVANLLRLFQIPQISYASTSAKLSDKSRYDYFARTVPPDFYQAKAMAEILRYFNWTYVSTVASEGDYGETGIEAFEQEARLRNICIATAEKVGRSNIRKSYDSVIRELLQKPNARVVVLFMRSDDSRELIAAASRVNASFTWVASDGWGAQESIVKGSEHVAYGAITLELASHPVRQFDRYFQSLNPYNNHRNPWFRDFWEQKFQCSLQNKRNHRQICDKHLAIDSSNYEQESKIMFVVNAVYAMAHALHKMQRTLCPNTTKLCDAMKILDGKKLYKDYLLKINFTAPFNPNKGADSIVKFDTYGDGMGRYNVFNFQHIGGKYSYLKVGHWAETLYLDVDSIHWSRNSVPTSQCSDPCAPNEMKNMQPGDVCCWICIPCEPYEYLVDEFTCMDCGPGQWPTADLSGCYNLPEDYIRWEDAWAIGPVTIACLGFMCTCIVITVFIKHNNTPLVKASGRELCYILLFGVSLSYCMTFFFIAKPSPVICALRRLGLGTSFAICYSALLTKTNCIARIFDGVKNGAQRPKFISPSSQVFICLGLILVQIVMVSVWLILETPGTRRYTLPEKRETVILKCNVKDSSMLISLTYDVVLVILCTVYAFKTRKCPENFNEAKFIGFTMYTTCIIWLAFLPIFYVTSSDYRVQTTTMCISVSLSGFVVLGCLFAPKVHIVLFQPQKNVVTHRLHLNRFSVSGTATTYSQSSASTYVPTVCNGREVLDSTTSSL

>Biomphalaria_glabrata_ZIP9B_XP_055878950

MDDIWTMLALSLAMLIGCYLAGVIPLTISLSEEKLKLVTVLGAGLLVGTALAVIIPEGVHAMYSSLEGAHEHVHEHAREVNSNQALNIPEEKDIHKEKPVHEDAAPAPEHSHENKLEAHSVIGITLVSGFIFMLLVDQIGGNMHAHSSPSEVEAAGHGVNRNKITATLGLVVHAAADGIALGAAITLSQTHITMIVFVAIMLHKAPAAFGLVSFLLHEGLDRLRIRKHLAIFSAAAPLLTIVTYLVLSLQSKENLADIQTTGIAMLFSAGTFLYVATVHVLPEISVSQTQHKSADGTVIIREQKGFKMTELIALVLGSVIPVVLALGHKH

>Aplysia_ZIP9_XP_005097024

MDEAVTLLGLSFAMLVGCYLAGVIPLTITLSEEKLKMVTVLGAGLLVGTALAVIIPEGVHAMYSNGKDHEGHSHQLKEIEHKENEVVNAVGSLAGNDDAATADLHRDHHHDMGEEHSKIGISLVTGFIFMLLVDQIGGNMHSHSVSNDAEATGTVQNRNKITATLGLVVHAAADGIALGAAITLSENHITMIVFAAIMLHKAPAAFGLVSFLLHEGLDRARIRKHLAVFSASAPLLAIITYLSLSQQNKETLSDMHTTGIAMLFSAGTFLYVATVHVLPEISTSHTQHKAPDGTVIVREQKGFKKSELAALVVGSLLPVVLAMGHKH

>Patella_vulgata_ZIP9_XP_050405555

MDDITTLVLLSLGMSIGCYLSGLIPLAFTLSEEKLKLVTVLGAGLLVGTALSVIIPEGVHAMYSSGRRHGASYHHVEKLAPETIRRNANIKLIHSNVKEDGLEDSHHDMSEEHSRIGISLVAGFIFMLLVDQLGGSHFHAASDLESGSEALAHNRNKITATVGLVVHAAADGVALGAAITLAENHITMIVFIAIMLHKSPAAFGLVSFLLHAGFERSRIRKHLLIFSLAAPLLSFVTYLCLSKQSKETLYDMHTTGIAMLFSAGTFLYVATVHVLPEISTGKSLHTSVDGTIIVREHKGFKKIELLALVTGSVIPVVLSVGHKH

>Human_ZIP9_NP_060845

MDDFISISLLSLAMLVGCYVAGIIPLAVNFSEERLKLVTVLGAGLLCGTALAVIVPEGVHALYEDILEGKHHQASETHNVIASDKAAEKSVVHEHEHSHDHTQLHAYIGVSLVLGFVFMLLVDQIGNSHVHSTDDPEAARSSNSKITTTLGLVVHAAADGVALGAAASTSQTSVQLIVFVAIMLHKAPAAFGLVSFLMHAGLERNRIRKHLLVFALAAPVMSMVTYLGLSKSSKEALSEVNATGVAMLFSAGTFLYVATVHVLPEVGGIGHSHKPDATGGRGLSRLEVAALVLGCLIPLILSVGHQH

>Mouse_ZIP9_Q8BFU1

MDDFLSISLLSVAMLVGCYVAGIIPLAVNFSEERLKLVTVLGAGLLCGTALAVIVPEGVHALYEEVLEGKHHQTSEMKQNGIASDKAAEISSVHEHEHSHDHTQLHAYIGVSLVLGFVFMLLVDQIGSSHVHSSDDPETARPSSSKITTTLGLVVHAAADGVALGAAASTSQTSVQLIVFVAIMLHKAPAAFGLVSFLMHAGLERNRIRKHLLVFALAAPAMSMLTYLGLSKSSKEALSEVNATGVAMLFSAGTFLYVATVHVLPEVGGMGHSHKPDTTGGRGLSRLEVAALVLGCLIPLILSIGHQH

>Chicken_ZIP9_NP_001007934.1

MDDFRSICLLSLAMLVACYVAGIIPLAVNFSEERLKLVTVLGAGLLCGTALAVIVPEGVHALYEDILEGKHHPANEMQHVMESEKVAEIAVVHEYGHDHSRLHAYIGVSLVLGFVFMLLVDQIGSSHVHSTDDPEAARSGNSKITTTLGLVVHAAADGVALGAAASTSQTSVQLIVFVAIMLHKAPAAFGLVSFLMHAGLERNRIRKHLLVFALAAPVMSMVTYLGLSKSSKEALSEVNATGVAMLFSAGTFLYVATVHVLPEVGGIAHSHRPESTGGKGLSRLEVAALVLGCLIPLVLSIGHHH

>Zebrafish_ZIP9_NP_001013558.1

MDDFSSISLLSLSMLIGCYVAGTIPLAVNFSEEKLKLVTVLGAGLLCGTALAVIIPEGVHALYEEMLEGVHNHGHGQVEAEVSEQKVAEGVVRPSGEHGHGHEQLHAYIGISLVLGFVFMLLVDQIGSAHMHSSDDPEAARAASSKITTTLGLVVHAAADGVALGAAASTSQTSVQLIVFVAIMLHKAPAAFGLVSFLMHAGLERNRIRKHLLVFALAAPVLAMVTYVGLSQSSKEALSDVNATGVAMLFSAGTFLYVATVHVLPEVGGMGGHSHSPGGSAGKGLSKLEVGALVLGCLIPLVLSIGHQH

>Amphioxus_ZIP9_A0A9J7M0P3

MDMWIGDVWGITLLSLAMLVGCYVAGSIPLAISLSEQRLKLVTVMGAGLLVGTALAVIIPEGVHSLYSAQTEKHHAKMMKSVGPRAVKVVNTSKDFHFHMHEHHDEHEELHQYIGVSLVLGFVFMLLVDQLGGAHGHSHTTDVEGSTPTKQNRNKITATLGLVVHAAADGVALGAAASTARADVELIVFIAIMLHKAPAAFGLVSFLMHEGFDRNRIRKHLLIFSLAAPLLTIITYFGLSQSGKEALSSVNATGITMLFSAGTFLYVATVHVLPEIQSMGTQGGGEGGSDGGHHHGFSKLELVAFVTGALFPLLLSIGHKH

>Frog_ZIP9_Q6NRM1

MDDFTSISLLSLAMLVGCYVSGIIPLAVNFSEEKLKLVTVLGAGLLCGTALAVIVPEGVHALYEEALEAKHHEMGEIHKVKDAETGAEASVAHEHDHSNLHAYIGVSLVLGFVFMLLVDQIGSSHMHSADDPEAARAASSKITTTLGLVVHAAADGVALGAAASTSQTSVQLIVFVAIMLHKAPAAFGLVSFLMHAGLERNRIRKHLLVFALAAPLLSMLTYLGLSKSSKEALSEVNATGVAMLFSAGTFLYVATVHVLPEVGGMGHSHKQDLGAAKGLSRLEVCALVLGCLIPLVLSIGHQH

>Salmon_ZIP9_A0A1S3PYQ3

MSIDDFTSISLLSLAMLVGCYVAGTIPLAVNFSEEKLKLVTVLGAGLLCGTALAVIIPEGVHALYEEVLEGAHHAPGQVKGVEVSEPKEGVDAALGVSGEHSHSHEHLHAYIGVSLVLGFVFMLLVDQIGSAHVHSSADDPESARVASSKITTTLGLVVHAAADGVALGAAASTSQTSVQLIVFVAIMLHKAPAAFGLVSFLMHAGLERNRIRKHLLVFALAAPVLAMVTFVGLSQSSKEALLDVNATGVAMLFSAGTFLYVATVHVLPEVGGTGHSHAPVQGKEAAKGLSKVEVGALVLGCLIPLVLSVGHQH

>Human_NPFF2_AF268899

MNEKWDTNSSENWHPIWNVNDTKHHLYSDINITYVNYYLHQPQVAAIFIISYFLIFFLCMMGNTVVCFIVMRNKHMHTVTNLFILNLAISDLLVGIFCMPITLLDNIIAGWPFGNTMCKISGLVQGISVAASVFTLVAIAVDRFQCVVYPFKPKLTIKTAFVIIMIIWVLAITIMSPSAVMLHVQEEKYYRVRLNSQNKTSPVYWCREDWPNQEMRKIYTTVLFANIYLAPLSLIVIMYGRIGISLFRAAVPHTGRKNQEQWHVVSRKKQKIIKMLLIVALLFILSWLPLWTLMMLSDYADLSPNELQIINIYIYPFAHWLAFGNSSVNPIIYGFFNENFRRGFQEAFQLQLCQKRAKPMEAYALKAKSHVLINTSNQLVQESTFQNPHGETLLYRKSAEKPQQELVMEELKETTNSSEI

**Supplementary Figure 6.** Transmembrane domain prediction made by the DeepTMHMM tool**
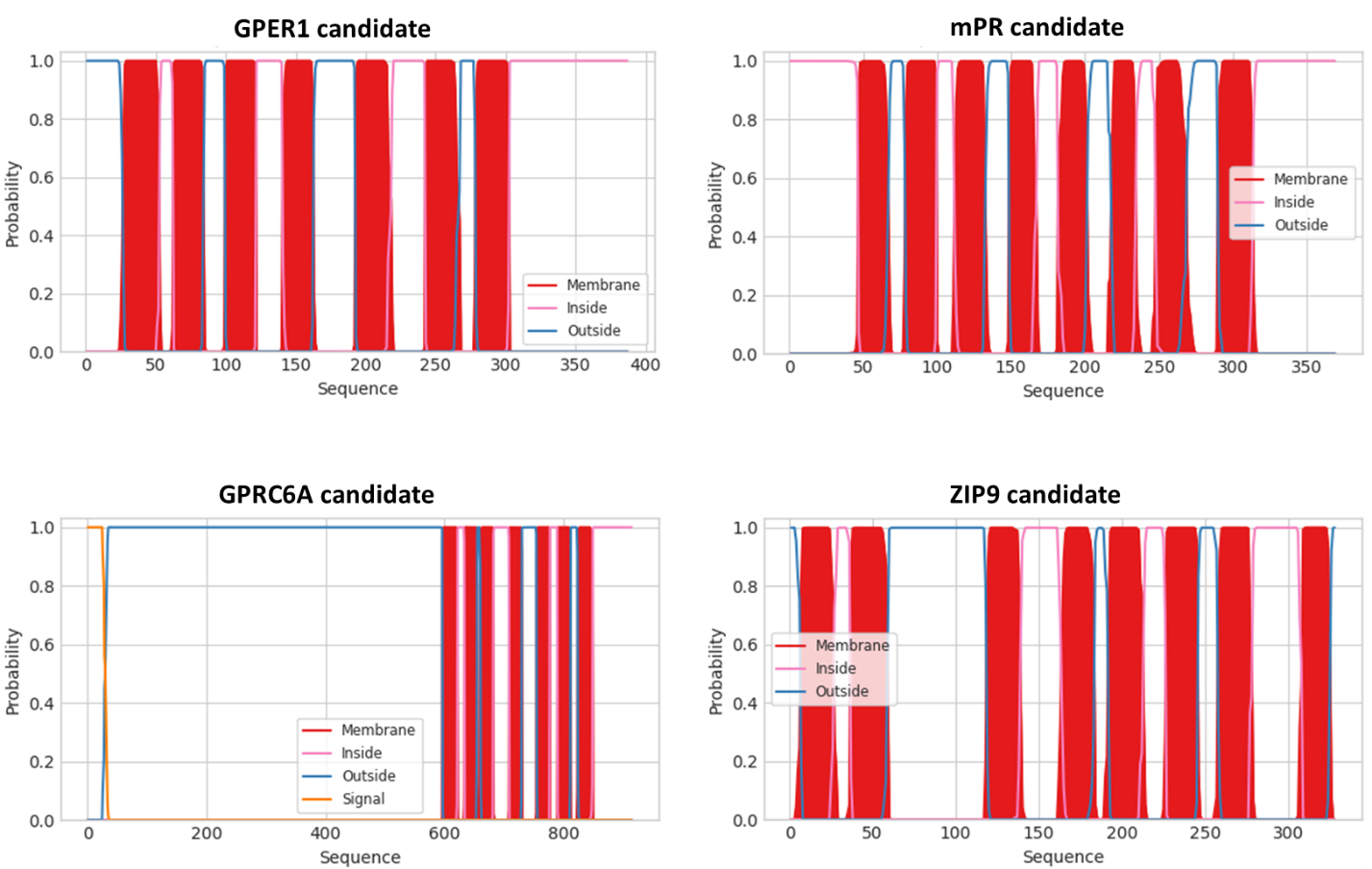
**

**Supplementary Figure 7.** Positive control experiments. **A**: expression of GFP in HEK293 cells; **B**: Expression of *Ciona* GALPR in HEK293 cells; **C**: Dose-response curve of the positive control: calcium mobilization induced by *Ciona* GALP in *Ciona* GALPR-expressing HEK293 cells. Data points are means ± SEM of two independent transfections. Bars = 100 µm (A) and 50 µm (B)

**
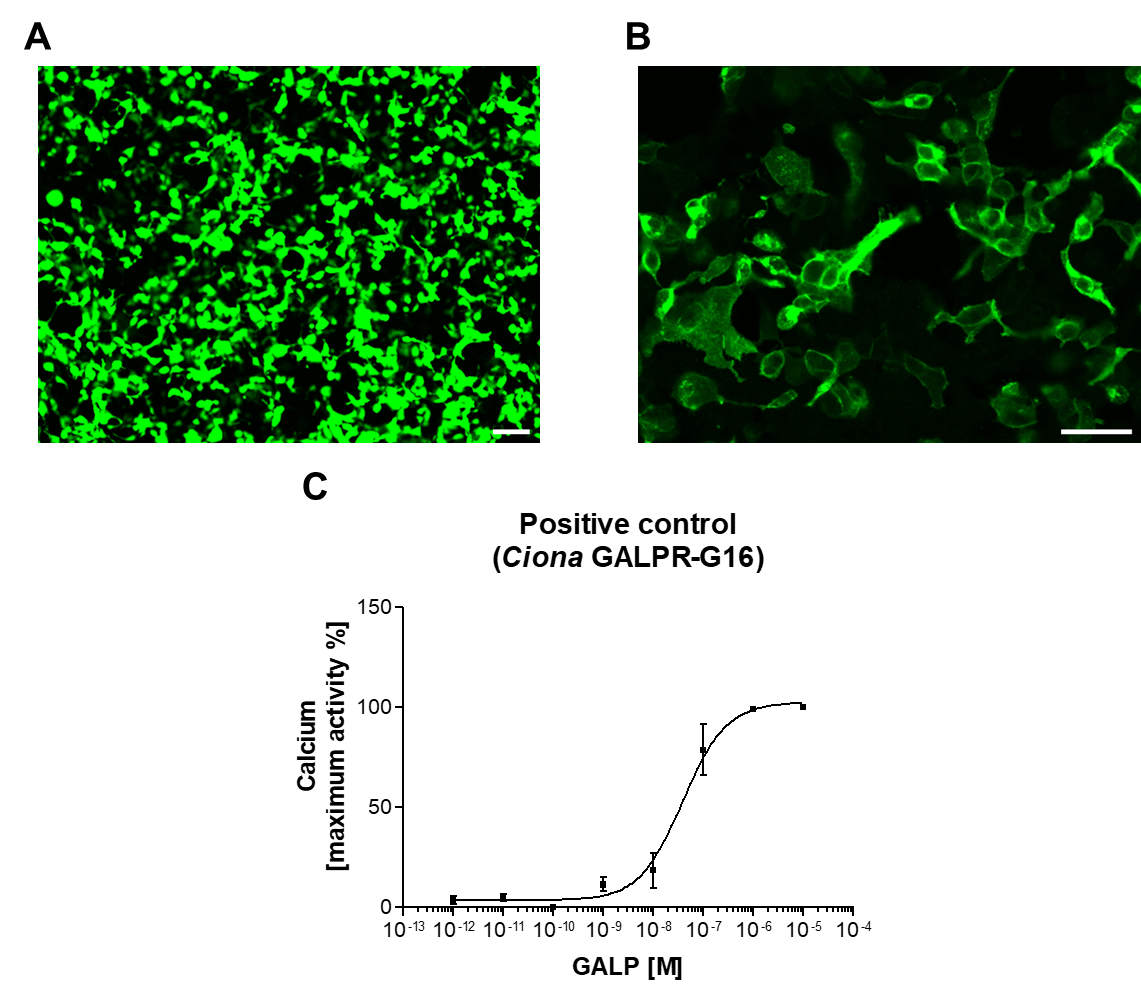
**

**Supplementary Figure 8. Codon usage optimized sequences**

mPR_original ATGTTGTTACTGCCAGCAACATTGAGTAGGGAAGAAATCCCAATACTTTTTCATGAACCC

mPR_optimized ATGCTGTTGTTGCCCGCCACATTAAGCAGGGAGGAGATTCCTATACTCTTCCATGAGCCA

*** ****. ****.**.*****.** *****.**.** **:***** ** *****.**.

mPR_original CATGTTATGAAAGGGTTTAGGCCACTGCACTACCCATGGATGTCTTATTTTTTAAGTTTG

mPR_optimized CATGTCATGAAAGGTTTTAGACCACTACACTACCCCTGGATGAGCTATTTCCTGAGCCTG

***** ******** *****.*****.********.******: ***** *.** **

mPR_original TTTCAGTGGCATAATGAACTCTTGAACATCTGGACACACCTACTGGCATTAATCATGGTG

mPR_optimized TTCCAGTGGCATAACGAACTACTTAATATCTGGACCCACCTCCTGGCCCTCATAATGGTG

** *********** *****. * ** ********.*****.*****. *.**.******

mPR_original TTGGTGAGGGCTAGTATGTGGTGGACAGAGTTTGACCTGCTCCGAGACCCTTACATGTGG

mPR_optimized CTGGTAAGAGCATCAATGTGGTGGACAGAATTTGATTTGTTGCGGGACCCCTACATGTGG

****.**.**:: :**************.***** ** * **.***** *********

mPR_original CCACTGTCAGTGGGGATCATCACTATGATTATTCTCTATGTCTGCAGCTCTGGTGCTCAT

mPR_optimized CCTCTGTCTGTTGGTATTATCACCATGATCATCCTCTACGTGTGCAGCTCCGGCGCTCAT

**:*****:** ** ** ***** ***** ** ***** ** ******** ** ******

mPR_original TGTTTTCAGAACAGGTCAGAATTGGTCCACTACACCTGTTTCATGTTTGACTATGCTGGT

mPR_optimized TGTTTTCAAAACCGCAGCGAGCTGGTGCATTACACTTGTTTCATGTTCGACTACGCCGGG

********.***.* : .**. **** ** ***** *********** ***** ** **

mPR_original ATAGGACTCTATGGTTTTGGAAGTACCATGCTACACTACTGGTATTGTCTCCATGAAAGT

mPR_optimized ATAGGGCTGTATGGTTTTGGCTCTACTATGCTGCACTACTGGTATTGTCTGCACGAGAGT

*****.** ***********.: *** *****.***************** ** **.***

mPR_original TTCATGGGCTCTTTATCCCACCAGCTCGCTATACCAGTTGGAGCAATTTTAGCAGTCTTG

mPR_optimized TTCATGGGGAGCCTGAGTCATCAACTCGCTATTCCAGTGGGAGCTATATTGGCCGTCCTC

******** : *.: ** **.********:***** *****:**:**.**.*** *

mPR_original GTCTGTATCTGCTGCTCTATTTCCAAAACTAAGTACAAGCGCCCTTACCCTTTCACTCGT

mPR_optimized GTCTGCATTTGCTGCTCTATTTCCAAAACGAAGTACAAGCGGCCCTATCCTTTTACACGC

***** ** ******************** *********** ** ** ***** **:**

mPR_original CGTATCTGGCAGATGTCCTCAGTGGCCTCTATTTACATTTGGCTTATATTCCCAATTTGG

mPR_optimized AGGATATGGCAGATGTCTTCCGTGGCCTCAATTTATATCTGGCTCATTTTCCCCATCTGG

.* **.*********** **.********:***** ** ***** **:*****.** ***

mPR_original TACAGAATTTGGTTGTATGTCCACACTGGAGAGTGGGATCCCAGTTTTAAGCACCATATT

mPR_optimized TACAGGATTTGGCTTTATGTCCACACCGGGGAATGGGACCCTAGTTTCAAACACCACATC

*****.****** * *********** **.**.***** ** ***** **.***** **

mPR_original CGTCAGATGTGTTGGTTCACTTTGGGAGGATTTTTCTTTGGTTCAGACATTCCTCAAAGG

mPR_optimized AGGCAGATGTGCTGGTTTACTCTTGGCGGATTCTTCTTTGGCTCCGACATTCCACAGCGT

.* ******** ***** *** * **.***** ******** **.********:**..*

mPR_original TTCTTTCCGGGCACTTTTGATATTATCGGTCACAGCCATCAGCTGTTCCACATATGTATA

mPR_optimized TTCTTTCCTGGGACATTTGATATCATCGGCCACTCACACCAGTTATTTCACATTTGTATC

******** ** **:******** ***** ***: .** *** *.** *****:*****.

mPR_original TTCATGACCACCTATGAACAGCTGAGTGCCCTGTACTTGGAGTTGACCGGCATCAGTGTC

mPR_optimized TTCATGACCACCTACGAGCAGCTCTCAGCACTGTATCTGGAACTCACTGGAATTTCTGTG

************** **.***** : :**.***** ****. * ** **.** : ***

mPR_original ATCATCCACAACATGGAGTCCCCAACTCTCTTCAACACCTGGGGAGTTCTCATCCTAGTT

mPR_optimized ATCATCCACAACATGGAGTCCCCGACCCTGTTTAACACATGGGGAGTTCTTATCCTGGTG

***********************.** ** ** *****.*********** *****.**

mPR_original GTTGTTTGCAATTCCATCGTGGTTTATTTATTTCACATCTCCGTAGAGAGGAAGCTTGAA

mPR_optimized GTGGTTTGTAATAGCATCGTGGTGTACCTGTTCCACATCAGTGTGGAAAGAAAGCTGGAA

** ***** ***: ********* ** *.** ******: **.**.**.***** ***

mPR_original GATGAGAGGAAGAAGGAGGATAAGGAAGAGGAGGATAAGGGAGAGGGCGTGTTAAAGGAA

mPR_optimized GATGAGCGAAAGAAGGAGGACAAAGAGGAGGAGGACAAGGGCGAGGGAGTACTTAAAGAA

******.*.*********** **.**.******** *****.*****.**. *:**.***

mPR_original AAAGCTGAACTTAAGAGTAATTCTCCAAACAGGAAAAGTGCCAATGGTGATGGCTGTGAT

mPR_optimized AAGGCTGAATTAAAGTCCAACTCGCCCAATCGGAAATCCGCGAACGGCGATGGCTGCGAT

**.****** *:***: ** ** **.** .*****: ** ** ** ******** ***

mPR_original GTTCATCACAGAAATGGGGTCATCCAT

mPR_optimized GTCCATCATAGAAATGGAGTCATCCAC

** ***** ********.********

ZIP9_original ATGGATGATATCTTGACTCTCCTTTCTCTGTCCATAGCGATGCTTGTTGGCTGTTACCTT

ZIP9_optimized ATGGACGACATCCTGACACTGCTCTCTCTCTCTATTGCTATGCTTGTCGGCTGCTATCTC

***** ** *** ****:** ** ***** ** **:** ******** ***** ** **

ZIP9_original GCAGGAGTCATCCCACTGACTATTTCACTTTCAGAGGAAAAACTTAAATTAGTGACAGTT

ZIP9_optimized GCCGGGGTAATTCCTCTGACAATCTCTTTGTCTGAGGAGAAGCTGAAGCTGGTGACCGTG

**.**.**.** **:*****:** **: * **:*****.**.** **. *.*****.**

ZIP9_original CTTGGTGCTGGATTGTTGGTTGGCACTGCCCTTGCAGTTATTATACCAGAGGGTGTTCAT

ZIP9_optimized CTGGGCGCCGGGCTGCTGGTTGGAACTGCTTTAGCAGTTATCATCCCGGAAGGGGTCCAC

** ** ** **. ** *******.***** *:******** **.**.**.** ** **

ZIP9_original GCAATGTACTCAAGTTATGAAGCCCACGAGCATTCACATGATCATGCAAGAGAAGCTGAG

ZIP9_optimized GCCATGTACTCCAGCTACGAGGCGCATGAGCATAGTCATGATCATGCAAGGGAAGCTGAA

**.********.** ** **.** ** ******: :**************.********.

ZIP9_original CCCATTCAAGCATCTGATGCTGCAAAAAATGATAAACAAGGGAGCTCTGCATTAAAAGAT

ZIP9_optimized CCCATTCAGGCTAGCGACGCTGCAAAGAACGACAAACAGGGCTCATCGGCCTTGAAAGAC

********.**:: ** ********.** ** *****.** : .** **.**.*****

ZIP9_original CCAGCCTCACAGCTTGAGCACCATCATGAGCACAGCAAACCAGACGTGCATTCTATTATA

ZIP9_optimized CCCGCATCTCAGCTGGAGCACCATCATGAGCATAGTAAACCAGATGTGCACTCAATCATC

**.**.**:***** ***************** ** ******** ***** **:** **.

ZIP9_original GGCGTTACCCTAGTTATTGGGTTTATTTTCATGTTGTTGGTGGATCAGATAGGGGGGAGC

ZIP9_optimized GGCGTCACCCTGGTGATCGGGTTCATATTTATGCTCCTGGTGGACCAGATCGGCGGTAGT

***** *****.** ** ***** **:** *** * ******* *****.** ** **

ZIP9_original ATGCATTCCCATGCGATTTCTTCAGACCCAGAGACGGCGAGTCATGGACAGAGTAGAAAT

ZIP9_optimized ATGCACTCCCACGCCATTAGCTCCGACCCCGAGACTGCCTCTCATGGACAATCAAGAAAC

***** ***** ** ***: **.*****.***** ** : *********.: :*****

ZIP9_original AAAATTACAGCCACACTTGGACTTGTTGTCCACGCTGCAGCCGATGGTATAGCCCTTGGA

ZIP9_optimized AAGATCACCGCCACCCTCGGGCTTGTTGTTCACGCAGCTGCAGATGGAATTGCCCTGGGT

**.** **.*****.** **.******** *****:**:**.*****:**:***** **:

ZIP9_original GCCGCCATATCATTATCTGAAACTCACATCACCATGATTGTTTTTATAGCAATCATGTTG

ZIP9_optimized GCAGCTATATCCCTGTCCGAAACACACATCACCATGATCGTGTTCATCGCCATCATGCTG

**.** *****. *.** *****:************** ** ** **.**.****** **

ZIP9_original CATAAGGCTCCTGCAGCTTTTGGTTTGGTTTCCTTTTTAATGCACGAGGGATTGGATCGA

ZIP9_optimized CACAAGGCGCCTGCTGCTTTTGGGCTAGTAAGTTTTCTGATGCACGAGGGCCTGGATCGC

** ***** *****:******** *.**:: *** *.***********. *******.

ZIP9_original ACAAGAATAAGGAAACATTTAGCAGTATTTTCTGCTGCTGCACCACTTTTAACCATTATA

ZIP9_optimized ACAAGAATAAGGAAACACCTGGCCGTGTTCAGCGCCGCTGCACCTTTATTGACCATTATT

***************** *.**.**.** : ** ********: *:**.********:

ZIP9_original ACTTACCTTGGCCTAAGTCAGCAAAGTAAAGAAACATTGTCAGATATGCAAACAACAGGG

ZIP9_optimized ACGTACCTCGGCCTCAGCCAGCAATCAAAAGAGACGCTTTCCGACATGCAGACCACCGGT

** ***** *****.** ******: :*****.**. * **.** *****.**.**.**

ZIP9_original ATTGCAATGTTGTTTAGTGCTGGGACATTTTTATATGTGGCCACTGTTCATGTCCTTCCA

ZIP9_optimized ATTGCGATGTTGTTCTCCGCCGGAACTTTCTTGTATGTCGCCACAGTGCACGTGCTCCCC

*****.******** : ** **.**:** **.***** *****:** ** ** ** **.

ZIP9_original GAAATTTCTGTAAGCCAGACACAGCATAAGGCAGCAGATGGAACTGTAATTATTCGAGAG

ZIP9_optimized GAAATTAGCGTCAGCCAGACTCAGCACAAGGCCGCCGATGGAACTGTGATTATACGGGAA

******: **.********:***** *****.**.***********.*****:**.**.

ZIP9_original CAGAAAGGTTTCAAGAAATTGGAGCTTGCAGCCTTAGTGTTGGGTGCACTTTTACCTGTT

ZIP9_optimized CAGAAGGGCTTTAAGAAGCTTGAGCTCGCCGCCCTTGTGCTAGGAGCATTACTACCAGTC

*****.** ** *****. * ***** **.*** *:*** *.**:*** *: ****:**

ZIP9_original TTTTTAGCTGTGGGTCATAAGCAC

ZIP9_optimized TTCCTGGCGGTGGGCCACAAACAT

** *.** ***** ** **.**

**Supplementary Figure 9.** Multiple sequence alignment of molluscan mPRβ candidates and vertebrate mPRβ sequences. Transmembrane domains are indicated with green color.

Biomphalaria -------------------------------MIFLPPALDKDEIPILFHEPYVLTGFRPL 29

Lymnaea -------------------------------MLLLPATLSREEIPILFHEPHVMKGFRPL 29

Crassostrea -------------------------MKHKGHISLLRPTTTKHDVPVLFQEPHVETGFRHP 35

Sepiella ------------------------------MWHLMKPTVTKEQVPLLFHEPHVLSGFRHL 30

Octopus ------------------------------MVQLMKPTVSKEHVPLLFHEPHVLSGFRHI 30

Zebrafish MSSGVLGRLSTLTLSLQQLGQLPHLSNWLPRLPRRQATVHASEVPSLFREPYILSGYRPV 60

Chicken MMTAILERLSTLSLSGPQLSRLPRLLE--DGFPKMPCTVQEGEVPQLFREPYIHTGYRPT 58

Human MTTAILERLSTLSVSGQQLRRLPKILE--DGLPKMPCTVPETDVPQLFREPYIRTGYRPT 58

Mouse MTTAILERLSTLSMSGQQLRRLPKILE--EGLPKMPCTVPETDVPQLFREPYIHAGYRPT 58

: .:* **:**:: *:*

Biomphalaria HYPWTSYLLSIFQWHNELLNIWTHLLALIMVLIRASVWSTEFSLLSD-PFMWPLSVGIIT 88

Lymnaea HYPWMSYFLSLFQWHNELLNIWTHLLALIMVLVRASMWWTEFDLLRD-PYMWPLSVGIIT 88

Crassostrea HQPWFYYICSIFQKHNECMNVWTHLIGLLLTVSRTIEFSNEYDLIGN-PHMWPLSAGLIT 94

Sepiella NQPWSYYFLSLFQVHNECLNSWTHLIALILIIKKMFTFSMEVDLITD-PYMWPLTSGILS 89

Octopus HQPWTYYFLSLFQIHNECLNSWTHLIAMLLVIKKMMTFSKEFSLITD-PYMWPLTSGILS 89

Zebrafish HQEWRSYFCSLFQCHNELLNVWTHLLAIPAVLLQFSFFAGAWGLTLN-LASLPLFLYVLS 119

Chicken GQDWRYYFLSLFQKHNEVVNVWTHLLAALAVLLRFKAFVEGEQLPLD-AWSLPLLIFVLS 117

Human GHEWRYYFFSLFQKHNEVVNVWTHLLAALAVLLRFWAFAEAEALPWASTHSLPLLLFILS 118

Mouse GHEWRYYFFSLFQKHNEVVNVWTHLLAALAVLLRFWAFVEAGALQWASPHTLPLLLFILS 118

* *: *:** *** :* ****:. : : : * ** :::

Biomphalaria MIILYICSSGAHCLQNRSEVVHYTCFMCDYAGIGLYGFGSTMLHYWYCLHEDLLGSFSHQ 148

Lymnaea MIILYVCSSGAHCFQNRSELVHYTCFMFDYAGIGLYGFGSTMLHYWYCLHESFMGSLSHQ 148

Crassostrea MILMYLCSTCAHCFSHKSELVHYTGFMIDYAGIGIFGLGSTIIHYAYCIHDTMLNSSLKM 154

Sepiella IIILYLCSSLAHCLQSRSELAHYTCFMFDYAGIGLYGTGSIVLHYNYCMLPTYKLGALRE 149

Octopus IVILYLCSSLAHCLQSRSELAHYTCFMFDYAGIGLYGIGSIILHYNYCMLPSFDIGILQS 149

Zebrafish SLTYLSFSVAAHLLQSHSELAHYSLFFVDYVGVAVYQYGCSMGHYFYCSEPEWRHSLVGV 179

Chicken SVTYLTCSLLAHLLQSKSELYHYTFYFVDYVGVSTYQYGSALAHFYYSSDQAWYDKF-WL 176

Human SITYLTCSLLAHLLQSKSELSHYTFYFVDYVGVSVYQYGSALAHFFYSSDQAWYDRF-WL 177

Mouse SITYLTCSLLAHLLQSKSELSHYTFYFVDYVGVSVYQYGSALAHFFYSSDQAWYELF-WI 177

: * ** :. :**: **: :: **.*:. : *. : *: *.

Biomphalaria FAIPVGAVLAVIVCICCTISKTKYKRPYPFIRRVWQMSSVFAIYVWLIFPIWYRIWMYYH 208

Lymnaea LAIPVGAILAVLVCICCSISKTKYKRPYPFTRRIWQMSSVASIYIWLIFPIWYRIWLYVH 208

Crassostrea LSVPIGVILGALVCICCTVSKVFYKRPYPFTRKIWQIGSVGGIYTWLSLPILHRLFLD-- 212

Sepiella WAITIGIGLGVVVCMCCSISKIRYKRPYPFVRKVWQLGSVGGIYAWLILPIAHRVFLFVI 209

Octopus WIIPVGVLLGVGVCLCCSISKVRYKRPYPFIRKVWQLGSVGGIYALLILPIAHRVLLMIL 209

Zebrafish LFLPGAAMLAWLSCASCCYSKFRYRRPYPFHRKICQIIPTSLAYLLDISPVAHRLLTK-- 237

Chicken FFLPAAAFCGWLSCAGCCYAKYRYRRPYPIMRKMCQVIPAGLAFILDISPVAHRVIVCHL 236

Human FFLPAAAFCGWLSCAGCCYAKYRYRRPYPVMRKICQVVPAGLAFILDISPVAHRVALCHL 237

Mouse FFLPAAAFCGWLSCAGCCYAKYRYRRPYPVMRKICQVVPAGLAFVLDISPVAHRVALCHL 237

: . . * * :* *:****. *:: *: . : *: :*:

Biomphalaria DGKWSSSFKNHIQQMLWFTVGGFFFGSDVPQRFCPGLFDIIGHSHQIFHMCIFMTTYEQM 268

Lymnaea TGEWDPSFKHHIRQMCWFTLGGFFFGSDIPQRFFPGTFDIIGHSHQLFHICIFMTTYEQL 268

Crassostrea KDGEDVSLYHHTSQMIWFALAGFFFGSDIPQRFFPGKFDFVGHSHQIFHICIILVTQKQL 272

Sepiella SSQWDQGLPHHMEQMVWFLVAGFFFGSDIPQRFFPGKFDFVGHSHQLFHI---------- 259

Octopus TSEWDKGLPHHIEQMIWFIMAGFFFGSDIPQRFFPGKFDFLGHSHQLFHICIMMVSWKQL 269

Zebrafish -SWDEPVLVFHAMQVAFFLLAALFFSCPVPERFFPGRCDIVGHGHQIFHIFLVLCTMCQL 296

Chicken GGCEEDAAWYHTYQILFFLISAYFFSCPVPEKYFPGSCDIVGHAHQIFHTFLAICTLSQL 296

Human AGCQEQAAWYHTLQILFFLVSAYFFSCPVPEKYFPGSCDIVGHGHQIFHAFLSICTLSQL 297

Mouse AGCQEQAAWYHTLQILFFLVSAYFFSCPVPEKYFPGSCDIVGHGHQIFHAFLSVCTLSQL 297

. . * *: :* :.. **.. :*::: ** *::**.**:**

Biomphalaria NALYLELTDDTVIIHKMEEPTLFN-TWGMLAMVIVANIFVVYYFHCSVNNRLEQEKEQNL 327

Lymnaea SALYLELTGISVIIHNMESPTLFN-TWGVLILVVVCNSIVVYLFHISVERKLEDERKKED 327

Crassostrea DGVHLDIEKYHRTPAFVDEPSFME-TFGAVISLTIVCFLNVFIFHNVVKYRLGKENVKQE 331

Sepiella ------------------------------------------------------------ 259

Octopus DGIYEDIVAWKTLLYEKDPPTFSS-TFGAIFLTIFINLIVVVIFTESAKQFIDQGKPREI 328

Zebrafish EAMFRDFLVHQQSVVDAHGEHFILLAGGSFFLLVLCSILTAVLMRGAVQRQLRKKD---- 352

Chicken EAICLDYKNRQEIFLKRHRPFSIYLSCISFFGLVACSAITAYILRCRIKAILAKKDS--- 353

Human EAILLDYQGRQEIFLQRHGPLSVHMACLSFFFLAACSAATAALLRHKVKARLTKKDS--- 354

Mouse EAILLDYQGRHEIFLQRHGPLSVYSACLSFFVLAACSAATATLLRHKVKDRLIKKDS--- 354

Biomphalaria DVKNNKHMTENGGSCLHQENIQKSLV--------SKCKDKTQ----- 361

Lymnaea KEEE-----DKGEGVLKEKAELKSNSPNRKSANGDGCDVHHRNGVIH 369

Crassostrea ----------------------------------------------- 331

Sepiella ----------------------------------------------- 259

Octopus KIDLISVKKRK------------------------------------ 339

Zebrafish ----------------------------------------------- 352

Chicken ----------------------------------------------- 353

Human ----------------------------------------------- 354

Mouse ----------------------------------------------- 354

**Supplementary Figure 10.** Multiple sequence alignment of molluscan mPRγ candidates and vertebrate mPRγ sequences. Transmembrane domains are indicated with green color.

Lymnaea ---------------------------------MLLLPATLSREEIPILFHEPHVMKGFR 27

Zebrafish -----------------------------MLNL-IKLPQVFTINQVPKVFHEDGIISGYR 30

Chicken ------------------------------MLS-LKLPRLLSIHQVPKGYQEQGILCGYR 29

Human ------------------------------MLS-LKLPRLFSIDQIPQVFHEQGILFGYR 29

Mouse ------------------------------MLS-LKLPRLFRIDQVPQVFHEQGILFGYR 29

Biomphalaria --------------------------MLGMSVSTGLSGPIYYAHQVPEHFHEHYILRGYR 34

Sepiella MATRMVIGAKTSYHFRLMMKKIISCKSFEMISTYPLNGPLYHVDQIPAEFHESFILSGYR 60

Octopus MAARIVIAARITDHIKLTLKKLIRHRNLEMMVFQRILGPLCSVEQIPTEFRENFILTGYR 60

.::* ::* :: *:*

Lymnaea PLHYPWMSYFLSLFQWHNELLNIWTHLLALIMVLVRASMWWTEFDLLRDPYMWPLSVGII 87

Zebrafish HPCSSAKDCVLSLFQLTNETLNIWTHFLPTWFFLWKLLTVVLVLEDWRDPFIWPFLVFLL 90

Chicken PPRISAADCVLSAFQMTNETLNIWTHFLPAWYFVWMLVGRLWGPG-GRDPPAWPLLAYLL 88

Human HPQSSATACILSLFQMTNETLNIWTHLLPFWFFAWRFVTALYMTDIKNDSYSWPMLVYMC 89

Mouse HPQSSATACILSLFQMTNETLNIWTHLLPFWFFVWRFMTALYVTDIQNDSYSWPMLVYMC 89

Biomphalaria HPKSSVTQCLLSVFDPTNETLNIWTHFLPTWYFVYVVYHLWWSIDFTNDVYSWPLLSYLL 94

Sepiella HPKSTFLQCVFSVFYRTNETGNFWTHFLPSCYFIYVICEK-LSE----DDYSLPFLAYLL 115

Octopus YPECTIFQCVCSIFTATNETFNFWTHFLPALYFLYTICQT-YSKDFLQSSYNSPLIAYLV 119

. * * ** *:***:* . . *: :

Lymnaea TMIILYVCSSGAHCFQNRSELVHYTCFMFDYAGIGLYGFGSTMLHYWYCLHESF-----M 142

Zebrafish SCCVYPLASSCAHTFSTMSERARHICFFFDYGALSFYSLGSAIIYSSYSFPDKW-----V 145

Chicken SCCIYPLASSCAHTFSPMSARARHVCYFFDYAALSMYSLGSALAYSAYVFPEEW-----V 143

Human TSCVYPLVSSCAHTFSSMSKNARHICYFLDYGAVNLFSLGSAIAYSAYTFPDAL-----M 144

Mouse TSCVYPLASSCAHTFSSMSKNARHICYFLDYGAVNLFSLGSAIAYSAYTFPDAL-----V 144

Biomphalaria VCCAFPLASAVAHLFNVMSDCARHVCFFLDYSALSLFSFGVALIYRAYCFPSHILSNTTG 154

Sepiella ACILFSLASSMAHMFFVLSDYARHICFFIDYGGLSLFSIGSAIAYRAYVFPEVL-----E 170

Octopus TCILFPFASSMAHMFLSLSNNARHICFFIDYGAVSLYSFGSSIAYRAYAFPEVL-----E 174

. *: ** * * .:: *:::**..:.::.:* :: : * : .

Lymnaea GSLSHQLAIPVGAILAVLVCI--CCSIS------------KTKYKRPYPFTRRIWQMSSV 188

Zebrafish NGTFHLNYVSIAVVNSIISTALACYSRLGLPFLEYNCHSIKRPSGKLDQKLCKCLRIIAF 205

Chicken GSIFHCCYVPVAVLNTVLSTSLACYSRFLE---------------LERPWLSKASRTLAF 188

Human CTTFHDYYVALAVLNTILSTGLSCYSRFLE---------------IQKPRLCKVIRVLAF 189

Mouse CSTFHECYVALAVLNTILSTGLSCYSRFLE---------------LQKPRLCKLLRVLAF 189

Biomphalaria CTWFKDHYVNLAAITAILCTFISCETRFMK-----------------PSAARKAIRLGAF 197

Sepiella RTWFGYWYLPMAIFGALASTFFSCLSRFKK-----------------DCHTQQLLRIVAF 213

Octopus YTWFGKWYLPIAFFSALLSTLFSCQSRFSG-----------------K--YEQIFRIGVF 215

: :. . :: * : : : .

Lymnaea ASIYIWLIFPIWYRIWLYVHTGEW-DPSFKHHIRQMCWFTLGGFFFGSDIPQRFFPGTFD 247

Zebrafish VYPYLFDNIPLFYRIFVCAGEGCTVNEANTVHYQHTSLAFFTGFLFATHLPERLAPGSFD 265

Chicken VYPYLFDSIPLFYRFYVCAARSCA-DPTVAAHYRHTAFAFLTCFIFATHLPERLAPGHFD 247

Human AYPYTWDSLPIFYRLFLFPGESAQ-NEATSYHQKHMIMTLLASFLYSAHLPERLAPGRFD 248

Mouse AYPYTWDSLPIFYRLFLFPGESSR-NEAMLYHQKHMGMTLLASFFYSAHLPERLAPGRFD 248

Biomphalaria AIPYLFDSIPIFYRILFPDV-NEW--SAEYLHKRQFLFALVAAFLYASHLPERLLPGIFD 254

Sepiella SLPYLFDNLPVMVWLALCDFKQCL--QSKFYHITQFMFCFIAAFLYMSHLPERLQPGRFD 271

Octopus SWPYFFDNIPIFILF--TPLEERF--YSKFYHTKQFLYCFMAVLMYVSHFPERFKPGYFD 271

* : :*: : : * : . ::: :.:*:*: ** **

Lymnaea IIGHSHQLFHICIFMTTYEQLSALYLELTGISVIIHNMESP-TLFNTWGVLILVVVCNSI 306

Zebrafish YIGHSHQLFHVFAIIGTYFQMTAIELDMAARKQWLHAHLPPVTFLNTVGAAFFSVVSGLC 325

Chicken YIGHSHQVFHVCGILGTHFQLEAILMDMSERQARLPATSLLQ----ALAPMGTCMAVGLA 303

Human YIGHSHQLFHVCVILATHMQMEAILLDKTLRKEWLLATSKPFSFSQIAGAILLCIIFSLS 308

Mouse YIGHSHQLFHVCVILATHLQMEAILLDKTLRREWLLATSRPFSFPQIAAAMLLCIIFSLS 308

Biomphalaria IIGHSHQLFHVSSILAVMDQLQAVLLDFKERRSFVEPCWQSREFSNSLGYLLNIFVINSI 314

Sepiella IWGHSHQIFHVCGILGTISQMKAIEIDMDLQKANIIKPCYYMYFHYSVEIMCAVLILNII 331

Octopus IVLHSHQLFHIFGIISTFYQMRGIQIDMATRKNTITKQWFYNYFHYSISIVLVQLLLIAL 331

****:**: :: . *: .: :: : .

Lymnaea VVYLFHISVERKLEDERKKEDKEEEDKGEGVLKEKAELKSNSPNRKSANGDGCDVHHRNG 366

Zebrafish IVYVFSLSLFSTR-GVKNKSF--------------------------------------- 345

Chicken VIAHCSAQLCRAP-EPSHREKLH----GQ------------------------------- 327

Human NIIYFSAALYRIP-KPELHKKET------------------------------------- 330

Mouse NIIYFSAALYRIP-EPELHEKET------------------------------------- 330

Biomphalaria IIMIFTIRLLYLKHKMKCT----------------------------------------- 333

Sepiella IIFIFSCML---KIEMKKKND--------------------------------------- 349

Octopus IILLYTCIL---NHKKAIKKKE-------------------------------------- 350

: :

Lymnaea VIH 369

Zebrafish --- 345

Chicken --- 327

Human --- 330

Mouse --- 330

Biomphalaria --- 333

Sepiella --- 349

Octopus --- 350

**Supplementary Figure 11.** *Lymnaea* homologs to nuclear and membrane ecdysone receptors. The homology searches were performed using *Drosophila* (AAF57278.3) and *Octopus* (XP_014782910.1) nuclear ecdysone receptors sequences, as well as using the *Drosophila* dopamine/ecdysteroid receptor sequence (ecdysone GPCR; AAF47893.1).

>*Lymnaea* nuclear ecdysone receptor homolog_cds

ATGCACGGAGAACTTTCAGACTTGTTCAGTAAATCAACAGTTAATAGTGCACACCCAGCCATGGGCAAAGTTCTCCCTGAGGGTGAGATGCCCCCAGACAGCAGCCAGATTCAGTTATCTCTAGCGGATCGGATGAGTTTCGTTCCTGGCCAGGGCAGCAGTGCTTACCATACTTTGGATAATTCTGATGAGTTAGGGGGGCCATCAGACCCCAAACGGAAGAAGGGAAATGGTGTGGAGGGCAAAAGTATAGAGGAGGAGCTGTGTAGGATTTGTGGAGACAGGGCATCAGGATACCACTACAATGCTCTCAGCTGTGAGGGATGTAAAGGTTTTTTTCGTCGAAGCATTACAAAAACTGCTGCCTATGTTTGCAAATATGGAGGCAATTGTGAGATGGATATGTGGATGCGACGAAAGTGCCAATCATGCAGGTTACGAAGATGTAGGGAAGTTGGAATGAAAGAAGAATGTCTCTTATCAGAAGACCAGTGTAAGGCCAGAGATGCCAGACGTAAAGCCAAACAGAGATTTGTGCCCAAGAAAGAAGTTCACAGCCCAGATAGTTATGGTGGCCTCAGCTCCGACATTGAGACGGTTACCCTGACCCCCAATCTGGATATTAAACCCTCATTTTTTTCCTTTTCATCCACTGCCTCCACCTCATCTGGTGCAGGGTCCCCATGCTCAGTTTCAGATGATGTCCCAGATCCTATGAAGAAGTTGACAGAGCAACAGAGAGAAATTATTGAAAAGTTGGTTGCCTTACAGGACAAGTATGAGTTTGCTGATGACGCAAGCTACGAGGAGACCTTAAAAGATTTTGATTCCCGGAAAAACCAATTATTGGATCCTCAAACCTTCATGAGCTCCCTGGCACAGACATTCGTTCTCATTACACAACTGATTGTGGAGTTTGCTAAAGGCCTGCCATGGTTCCTCACACTATCCAAAGAAGATCAAATAACTTTGCTCAAGTCATCATCAACTGAAGTGATGTCCATCAGGGCAGCCAGATGTTATGACCTAGAATCATCGTCCATTGTGTTTGGTAATGGCCAGCCATGTACCCTAGATAACATGAAAGCTGCTGGACTGGGTCATTATGCTGAGCTTCTGTATGAATTTTGTCACAATATGGCATCAATAAAGACAGATAATGCTGAATATGCCCTGTTTACAGCTATATGCATCTTCTCAGAACGGCCTGGACTAGAGGCAAAGCAGAAAGTGGAGAAAATTCAAGGTGAATATGTGGAATTGTTGAATGCATACGAGACAGCTAAACGAGGTCGTGGCGGTAAAGCTCTCGCCCGTTTCCTTGCTAGACTGACAGATTTGCGTTCCATTAGTGTAGAACACTCCAATGTGCTTGTGGAGTTGGATGTAAAGAAAGATGGCCCAGATGTTCCTTCTATCATCAAAGATATTCTTCTCTTACCCCCAGAAGGCAACTAG

>*Lymnaea* nuclear ecdysone receptor homolog_protein

MHGELSDLFSKSTVNSAHPAMGKVLPEGEMPPDSSQIQLSLADRMSFVPGQGSSAYHTLDNSDELGGPSDPKRKKGNGVEGKSIEEELCRICGDRASGYHYNALSCEGCKGFFRRSITKTAAYVCKYGGNCEMDMWMRRKCQSCRLRRCREVGMKEECLLSEDQCKARDARRKAKQRFVPKKEVHSPDSYGGLSSDIETVTLTPNLDIKPSFFSFSSTASTSSGAGSPCSVSDDVPDPMKKLTEQQREIIEKLVALQDKYEFADDASYEETLKDFDSRKNQLLDPQTFMSSLAQTFVLITQLIVEFAKGLPWFLTLSKEDQITLLKSSSTEVMSIRAARCYDLESSSIVFGNGQPCTLDNMKAAGLGHYAELLYEFCHNMASIKTDNAEYALFTAICIFSERPGLEAKQKVEKIQGEYVELLNAYETAKRGRGGKALARFLARLTDLRSISVEHSNVLVELDVKKDGPDVPSIIKDILLLPPEGN

Conserved domain analysis

*Drosophila*


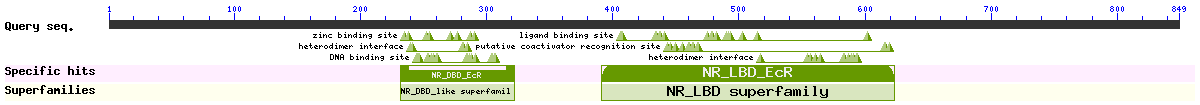


*Octopus*


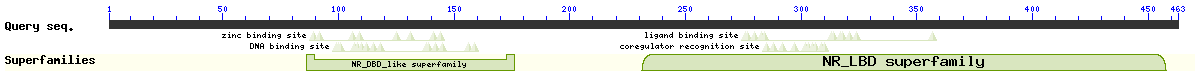


*Lymnaea*


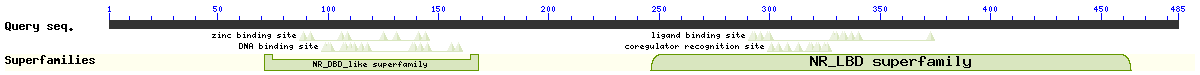


>*Lymnaea* ecdysone GPCR homolog_cds

ATGGGCCACCGTCCAGGTTTGAACACATCATTCGTCGTCTTTGGCCTGCTGGTAGCAGTGGTAGCGGGGTCACAGCTTCCAACAATCAAAAGATCGACGTCTACCGCCGCCCTGTCCACAACGGCATCGCTGGAAGGGGGGATGTCCAGAAATTTCAACGCCGTCACGGACATGGCCGACGACCCCAAGGGGGACAACCCGGAGTTCACCATCCAGACGTTCATCACCATACTCGTGACCATCCTGATCGTGGTCAGCAACTGCGCCATCATCATGGTGGCGTCGTGGACCGAGGCGTTCAGCAACTTCAACAAGACCTTCATCTACTCGCTGACCCTGGCGGACCTGCTGATTGGCCTGTTCATCACGCCGTACTCCATCTTCCTGTCCGTCTACAAGAGATGGGTCTACACGAGTGATATGTTCTGTAGCGTGGAGGCTTATGTTTTCACCACGCTCATGACGGCCAAGCTGTACTCTCTGACCTGGCTCAACGTCGACCACTACGTGGCGGTGCGCAAGCCCGAGCGGTACAACGTGATGATGTCCCCTACTCGAAGTCTCTGCTGGATCGTCTTCTCCTGGATCGTGGCGGTCTCGTTCTGCTGTCCGCCGCTCTTCTCTTTTCAGTCGGCTGTCTTCAACAAAGCCACGTCGATATGCATGATCGATACCAAGCAACAAGTAGCCTACATGCTGACCGCTGGCGTGCTGGTATCGATACCAAGCTTATTTGCTATGGCCATAACCAGCGCCTACTTATTTACCAAGTCTTTCAAGAAACGGATCCAATTCTATGAGAAAGTTTATGTGGAGCTGTCATCACGACCGAGGAACTACTACATCACCTGTATCATGTGTCTGGTCTTTACCCTAGTCTGGGTGCCCTTTGTTATCATCAACCTTGTGGTCAAGCGGGTATTCCAGGCGGAGGTGGACGACTTTCTCCAGTTCTTCAGCTGCTGGCTGGGAATCGCCGCCAGTTTCAGCCAGTTCTTCATACTGCTGCTGCTGAGCGCCGACTTCCGGTTCCACTTCTGGGAGGTCTGCCAGCTGCCGGGCTGGTGCACGCCGTGCAGCAGGGGTCATCTGGCGGAGAGGGTTGACGCCAACATCATCACCACGCCCCACAAGCGACGACGCGGGCAGAAGGTGTGA

>*Lymnaea* ecdysone GPCR homolog_protein

MGHRPGLNTSFVVFGLLVAVVAGSQLPTIKRSTSTAALSTTASLEGGMSRNFNAVTDMADDPKGDNPEFTIQTFITILVTILIVVSNCAIIMVASWTEAFSNFNKTFIYSLTLADLLIGLFITPYSIFLSVYKRWVYTSDMFCSVEAYVFTTLMTAKLYSLTWLNVDHYVAVRKPERYNVMMSPTRSLCWIVFSWIVAVSFCCPPLFSFQSAVFNKATSICMIDTKQQVAYMLTAGVLVSIPSLFAMAITSAYLFTKSFKKRIQFYEKVYVELSSRPRNYYITCIMCLVFTLVWVPFVIINLVVKRVFQAEVDDFLQFFSCWLGIAASFSQFFILLLLSADFRFHFWEVCQLPGWCTPCSRGHLAERVDANIITTPHKRRRGQKV

Conserved domain analysis

*Drosophila*


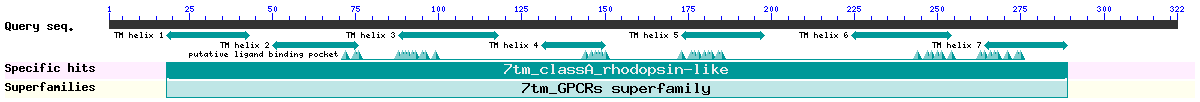


*Lymnaea*


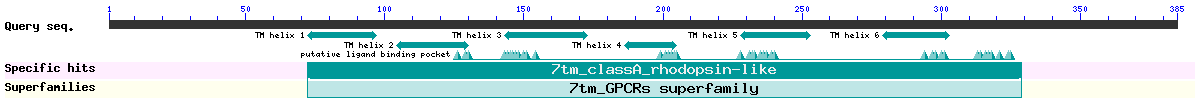


**References**

1. Pang Z, Lu Z, Wang M, Gong L, Liu B, Jiang L, et al. Characterization, relative abundances of mRNA transcripts, and subcellular localization of two forms of membrane progestin receptors (mPRs) in the common Chinese cuttlefish, *Sepiella japonica*. *Anim Reprod Sci* (2019) 208:106107. doi: 10.1016/j.anireprosci.2019.106107

2. Ren J, Chung-Davidson YW, Jia L, Li W. Genomic sequence analyses of classical and non-classical lamprey progesterone receptor genes and the inference of homologous gene evolution in metazoans. *BMC Evol Biol* (2019) 19:136. doi: 10.1186/s12862-019-1463-7

3. Kumar S, Stecher G, Tamura K. MEGA7: Molecular Evolutionary Genetics Analysis Version 7.0 for Bigger Datasets. *Mol Biol Evol* (2016) 33:1870-4. doi: 10.1093/molbev/msw054

4. Thomas P, Pang Y, Dong J, Groenen P, Kelder J, de Vlieg J, et al. Steroid and G protein binding characteristics of the seatrout and human progestin membrane receptor alpha subtypes and their evolutionary origins. *Endocrinology* (2007) 148:705-18. doi: 10.1210/en.2006-0974

5. Fujimoto K, Totani Y, Nakai J, Chikamoto N, Namiki K, Hatakeyama D, et al. Identification of Putative Molecules for Adiponectin and Adiponectin Receptor and Their Roles in Learning and Memory in. *Biology (Basel)* (2023) 12:375. doi: 10.3390/biology12030375.

6. Wellendorph P, Hansen KB, Balsgaard A, Greenwood JR, Egebjerg J, Brauner-Osborne H. Deorphanization of GPRC6A: a promiscuous L-alpha-amino acid receptor with preference for basic amino acids. *Mol Pharmacol* (2005) 67:589-97. doi: 10.1124/mol.104.007559
